# Genome-wide association studies of body weight, body conformation traits and coat color in Gamba sheep

**DOI:** 10.5713/ab.260295

**Published:** 2026-06-15

**Authors:** Yangshen Zhang, Hongbin Zhou, Luobu Gesang, Duoji Zhaxi, Ciren Pubu, Hong Miao, Jipan Zhang, Yongju Zhao

**Affiliations:** 1 College of Animal Science and Technology, Southwest University, Chongqing, China; 2 Chongqing Key Laboratory of Herbivore Science, Chongqing, China; 3 Chongqing Engineering Research Center of Higher Education for Herbivore, Chongqing, China; 4 Gamba County Agriculture and Animal Husbandry Comprehensive Service Center, Shigatse Tibet, China

**Keywords:** Body Conformation Traits, Coat Color, Correlation Analysis, Genome-wide Association Studies, Tibetan Sheep

## Abstract

**Objective:**

Elucidating the genetic architecture of body weight (BW), body conformation, and coat color (CC) is vital for the precision breeding of Gamba sheep, a unique high-altitude indigenous breed. Although previous studies focused on general plateau adaptation, the specific genetic factors regulating economic traits in this extreme-altitude population remain poorly understood.

**Methods:**

We characterized six key phenotypic traits and conducted genome-wide association studies (GWAS) leveraging whole-genome resequencing data from 296 individuals. To ensure robustness, we systematically evaluated five statistical models. The SUPER model was selected for growth-related traits due to its superior power in balancing signal sensitivity and false-positive control, whereas the MLMM model was applied to the binary CC phenotype. Both models consistently yielded genomic inflation factors (λ) close to the theoretical expectation.

**Results:**

Phenotypic analysis revealed distinct sexual dimorphism in growth determinants: cannon bone circumference was the primary driver influencing BW in rams, whereas body height and heart girth were predominant in ewes. The GWAS identified 82 genome-wide significant SNPs, delineating a prominent novel genomic region on chromosome 5 associated with body height. Functional annotation highlighted *PRKCE* and *SGIP1* as plausible positional candidate genes for growth characteristics, while *SPIRE2* was pinpointed as a primary genomic driver regulating CC variation.

**Conclusion:**

These findings provide novel insights into the genetic blueprint governing growth and pigmentation in this unique livestock, offering precise molecular targets for future marker-assisted selection.

## INTRODUCTION

Gamba sheep (*Ovis aries*) are an indigenous breed distributed exclusively in Gamba County, Tibet Autonomous Region, China. Situated at an extreme altitude of 4,700 to 5,200 meters above sea level, this region is characterized by chronic hypoxia and severe cold. Through long-term adaptation to these harsh conditions, Gamba sheep have developed outstanding meat production performance and remarkable plateau resilience, serving as an essential pillar of the local livestock industry [1,2]. Recent domestic studies have primarily focused on their plateau adaptation, gut microbiota, and meat quality characteristics. For instance, Jize et al. [3] reported that different feeding strategies significantly influence growth performance and rumen microbial communities, while Xu et al. [4] noted that Gamba sheep possess higher glutamic acid content and richer trace elements compared to other Tibetan sheep breeds, contributing to superior nutritional value.

To date, genome-wide association studies (GWAS) and genomic selection scans have been extensively utilized to decipher the genetic basis of adaptive, reproductive, and economic traits across various ruminant populations [5,6]. For instance, recent comprehensive genomic analyses have successfully revealed candidate genes for body size in Hu sheep [7] and identified critical loci related to the lambing trait in Hotan sheep [8]. Furthermore, genomic analyses in Tibetan yaks have revealed convergent evolutionary signatures in pathways regulating body size [9], while studies in other Tibetan sheep subpopulations have identified specific loci associated with growth (e.g., *LCORL-NCAPG*) [10] and coat color (CC) variation (e.g., *ASIP* and *MC1R*) [11]. Residing at altitudes significantly higher than most other Tibetan sheep populations, they likely possess a unique genetic architecture for body conformation and pigmentation that differs from broader regional groups.

Body conformation traits—including body weight (BW), body height (BH), body length (BL), heart girth (HG), and cannon bone circumference (CB)—are of paramount economic importance as they serve as direct indicators of growth performance and carcass yield [12,13]. Notably, emerging evidence suggests a biological link between pigmentation and body size traits [14]. Under extreme high-altitude conditions, melanin-related pathways may interact with metabolic networks to optimize energy allocation, where CC variation potentially influences thermoregulation and ultraviolet protection [15,16]. Therefore, uncovering the polygenic nature of these traits is vital for precision breeding and for enriching the global map of sheep genetic resources.

To overcome the inherent biases of traditional SNP chips and detect population-specific variants unique to this indigenous breed, whole-genome resequencing (13×) has become an essential approach [17]. Compared to preselected arrays, resequencing significantly enhances statistical power and mapping resolution, allowing for the precise identification of novel causal variants [18]. Although Gamba sheep were officially included in the National Livestock Genetic Resources Catalogue in 2022, the lack of high-resolution genomic data has severely hindered progress in their conservation and genetic improvement. In this study, we characterized the CC, BW, and four conformation traits (BH, BL, HG, and CB) of 301 Gamba sheep by leveraging 13× whole-genome resequencing data. Phenotypic relationships and sexual dimorphism were examined through correlation and path analyses. To ensure the accuracy of our genomic mapping, five statistical models (general linear model [GLM], mixed linear model [MLM], compressed mixed linear model [CMLM], multi-locus mixed linear model [MLMM], and the settlement of MLM under progressively exclusive relationship [SUPER] model) were systematically evaluated across different trait categories. Consequently, a tailored modeling framework was adopted: the SUPER model was selected for growth characteristics due to its superior control of population stratification, whereas the MLMM model demonstrated the optimal fitting performance for the (CC) phenotype, both maintaining the genomic inflation factor (*λ*) near 1.0. These findings elucidate the genetic basis of growth and pigmentation in Gamba sheep and provide essential genomic targets for marker-assisted selection (MAS).

## MATERIALS AND METHODS

### Experimental animals and trait measurement

A total of 301 healthy adult Gamba sheep (123 rams and 178 ewes, aged 1–5 years) were randomly selected from Gamba County, Tibet, for phenotypic measurement. To ensure data reliability, strict health screenings were conducted to exclude individuals with clinical diseases or disabilities. Although the sex ratio reflects the natural flock structure of the region, sex was incorporated as a covariate in the statistical models to mitigate potential sampling bias. To minimize environmental noise, all animals were maintained under uniform traditional alpine grazing management on natural pastures without supplemental feeding. Sampling was completed during a single autumn grazing season within the same ecological niche, effectively controlling for seasonal variables such as temperature and forage availability. All procedures were performed with the informed consent of the animal owners.

To better align with the biology of melanogenesis, CC was treated as a binary categorical trait. We constructed a strict case-control association model where solid black individuals were defined as cases and solid white individuals as controls. Individuals with intermediate or piebald phenotypes were excluded to prevent phenotypic confounding, resulting in a sub-cohort of 116 individuals strictly for the CC GWAS.

*BL:* straight-line distance from the anterior point of the shoulder to the posterior end of the ischial tuberosity.

*BH:* vertical distance from the highest point of the scapula to the ground.

*HG:* circumference around the chest immediately behind the scapula.

*CB:* horizontal circumference at the narrowest point of the left foreleg cannon bone.

BW and body conformation traits (BH, BL, HG, and CB) were measured by a single trained team using standardized instruments to minimize inter-observer bias. Notably, live BW was recorded following a 12-hour fasting period to ensure consistency. Following measurements, jugular venous blood was collected into EDTA tubes and stored at −20°C for genomic DNA extraction.

Prior to association analysis, phenotypic data underwent rigorous quality control. Outliers deviating by more than three standard deviations (±3 SD) from the population mean were identified and subsequently removed (n = 5 individuals) to mitigate experimental noise. Descriptive statistics and distribution analyses—including boxplots, histograms, and kernel density curves—confirmed that all remaining traits followed a normal distribution (Supplements 1–5) [19].

### DNA extraction and quality assessment

Genomic DNA was extracted from the collected blood samples using a commercial extraction kit (QT-1001; IGENEBOOK) and stored at −20°C awaiting whole-genome resequencing. DNA concentration, integrity, and purity were evaluated using 1.5% agarose gel electrophoresis and a NanoDrop spectrophotometer (Thermo Fisher Scientific). DNA samples that met the quality requirements were sequenced on the DNBSEQ-T7 platform.

### Genome alignment and variant detection

Approximately 12.3 TB of whole-genome resequencing data from 301 Gamba sheep were utilized, achieving an average depth of 13× and 99.6% genome coverage [20]. Following alignment to the ARS-UI_Ramb_v3.0 reference, initial variant calling yielded ~48.7 million SNPs (Supplement 6). To ensure high-quality associations, 9,785,979 SNPs were retained after stringent filtering (DP≥3; missing≤0.1; minor allele frequency [MAF]≥0.01, and Hardy–Weinberg equilibrium [HWE] p>10^−6^ [21]). The 13× sequencing depth provided high confidence for genotype calling across both homozygous and heterozygous sites, ensuring sufficient sensitivity for detecting QTLs associated with complex traits while minimizing spurious associations from rare variants (MAF<0.01) [22].

### Correlation and path analysis

To account for the observed sexual dimorphism in Gamba sheep, phenotypic correlation and path analyses were conducted separately for rams and ewes. First, Pearson correlation coefficients were calculated to evaluate the bivariate linear relationships between BW and various body conformation traits. To further elucidate trait relationships and partition these correlations into direct and indirect pathways of influence, a path analysis was performed. In this model, BW was defined as the dependent variable, while the conformation traits served as independent variables. The direct path coefficients were estimated as standardized partial regression coefficients derived from a multiple linear regression model. The indirect effects were subsequently computed by multiplying the respective correlation coefficients by the direct path coefficients. All statistical analyses were implemented in R, and the results—including correlation matrix heatmaps and path diagram models—were visually rendered to clearly illustrate the patterns of trait associations and causal interactions.

### Principal component analysis and population structure analysis

Principal component analysis (PCA) of the 301 Gamba sheep was conducted using *TASSEL* software to evaluate population structure. Although the first 10 principal components (PCs) were included as covariates in the subsequent GWAS models to rigorously correct for population stratification, only the first two components (PC1 and PC2) were visualized to illustrate the genetic clustering patterns. Population structure analysis was further performed using *ADMIXTURE* software, which applies a Bayesian model-based clustering approach to infer ancestral genetic components. The optimal number of ancestral clusters (K) was determined based on the cross-validation error. All results were visualized using RStudio. Additionally, a population phylogenetic tree was constructed (Supplement 7).

### Kinship and linkage disequilibrium analysis

Kinship coefficients among individuals were calculated using *TASSEL* and subsequently used to construct the kinship matrix (Supplement 8). Linkage disequilibrium (LD) decay analysis was performed using PLINK. The average LD decay distance was determined at the point where the squared correlation coefficient (r^2^) dropped to 0.2, which served as the baseline for defining the genomic extent of linkage within the Gamba sheep population. Haplotype blocks were subsequently visualized using LDBlockShow. These analyses were utilized to identify tag SNPs and define the genomic intervals for candidate gene selection. Specifically, genes located within the LD blocks containing significant SNPs, or within a ±100 kb window flanking these markers, were prioritized as candidate genes. Given the rapid LD decay observed in our population (7.99 kb), extending the search space to this conservative 100-kb window ensures the comprehensive capture of potential cis-regulatory elements (cREs) and structural variants. This interval selection is consistent with established methodologies widely adopted in previous whole-genome association studies in sheep [23,24].

### Genome-wide association study

Genome-wide association analyses were performed using the GAPIT v3.0 R package [19]. To minimize potential confounding factors, only autosomal SNPs were included in the analysis, while those located on sex chromosomes were excluded. To determine the most robust statistical approach for this population, we systematically evaluated five models: the GLM, MLM, CMLM, MLMM), and the SUPER model [25]. The fitting performance of each model was assessed using quantile-quantile (Q-Q) plots and the genomic inflation factor (*λ*) [26]. Based on the trait-specific architectures, a tailored strategic modeling framework was adopted: the SUPER model was selected as the optimal framework for continuous growth and body conformation traits due to its superior power in controlling population stratification, whereas the MLMM model was implemented as the core framework for the binary CC phenotype to maximize detection sensitivity for major-effect loci while maintaining *λ* values closest to the theoretical expectation (*λ*≈1.0) (Supplements 9–14). For continuous growth traits, the SUPER model improves statistical power and computational efficiency by constructing a kinship matrix through a three-step algorithm: (1) preliminary screening to sort SNPs by association; (2) determining the optimal number and size of marker bins to select “pseudo-QTNs” using maximum likelihood; and (3) excluding pseudo-QTNs in LD with the testing marker to derive a complementary kinship matrix [27]. The standard single-locus equation of the mixed model within the SUPER framework is as follows:


(1) y=Xβ+Zu+Wv+e

Where: *y* represents the vector of phenotypic observations; *v* is the vector of the marker effect being tested; *W* denotes the incidence matrix linking individuals to the specific marker *v*; *β* is the vector of fixed effects, which incorporated sex, age, and the first three PCs to rigorously account for individual variability and population stratification; *X* denotes the design matrix associated with the fixed effects *β*; *u* represents the vector of unknown random polygenic effects; *Z* is the incidence matrix relating individuals to the random genetic effects *u*; *e* is the vector of random residual errors. The random effects are assumed to follow a distribution of *u* ~ *N* (0, G), where G = 2*Kσ^2^_a_*, with *K* being the kinship matrix derived from the selected pseudo-QTNs and *σ^2^_a_* being the additive genetic variance.

Conversely, for the binary CC phenotype, the MLMM model was utilized to address the limitations of single-locus models when dealing with major-effect categorical traits. MLMM applies a stepwise forward-backward linear regression approach to systematically integrate multiple associated SNPs into the mixed model as fixed cofactors, effectively preventing the inflation of Type I errors caused by confounding major QTLs [28]. The iterative MLMM model is formulated as follows:


(2) $$y=X\beta +\sum_{i\in S}M_{i}\alpha_{i}+Z_{j}v_{j}+Zu+e$$

Where: y represents the vector of binary CC observations; S is the selected set of pseudo-QTNs integrated as fixed cofactors from previous steps, with *M_i_* and *a_i_* representing their design matrix and additive genetic effects, respectively; *Z_j_v_j_* represents the effect of the specific marker under evaluation; and the other parameters remain consistent with the background mixed model definitions. The optimal iteration model was determined using the extended Bayesian Information Criterion (eBIC) to prevent model overfitting.

Given the extreme marker density of WGS data and the rapid LD decay (7.99 kb) observed in Gamba sheep, applying a standard Bonferroni correction based on the absolute number of SNPs would be overly conservative and severely inflate Type II errors. To rigorously control for false positives while maintaining appropriate statistical power, we estimated the effective number of independent tests (N_e_) via LD pruning in PLINK (--indep-pairwise 50 5 0.2). This analysis identified 1,461,988 independent chromosomal segments. Consequently, the strict genome-wide significance threshold was adjusted to p = 0.05/N_e_ (3.42×10^−8^), and a suggestive significance threshold was established at p = 1/N_e_ (6.84×10^−7^). While the strict threshold rigorously minimizes false positives, its highly conservative nature increases the risk of Type II errors for polygenic traits. The suggestive threshold, which permits an expectation of one false positive per genome-wide scan, was thus adopted to strike an optimal balance—maintaining statistical stringency while effectively capturing functionally relevant loci with moderate effect sizes. These thresholds were visually represented by red solid and blue dashed horizontal lines, respectively, in the Manhattan plots.

To formally assess the statistical detection capacity of our GWAS framework given the cohort size (N = 296), a post-hoc statistical power analysis was conducted under the MLM; assumptions using the G*Power library (v3.1.9.7) and R environment. The statistical power was modeled across a realistic range of effect sizes (expressed as phenotypic variance explained, PVE, %) at the established genome-wide suggestive significance threshold (a = 6.84×10^−7^).

The PVE by each significant SNP was calculated to evaluate its contribution to the phenotypic variation. The PVE was estimated using the following standard approximation formula:


(3) $$PVE=\frac{2{\hat{\beta }}^{2}MAF(1-MAF)}{2{\hat{\beta }}^{2}MAF(1-MAF)+{\left(SE\right)}^{2}\cdot 2N\cdot MAF(1-MAF)}$$

where *β̂* is the estimated effect size of the minor allele, MAF is the minor allele frequency, *SE* is the standard error of the effect size estimate, and N is the total sample size used in the association analysis.

### Candidate gene annotation and functional enrichment analysis

Significant SNP loci were annotated using ANNOVAR software to identify the corresponding genes. Genes located within the LD regions (±100 kb) surrounding the significant SNPs were selected as candidate genes based on the results of LD analysis. The resulting gene set was subjected to Gene Ontology (GO) functional annotation and Kyoto Encyclopedia of Genes and Genomes (KEGG) pathway enrichment analyses to explore the biological functions and molecular pathways associated with the target traits [29,30]. Venn diagrams were also constructed to visualize the overlaps among candidate gene sets associated with different phenotypic traits.

To control the false positive rate arising from multiple simultaneous tests, the raw p-values for both GO terms and KEGG pathways were adjusted using the Benjamini-Hochberg (BH) correction method to calculate the false discovery rate (FDR). The entire set of protein-coding genes in the sheep reference genome (ARS-UI_Ramb_v3.0) was utilized as the background population. A significance threshold of Adjusted p-value (FDR)<0.05 was applied to identify statistically enriched terms and pathways [31].

## RESULTS

### Descriptive statistics

A statistical analysis of CC, BW, and body measurement traits in 301 Gamba sheep was done. The mean, maximum, minimum, SE, and coefficient of variation (CV) for all phenotypes are summarized in Table 1. Among the measured traits, BH exhibited the lowest variability (CV = 10.56%), while BW showed the highest variability (CV = 29.89%). Regarding pigmentation, the population included 26 individuals with pure white coats, 185 with black-and-white (piebald) coats, and 90 with pure black coats.

The correlation and path analyses of BW and body conformation traits in Gamba sheep revealed pronounced sexual dimorphism. To account for the influence of physiological differences between sexes on trait interrelationships, rams and ewes were analyzed separately.

All body measurements in rams exhibited moderate to high positive correlations with BW (R = 0.54 to 0.68) (Figure 1A). The strongest correlation was between BH and BW (R = 0.68), followed by CB (R = 0.64). Notably, there were moderate intercorrelations among the body measurements, e.g., BH and BL (R = 0.63) and BH and HG (R = 0.62), suggesting coordinated variation among these traits. Path analysis demonstrated that CB exerted the most significant direct positive effect on BW, with a path coefficient of 0.588, followed by HG (0.360) and BH (0.283). BL had only a minimal direct effect (0.004) (Figure 1C).

In contrast, BW exhibited moderate positive correlations with BH (R = 0.54) and HG (R = 0.54), a weaker correlation with BL (R = 0.32), and almost no correlation with CB (R = 0.06) in ewes (Figure 1B). Path analysis indicated that BH and HG had the strongest direct positive effects on BW, with path coefficients of 0.418 and 0.414, respectively. BL exhibited a negligible direct effect (0.015), while CB exerted a weak negative direct effect (−0.166) (Figure 1D).

### Genome-wide association analysis

PCA results revealed that the first two PCs explained 1.23% (PC1) and 0.92% (PC2) of the total genetic variance (Figure 2A). These relatively low percentages likely reflect a high degree of genetic homogeneity within the Gamba sheep population, which has experienced long-term isolation and adaptation to the extreme high-altitude environment of the Tibetan Plateau. While these values represent a small fraction of the total variance, they effectively capture the primary population stratification within this Gamba sheep population. To rigorously correct for this stratification in the subsequent GWAS analysis, the first 10 PCs were included as covariates in the model, although only PC1 and PC2 are visualized here.

Population structure analysis indicated that the cross-validation error reached its minimum at *K* = 3, suggesting that the genetic structure of the population was best explained by three ancestral clusters (Figures 2B, 2C). Additionally, Kinship matrix analysis revealed low relatedness among most samples, with approximately 90%–95% of pairwise kinship coefficients close to zero. Prior to analysis, individuals were identified as phenotypic outliers (deviating by more than ±3 SD from the population mean) and were excluded from the dataset to enhance mapping accuracy.

A total of 82 SNPs were identified as significantly associated with continuous growth and binary CC traits in the GWAS by surpassing the customized genome-wide independent-test thresholds (Figures 3, 4). A prominent and previously unreported genomic candidate region was highlighted on chromosome 5 associated with BH. The Q−Q plot under the null hypothesis (*λ_BW_* = 1.02) showed a clear deviation from the expected distribution, indicating that the model effectively accounted for the false-positive associations.

The region associated with BH was identified on chromosome 5. It spanned a 30.376−30.397 Mb region and contained 7 significant SNPs (Figure 3B). Other significant SNPs were distributed as isolated loci across the genome. BW was associated with 24 significant SNPs (Figure 3A), BH with 12 significant SNPs (Figure 3B), BL with 8 significant SNPs (Figure 3C), CB with 10 significant SNPs (Figure 4A), HG with 13 significant SNPs (Figure 4B), and CC with 15 significant SNPs (Figure 4C).

Functional annotation of the 82 significant SNPs using the ANNOVAR software revealed 35 associated positional candidate genes (Table 2). Notably, only two variants were identified within the exonic regions of their respective candidate genes (*UNC13B* and *GM2A*). Conversely, the vast majority of the remaining variants were positioned within the intronic or intergenic regions. To further investigate the regulatory potential of these non-coding variants, we conducted a coordinate-overlap analysis against the sheep cRE atlas [32]. Our analysis revealed no direct coordinate overlap between these GWAS signals and the annotated cREs provided by the current genomic resource.

Given that several genome-wide significant loci exhibited relatively low MAFs (≦0.05), we performed a rigorous individual-level genotype-phenotype validation to rule out potential statistical artifacts driven by phenotypic outliers. As illustrated in Supplement 15, individual scatter-boxplots for these low-MAF loci demonstrated that the minor allele carriers (ranging from 6 to 17 individuals) possessed phenotypic values entirely within the normal biological range of the Gamba sheep population. The significant associations were clearly driven by a consistent shift in the phenotypic distribution of the alternative genotype groups rather than leverage effects from isolated extreme outliers, thereby validating the statistical robustness of these rare variant associations.

Based on the formal power analysis (Supplement 16), the minimum detectable effect size (PVE) required to achieve the conventional 80% statistical power in this study was determined to be 10.1%. While the current sample size (N = 296) provides sufficient power to robustly capture loci with large-to-moderate genetic effects, we acknowledge a non-trivial risk of Type II error for low-frequency variants with minor phenotypic impacts. This statistical threshold provides a transparent baseline for assessing the completeness and coverage of our genomic findings in the Gamba sheep population.

### Linkage disequilibrium analysis

LD decay analysis revealed a rapid decrease in the average LD coefficient (r^2^) with increasing physical distance. The r^2^ value declined to half of its initial value (half-decay distance) at 7.99 kb and then dropped below 0.1 by 31.17 kb, indicating that LD had nearly disappeared beyond this distance (Supplement 17). LD block visualization showed strong local linkage (r^2^≈1) within the highlighted region on chromosome 5 (associated with BH), forming a clear haplotype structure and thereby enhancing the reliability of this association signal (Figure 5).

### Gene annotation and functional enrichment

A total of 446 candidate genes (Supplement 18) were annotated using ANNOVAR software within a ±100 kb window surrounding the significant SNPs across all analyzed traits. Detailed results for GO and KEGG pathway enrichment analyses are provided in Supplements 19, 20, respectively. GO and KEGG enrichment analyses revealed that these genes were primarily involved in critical biological processes and pathways crucial for the distinct phenotypes of Gamba sheep (Figure 6). For growth and body conformation traits, key terms including regulation of growth, skeletal muscle satellite cell migration, glycine transport, and beta-alanine metabolism were significantly enriched, driven by core genes such as *SIN3A, PPM1J, RHOC*, and *GADL1*. Regarding CC regulation, genes like *MC1R* and *TYRP1* significantly populated the melanin biosynthetic process and phenol-containing compound biosynthetic process. Crucially, a clear signature of high-altitude adaptation was observed through the enrichment of oxygen/stress-related pathways, including glutathione metabolism, lactate transmembrane transport, and signal transduction in response to DNA damage (mediated by *GGT5, SLC16A1, ATM*, and *CHEK1*). Furthermore, adaptive immunity pathways, particularly regulation of T cell mediated immunity and hematopoietic cell lineage, were heavily enriched, highlighting the robust immunological fitness of Gamba sheep under extreme Himalayan environments.

## DISCUSSION

The application of correlation and path analyses is central to unravelling the intricate ties between production and body conformation traits in livestock [33]. Our results highlight a clear sexual dimorphism in how different phenotypes drive BW—the primary production trait in this study [34]. In rams, CB emerged as the chief direct determinant of BW; in contrast, BH and HG played a far more dominant role in ewes. This shift suggests that trait–weight relationships are sex-dependent, likely reflecting divergent physiological priorities between the two. Notably, this sex-specific association aligns closely with the findings of Getachew et al. [35] in Menz sheep, confirming that skeletal traits such as CB may serve as potential “phenotypic markers” with high value for male selection. This emphasizes the importance of systematically evaluating genetic parameters and trait correlations for pre-weaning and adult growth traits under local specific environmental conditions, as recently demonstrated in other breeds like Dorper sheep [36]. Therefore, CB in rams and BH/HG in ewes might serve as useful phenotypic indicators for sex-specific selection strategies. However, given that these associations are based primarily on phenotypic correlations, further quantitative genetic evaluations are required to confirm their actual efficacy in accelerating genetic gain.

Through GWAS, we identified 82 significant SNPs linked to growth and pigmentation. By setting a MAF threshold at 0.01, we were able to capture low-frequency variants that may represent the unique genetic signatures of this indigenous population [37]. Since most of these variants are intronic, we cross-referenced them with a recent sheep cRE atlas. Lacking direct coordinate overlap, we conservatively define these non-coding loci as positional candidate variants. To link these loci to specific target genes, we applied a ±100 kb window. Given the rapid LD decay (~31 kb at r^2^<0.1) in this population, this range effectively captures functional candidate genes while minimizing interference from unrelated neighboring genes [38,39].

Regarding BW, the genomic region spanning the *SGIP1* gene locus appears to be a noteworthy positional candidate, given its documented physiological role in energy homeostasis. This gene has been characterized as a potential node in the neuroendocrine control of energy intake in resilient ruminant populations [40]. Interestingly, similar metabolism-centric evolutionary strategies have been observed in other hardy breeds, such as the Baluchi sheep, where growth-related selection signals frequently link back to metabolic efficiency rather than simple muscle hypertrophy [12,13]. Our findings on OAR1 (42.36 Mb) span the intronic region of the *SGIP1* gene. Given the intronic nature of these variants, they are highly unlikely to alter protein structures directly; instead, they may act as potential regulatory elements (such as cis-acting factors) that fine-tune *SGIP1* transcription, thereby emerging as a plausible driver for the observed selection signatures on this chromosome.

Complementing these findings, *PRKCE* (OAR3: 78.53 Mb) mapped within gene ontologies associated with insulin signaling and glucose homeostasis. Prior literature indicates that *PRKCE*-mediated signaling activates mitochondrial metabolism and enhances adipose insulin sensitivity [41]. Under the chronic hypoxia of the Tibetan Plateau (4,700–5,200 m), adaptive selection targeting regulatory variants near *PRKCE* likely serves to optimize glucose uptake—a mechanism documented in other high-altitude adapted species. Nevertheless, mechanistic functional validations remain warranted to experimentally bridge this genetic association with its underlying physiology in Gamba sheep [42].

As previously indicated, genes related to structural morphogenesis and vascular integrity were significantly enriched at the nominal level (p<0.05). This functional category is mainly represented by *NTN4* and *EPB41L3. NTN4* is essential for vascular maturation and the stability of the microvasculature, which orchestrates the development of the thoracic cavity. Since HG is highly correlated with cardiopulmonary capacity, the identification of *NTN4* suggests a genetic basis for enhanced oxygen transport efficiency—a vital adaptation for survival in extreme environments [43]. Similarly, the *EPB41L3* gene regulates adipocyte differentiation via the Wnt/*b* -catenin signaling pathway [44]. Notably, genomic analyses in Tibetan yaks have revealed that selection signatures in lipid metabolism pathways (e.g., *PPARG* and *ADIPOQ*) are essential for optimizing energy partitioning and subcutaneous fat storage, providing a critical buffer against the extreme cold and seasonal nutritional stress of the plateau [9].

Most notably, a pivotal, highly significant association peak was identified on OAR14 (14.22 Mb, p = 2.31×10^−26^) mapping to the intronic region of the *SPIRE2* gene, exhibiting a robust positive effect (*β* = 0.25). *SPIRE2* encodes an actin-nucleating protein essential for the intracellular positioning and actin-dependent transport of melanosomes—the specialized organelles within which melanins are synthesized and distributed [45]. Remarkably, our GWAS mapping aligns perfectly with recent population genetics evidence in the same indigenous breed; Zhang et al. [46] identified *SPIRE2* as a core under-selection gene regulating wool color divergence between black and white Gamba sheep using selection signature scans. The convergence of our dense statistical association and their independent selective sweep signals strongly implies that variations in intracellular melanosome transport logistics, rather than conventional raw chemical synthesis pathways alone, play a decisive role in defining the distinct pigmentation patterns and patches observed in Gamba sheep.

The significant SNPs identified here—especially those on OAR1, OAR5, and OAR12 that explain over 5% of the phenotypic variance—hold immediate practical value [25]. These loci could be integrated into MAS using low-cost KASP markers for early-stage screening [47]. Alternatively, they could serve as fixed effects in weighted GBLUP (wGBLUP) models to refine the accuracy of genomic predictions [48]. Such “precision breeding” strategies offer a way to improve growth performance while ensuring the breed’s hard-won resilience to extreme high-altitude environments is preserved.

## CONCLUSION

This study establishes a high-density genomic map of production, body conformation, and CC traits in Gamba sheep, offering novel insights into the genetic framework underlying their ecological adaptation and economic characteristics. Our findings highlight a distinct sexual dimorphism in the phenotypic architecture of BW and identify 82 genome-wide significant SNPs through a comprehensive evaluation of multiple statistical models, leading to the mapping of a prominent novel genomic region on chromosome 5 associated with BH. The positional identification of key candidate genes shifts the understanding of Gamba sheep development, demonstrating that growth-related loci such as *PRKCE* and *SGIP1* underline a metabolism-driven adaptive process. This mechanism prioritizes energy homeostasis and glucose efficiency over simple structural muscle hypertrophy, a physiological trade-off that appears pivotal for maintaining homeostatic stability under the chronic hypoxia and extreme stress of the Tibetan Plateau. Furthermore, our rigorous categorical analysis pinpointed *SPIRE2* as the primary genomic driver regulating CC variation, indicating that variations in intracellular melanosome transport logistics play a decisive role in defining the distinct pigmentation patterns of this indigenous population.

While these markers offer promising targets for future MAS and precision breeding, we acknowledge that our cohort size (N = 296) primarily captures moderate-to-large effect loci, introducing a non-trivial risk of Type II errors for complex polygenic traits with minor phenotypic impacts. Furthermore, the prevalence of these variants within intronic non-coding regions suggests that the phenotypic diversity of Gamba sheep is predominantly governed by complex transcriptional regulatory networks rather than direct structural protein modifications. Consequently, the genomic regions highlighted herein establish a robust candidate framework. Future research should leverage larger validation populations and transition toward multi-omics architectures—such as the combined transcriptome and proteome analyses recently applied to ovine wool and fleece traits [49]—to fully decode the functional landscape of these regulatory elements, thereby securing the sustainable utilization and precision conservation of this elite plateau genetic resource.

## Figures and Tables

**Figure 1:**
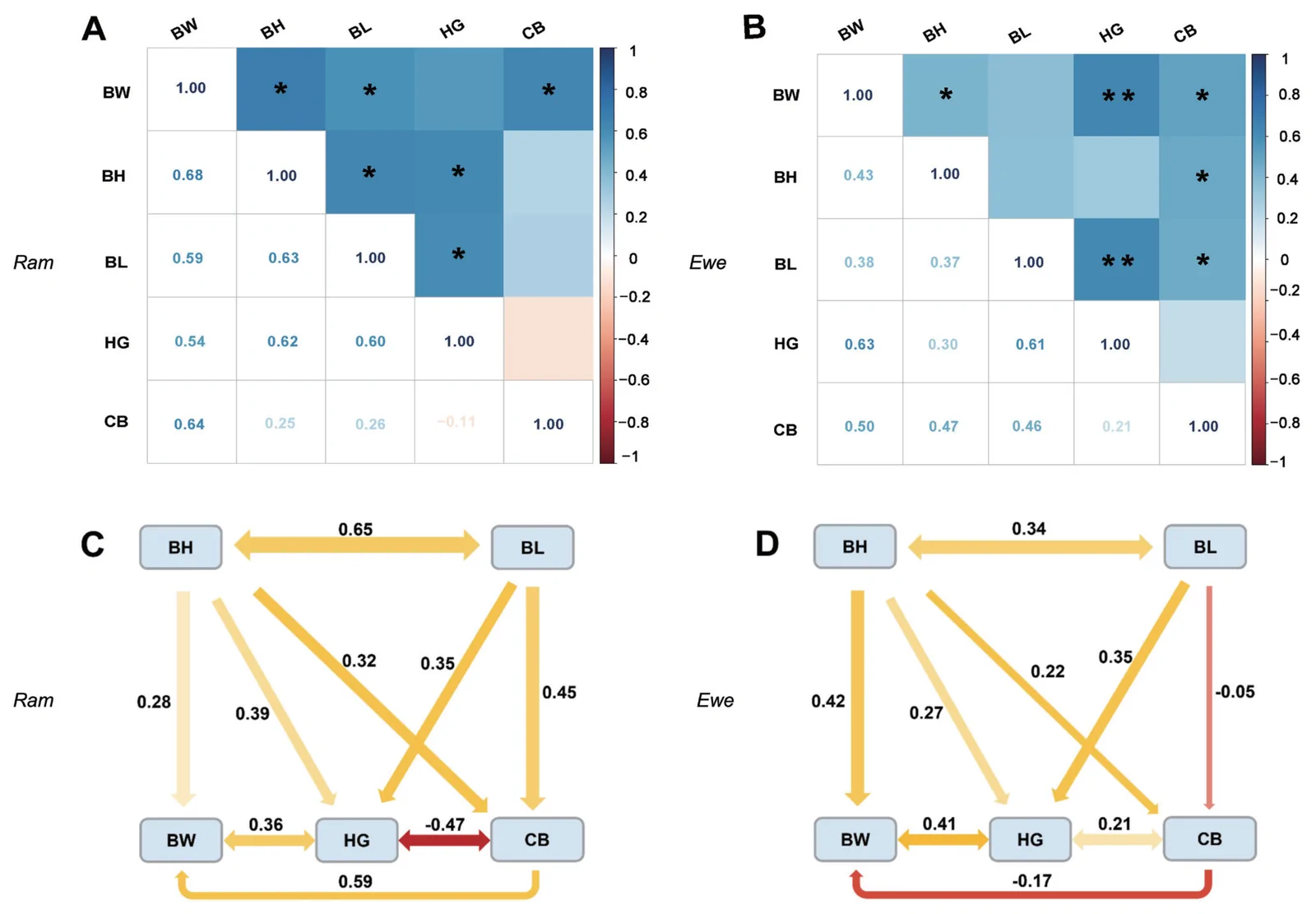
Correlation and path analysis results of growth traits in Gamba sheep. (A, B) Correlation heatmaps of body conformation traits in rams (A) and ewes (B). The color scale represents the Pearson correlation coefficient (r), and asterisks indicate statistical significance (* p<0.05, ** p<0.01). (C, D) Path analysis diagram of body conformation traits in rams (C) and ewes (D). BW, body weight; BH, body height; BL, body length; HG, heart girth; CB, cannon bone circumference.

**Figure 2:**
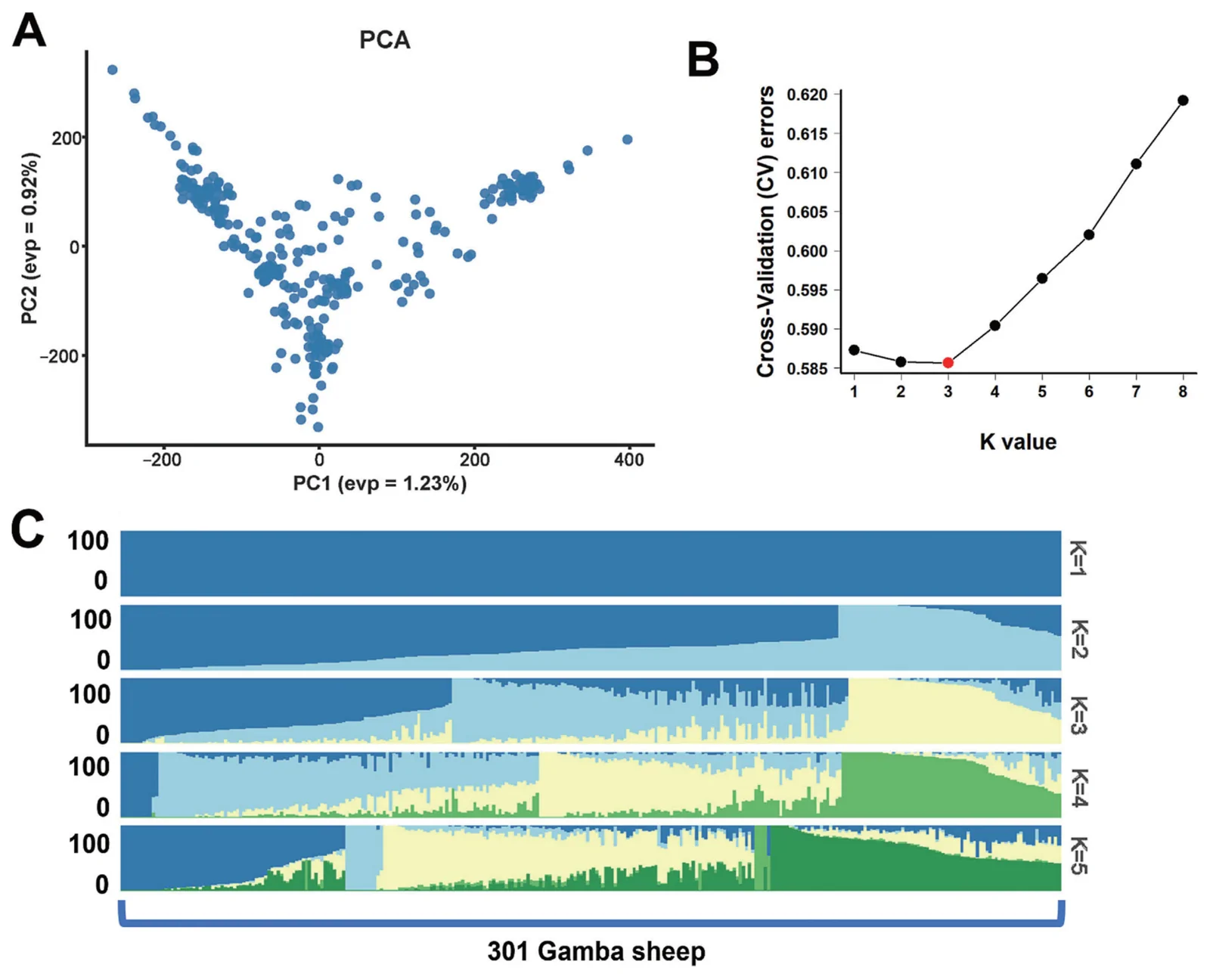
Genetic background of the 301 Gamba sheep population. (A) Principal component analysis (PCA) scores plot based on whole-genome SNP data. The first two components, PC1 and PC2, explain 1.23% and 0.92% of the total genetic variance, respectively, reflecting the high genetic homogeneity of the population. (B) Cross-validation error scores for different numbers of ancestral clusters (*K* = 1–8). The error reaches its minimum at *K* = 3, identifying the optimal number of clusters. (C) Ancestry composition inferred by *ADMIXTURE* analysis for *K* = 2 to 5. Each vertical bar represents an individual, with colors indicating the proportion of inherited ancestral components.

**Figure 3:**
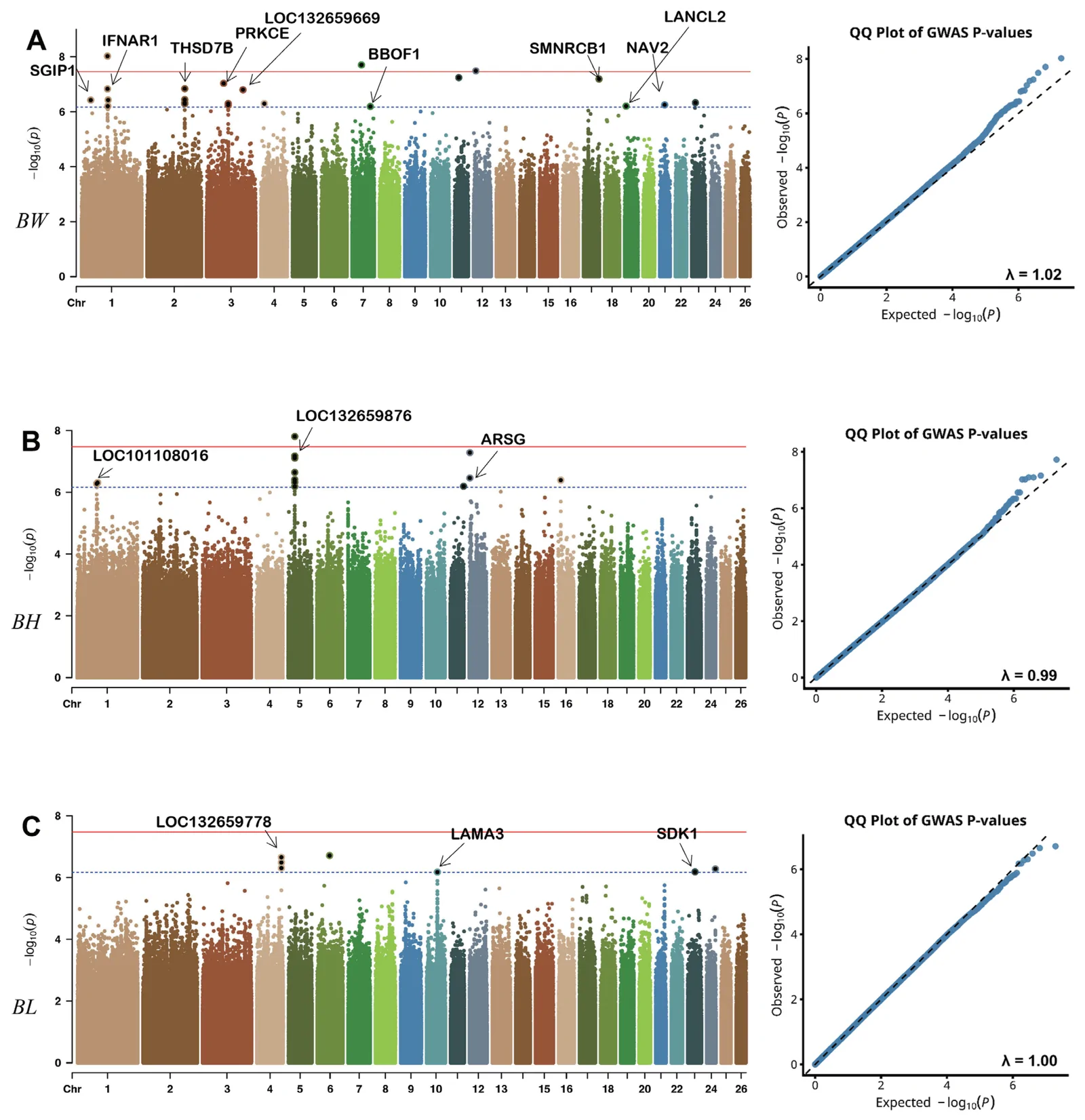
Manhattan and Q-Q plots of GWAS results for production and body conformation traits in Gamba sheep. (A) Results for body weight (BW), a primary production trait. (B) Results for body height (BH) and (C) body length (BL), representing body conformation traits. The red solid and blue dashed horizontal lines in the Manhattan plots represent the strict genome-wide significance threshold (p = 3.42×10^−^^8^) and the suggestive significance threshold (p = 6.84×10^−^^7^), respectively. Annotated gene symbols indicate significantly associated loci. The corresponding Q-Q plots demonstrate effective control of population stratification, with genomic inflation factors (λ) near 1.0 (e.g., *λ_BW_* = 1.02). Q-Q, quantile-quantile; GWAS, genome-wide association studies.

**Figure 4:**
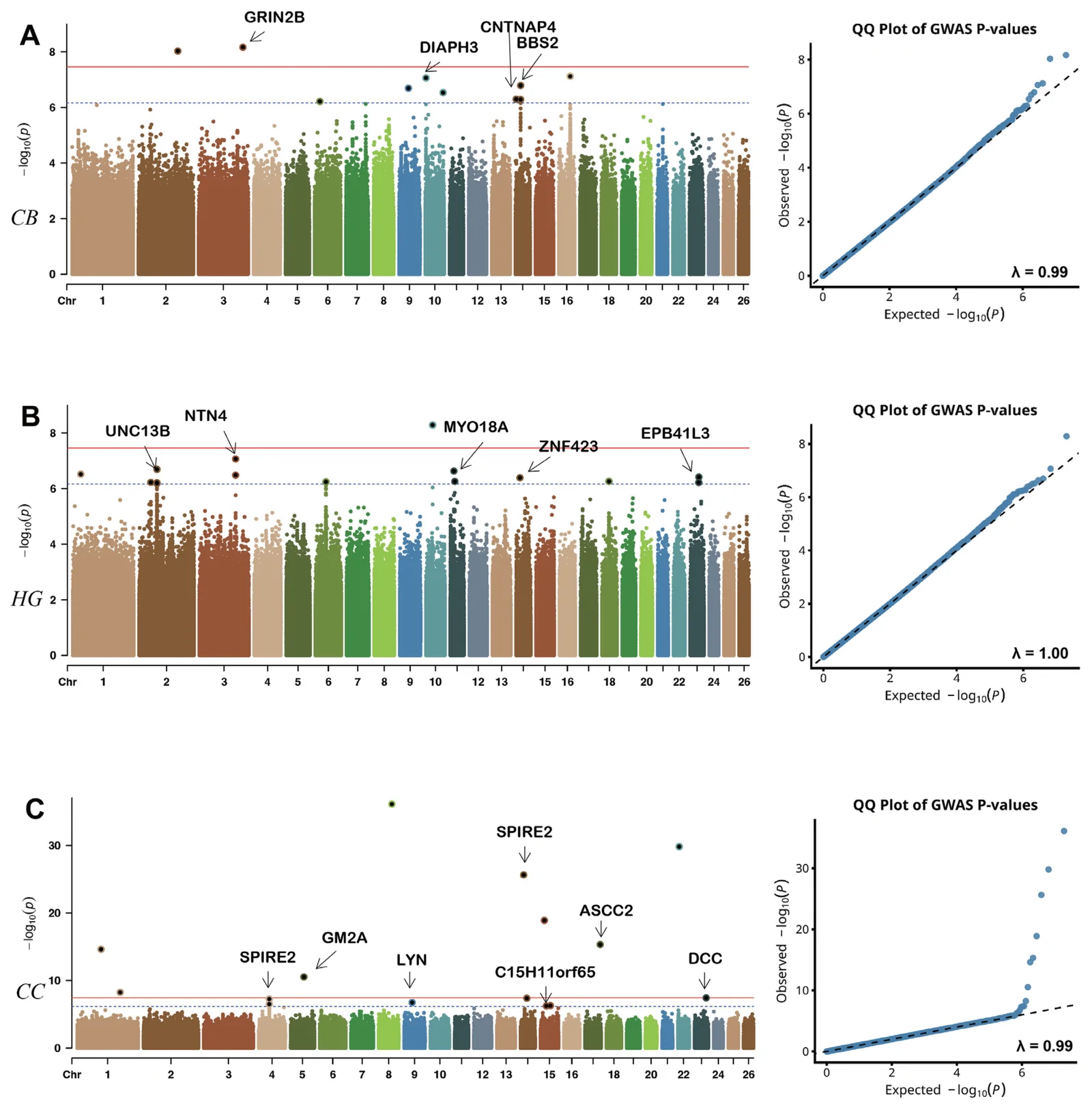
Manhattan and Q-Q plots of GWAS results for body conformation and coat color (CC) traits in Gamba sheep. (A) Results for cannon bone circumference (CB), (B) heart girth (HG), and (C) CC. The red solid and blue dashed horizontal lines in the Manhattan plots represent the strict genome-wide significance threshold (p = 3.42×10^−^^8^) and the suggestive significance threshold (p = 6.84×10^−^^7^), respectively. Annotated gene symbols indicate significantly associated loci. The corresponding Q-Q plots demonstrate effective control of population stratification, with genomic inflation factors (λ) near 1.0. Q-Q, quantile-quantile; GWAS, genome-wide association studies.

**Figure 5:**
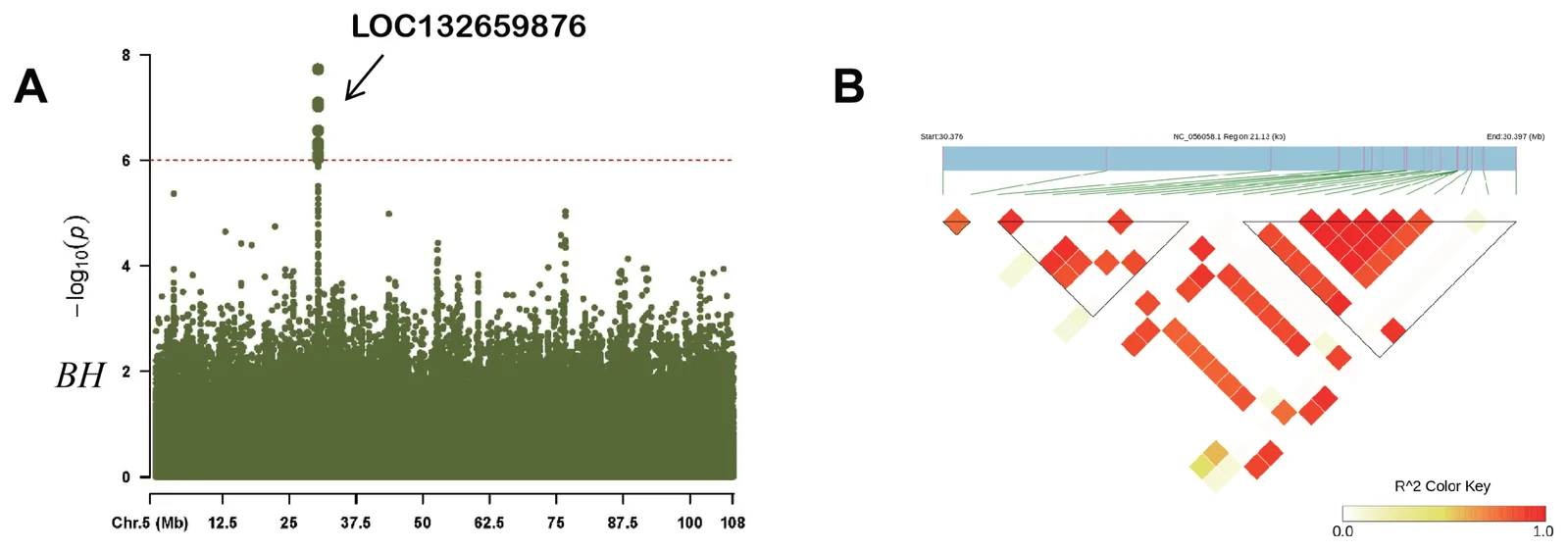
Local Manhattan plot and linkage disequilibrium (LD) heatmap for the candidate region on OAR5. (A) Magnified Manhattan plot of the significant QTL on chromosome 5 (30.37–30.39 Mb) associated with body height (BH). (B) Haplotype block analysis for the lead SNP within the peak region on OAR5. The color intensity in the heatmap represents the strength of LD (r^2^), with red indicating strong local linkage (r^2^≈1.0).

**Figure 6:**
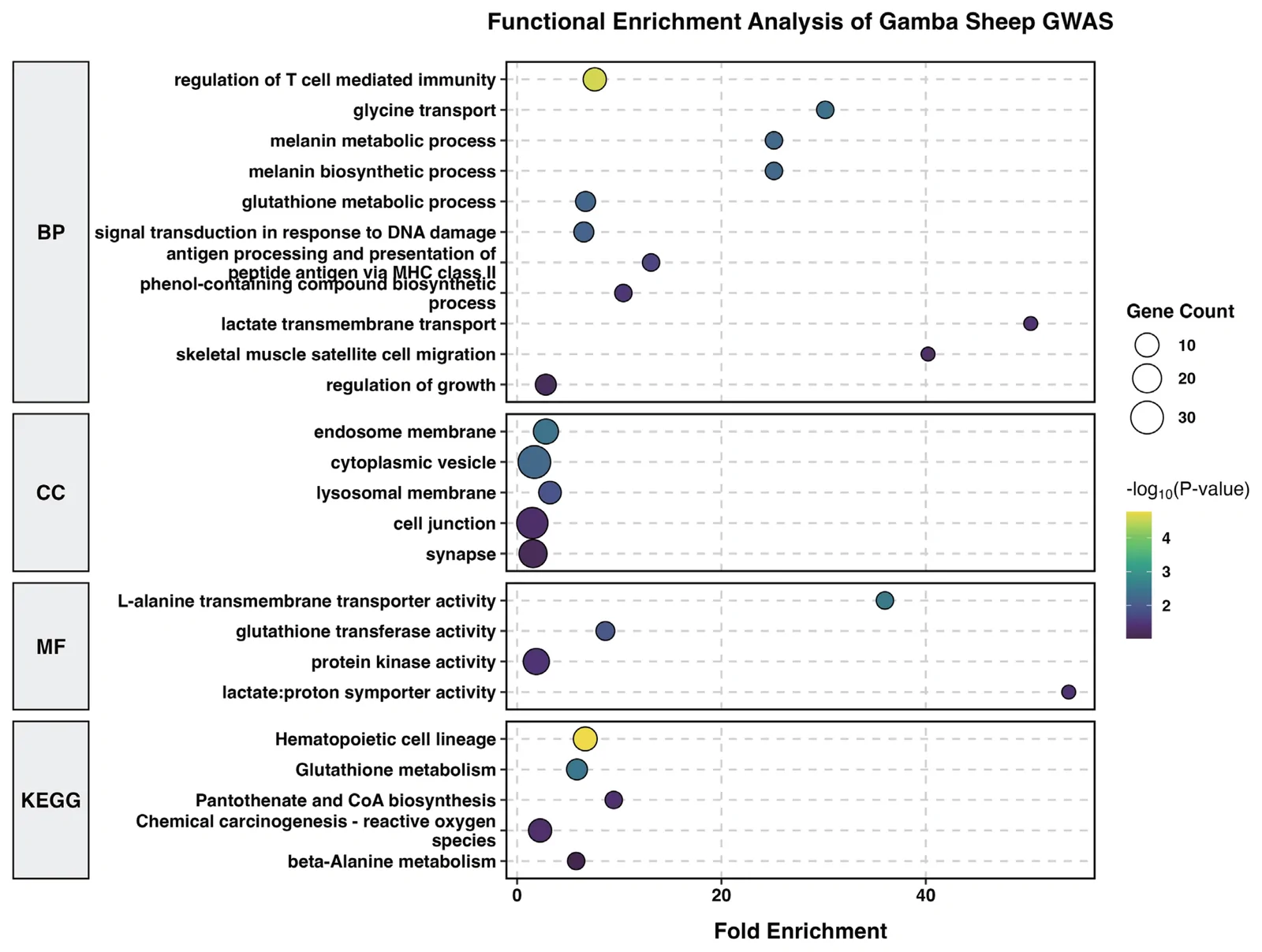
Gene Ontology (GO) and Kyoto Encyclopedia of Genes and Genomes (KEGG) pathway enrichment analyses of candidate genes. GWAS, genome-wide association studies; BP, biological process; CC, cellular component; MF, molecular function.

**Table 1:** Descriptive statistics of body weight and body measurement traits in Gamba sheep

Trait	Mean	Max	Min	SE	CV (%)
BW (kg)	21.04	46.30	8.80	0.35	29.89
BH (cm)	61.28	75.60	44.00	0.36	10.56
BL (cm)	59.87	79.60	40.00	0.41	12.24
HG (cm)	80.43	118.00	52.30	0.71	15.85
CB (cm)	8.29	10.00	6.00	0.07	15.37

SE, standard error; CV, coefficient of variation; BW, body weight; BH, body height; BL, body length; HG, heart girth; CB, cannon bone circumference.

**Table 2:** Summary of significant SNPs associated with coat color (CC), body weight (BW), and body conformation traits in Gamba sheep

Trait	Chromosome	SNP location	MAF	p-value	Genotype	Functional annotation	Candidate gene	Effect (beta)	SE	PVE (%)
Body height	1	94162992	0.01	5.45e-07	G/A	Intron	*LOC101108016*	−7.35	1.42	4.53
	5	30380965	0.03	8.05e-08	T/A	Intron	*LOC132659876*	−5.17	0.93	6.17
	12	2988108	0.01	4.59e-07	C/A	Intron	*ARSG*	−7.37	1.41	5.20
Body length	4	113337349	0.03	3.28e-07	C/T	Intron	*LOC13265778*	−8.65	1.69	9.38
	23	33186421	0.03	6.59e-07	C/T	Intron	*LAMA3*	3.69	0.74	6.14
	24	40168178	0.12	5.29e-07	T/C	Intron	*SDK1*	6.71	1.34	5.65
Body weight	1	42362866	0.17	3.74e-07	C/G	Intron	*SGIP1*	−6.58	1.30	7.90
	1	122850988	0.04	3.69e-07	T/C	Intron	*IFNAR1*	−4.34	0.86	4.05
	2	173686636	0.16	1.44e-07	T/G	Intron	*THSD7B*	−9.19	1.75	8.42
	3	78538918	0.16	9.21e-08	T/G	Intron	*PRKCE*	−3.75	1.95	3.62
	4	15679843	0.02	5.03e-07	G/T	Intron	*LOC132659669*	5.94	1.18	4.16
	7	83479327	0.03	6.42e-07	T/C	Intron	*BBOF1*	5.20	1.05	3.92
	17	71299017	0.16	6.41e-08	A/G	Intron	*SMNRCB1*	−8.15	1.51	8.85
	19	723022	0.16	6.86e-07	T/C	Intron	*LANCL2*	−8.69	1.75	7.57
	21	22721446	0.16	5.59e-07	C/T	Intron	*NAV2*	−7.63	1.52	7.68
Heart girth	2	53184968	0.12	5.96e-07	C/T	Exon	*UNC13B*	5.28	1.06	5.08
	3	165613518	0.08	3.27e-07	C/T	Intron	*NTN4*	6.42	1.26	5.40
	11	20472388	0.13	5.50e-07	A/G	Intron	*MYO18A*	5.07	1.01	4.98
	14	17599114	0.02	4.05e-07	T/C	Intron	*ZNF423*	12.49	2.47	5.35
	23	38966213	0.02	6.02e-07	G/A	Intron	*EPB41L3*	−11.52	2.31	5.29
Cannon bone circumference	3	201902244	0.06	6.82e-09	G/A	Intron	*GRIN2B*	0.73	0.13	9.51
	10	2904040	0.02	8.66e-08	A/C	Intron	*DIAPH3*	1.20	0.22	8.84
	14	3128423	0.02	5.04e-07	T/A	Intron	*CNTNAP4*	0.97	0.19	6.81
	14	23613516	0.06	5.18e-07	T/C	Intron	*BBS2*	−0.61	0.12	6.60
Coat color	4	48475352	0.03	5.44e-08	C/T	Intron	LOC132659732	0.20	0.04	8.94
	5	60285729	0.20	2.93e-11	G/C	Exon	GM2A	−0.12	0.02	12.81
	9	36156148	0.03	1.78e-07	C/T	Intron	LYN	0.19	0.04	8.30
	14	14221535	0.36	2.31e-26	C/T	Intron	SPIRE2	0.25	0.02	27.27
	15	17702155	0.02	1.28e-19	G/A	Intron	C15H11orf65	0.41	0.05	21.43
	17	68937223	0.17	4.72e-16	C/T	Intron	ASCC2	0.16	0.02	17.96
	23	52706998	0.06	3.73e-08	G/A	Intron	DCC	−0.14	0.03	9.14

SNP, single nucleotide polymorphism; MAF, minor allele frequency; SE, standard error; PVE, phenotypic variance explained.

## Data Availability

The original 301 sheep whole genome sequencing data in FASTQ format have been deposited in the Genome Sequence Archive on the China National Center for Bioinformation (CNCB) platform, under accession number CRA024483 (https://ngdc.cncb.ac.cn/gsa/browse/CRA024483). The generated SNP variants file has been deposited in the Genome Variation Map with the accession number GVM001013 (accessible at https://ngdc.cncb.ac.cn/gvm/getProjectDetail?project=GVM001013). Additionally, standardized phenotypic measurement spreadsheets used in this study are available from the corresponding author upon reasonable request.

## SUPPLEMENTARY MATERIAL

**Supplement 1**. Phenotypic distribution of body height (BH) in Gamba sheep. **Supplement 2**. Phenotypic distribution of body length (BL) in Gamba sheep.

**Supplement 3**. Phenotypic distribution of heart girth (HG) in Gamba sheep. **Supplement 4**. Phenotypic distribution of cannon bone circumference (CB) in Gamba sheep.

**Supplement 5**. Phenotypic distribution of body weight (BW) in Gamba sheep.

**Supplement 6**. Chromosomal SNP density map of Gamba sheep.

**Supplement 7**. Population phylogenetic tree of 301 Gamba sheep individuals.

**Supplement 8**. Kinship heatmap of 301 Gamba sheep individuals.

**Supplement 9**. Comparison of GWAS models for body height (BH) in Gamba sheep.

**Supplement 10**. Comparison of GWAS models for body weight (BW) in Gamba sheep.

**Supplement 11**. Comparison of GWAS models for body length (BL) in Gamba sheep.

**Supplement 12**. Comparison of GWAS models for heart girth (HG) in Gamba sheep.

**Supplement 13**. Comparison of GWAS models for cannon bone circumference (CB) in Gamba sheep.

**Supplement 14**. Comparison of GWAS models for coat color (CC) in Gamba sheep.

**Supplement 15**. Phenotypic validation of low-MAF significant SNPs for growth traits in Gamba sheep.

**Supplement 16**. Statistical power analysis of the GWAS workflow.

**Supplement 17**. Genome-wide average linkage disequilibrium (LD) decay over physical distance in Gamba sheep.

**Supplement 18**. Summary of significant SNP loci and candidate genes associated with important economic traits in Gamba sheep based on GWAS.

**Supplement 19**. Gene Ontology (GO) enrichment analysis of candidate genes.

**Supplement 20**. KEGG pathway enrichment analysis of candidate genes.
