# Supplementary material for: Genome-wide association studies of body weight, body conformation traits and coat color in Gamba sheep
Source: Anim Biosci. 2026 Jun 15;39(10):260295. doi: 10.5713/ab.260295 (PMC13662935; doi:10.5713/ab.260295)
Supplement: Supplementary file 7 [file ab-260295-Supplementary-15.pdf]

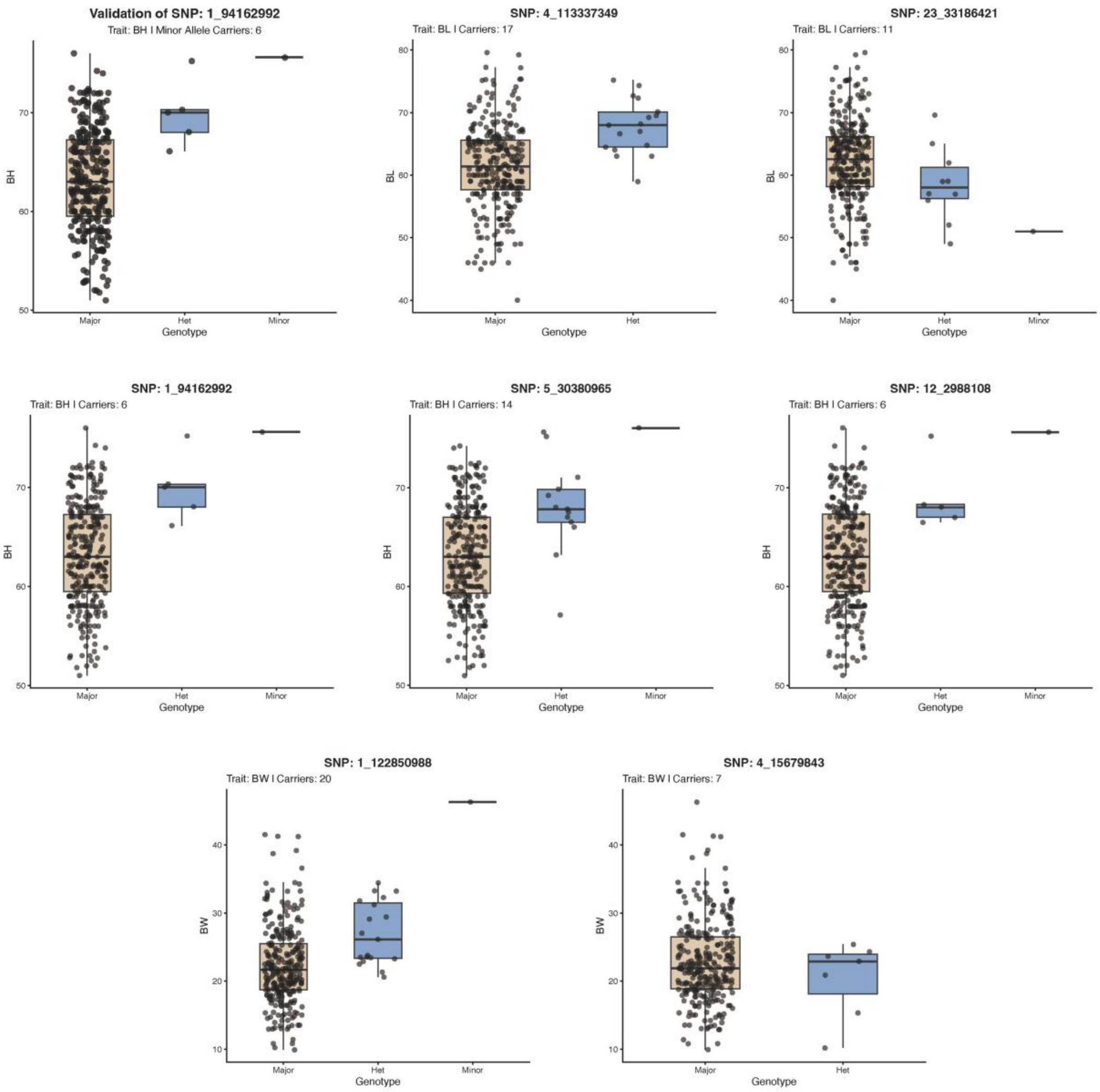


**Supplement 15. Phenotypic validation of low-MAF significant SNPs for growth traits in Gamba sheep.** Combined box-and-whisker and jitter plots illustrate the distribution of phenotypic values across different genotypes (Major, Het, and Minor) for representative single nucleotide polymorphisms (SNPs) associated with body height (BH), body length (BL), and body weight (BW). Each dot represents an individual animal within the validation population (N = 301). The title of each panel denotes the specific marker coordinate, and the subtitle indicates the corresponding trait along with the total number of minor allele carriers (Het + Minor). Horizontal lines within the boxes represent the median values, while the whiskers extend to 1.5 times the interquartile range.
