# Supplementary material for: Genome-wide association studies of body weight, body conformation traits and coat color in Gamba sheep
Source: Anim Biosci. 2026 Jun 15;39(10):260295. doi: 10.5713/ab.260295 (PMC13662935; doi:10.5713/ab.260295)
Supplement: Supplementary file 8 [file ab-260295-Supplementary-16.pdf]

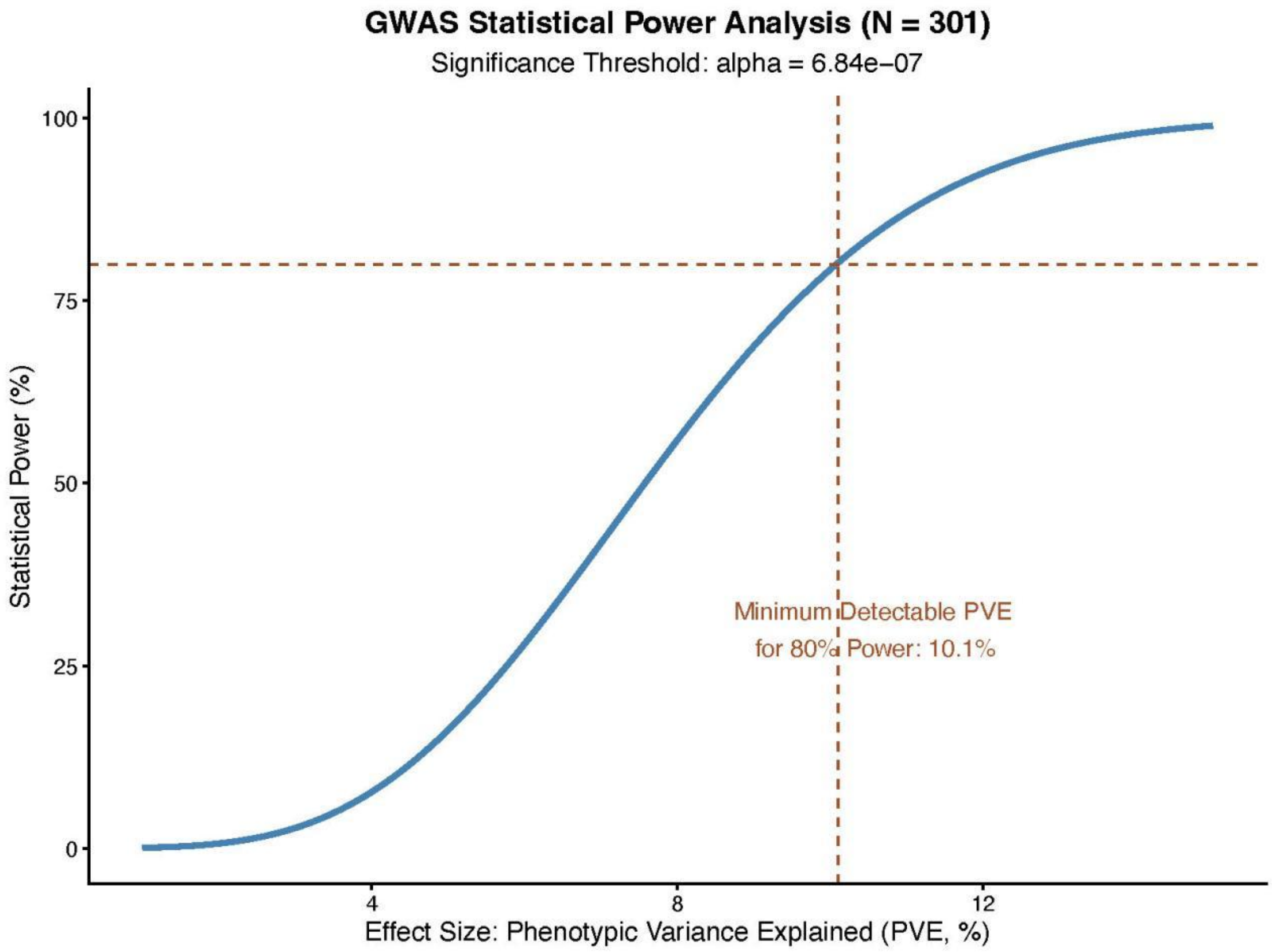

Supplement 16. Statistical power analysis of the GWAS workflow.

The S-shaped power curve was simulated based on a population size of 296 individuals (N = 296) and a genome-wide significance threshold of $\lambda = 6.84 \times 10^{-7}$. The x-axis represents the effect size, quantified as the percentage of Phenotypic Variance Explained (PVE, %). The y-axis denotes the estimated statistical power (%). The dashed horizontal and vertical reference lines indicate that the experimental design achieves a standard 80% statistical power to detect causal variants with a minimum detectable PVE of 10.1%.
