# Supplementary material for: Genome-wide association studies of body weight, body conformation traits and coat color in Gamba sheep
Source: Anim Biosci. 2026 Jun 15;39(10):260295. doi: 10.5713/ab.260295 (PMC13662935; doi:10.5713/ab.260295)
Supplement: Supplementary file 9 [file ab-260295-Supplementary-17.pdf]

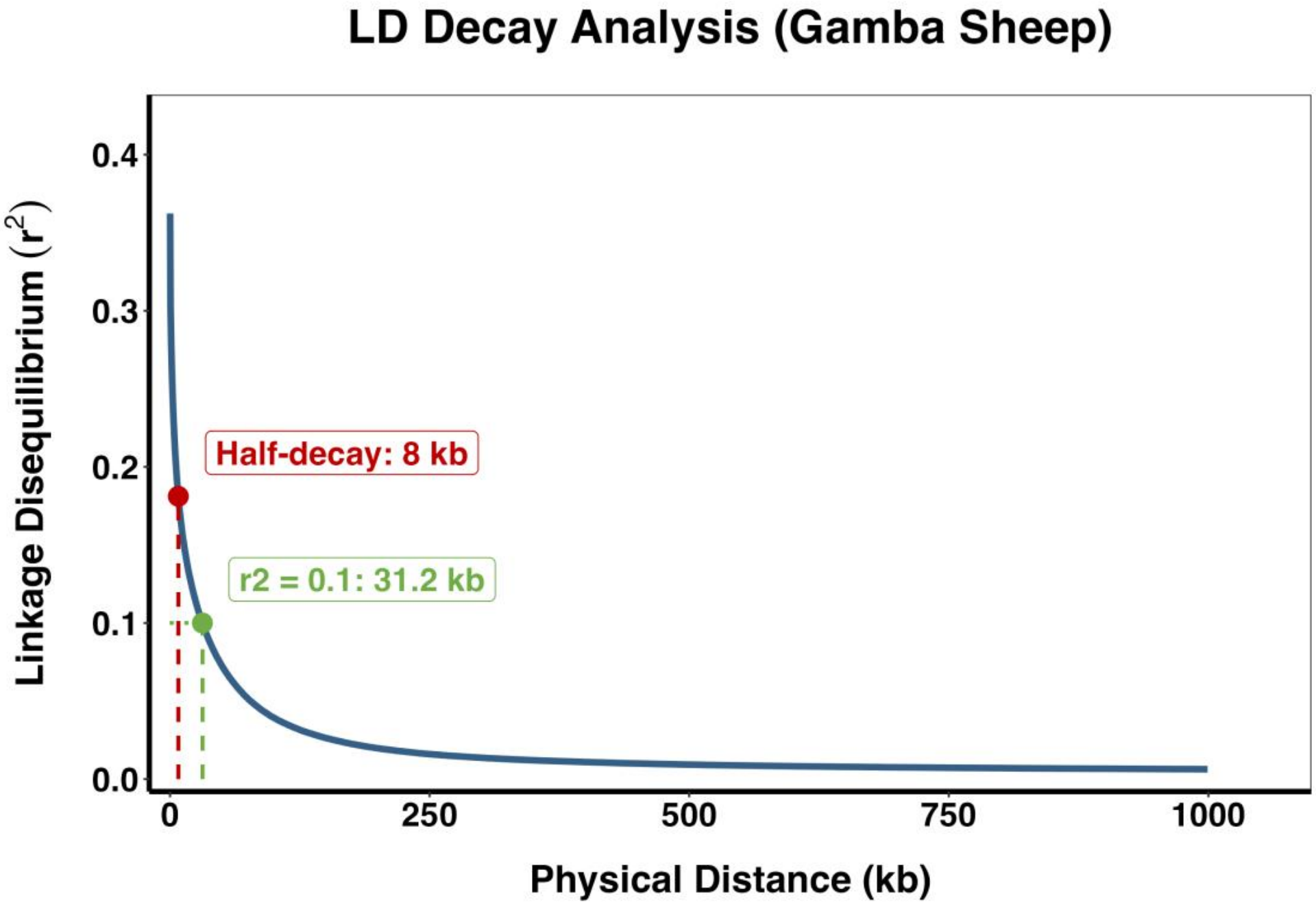

Supplement 17. Genome-wide average linkage disequilibrium (LD) decay over physical distance in Gamba sheep. The decay curve illustrates the rapid decrease in the average linkage disequilibrium coefficient (r^2, y-axis) as the physical distance between genomic markers increases (kb, x-axis). The red dashed line highlights the half-decay distance, where the r^2 value drops to half of its maximum initial value at approximately 7.99 kb (labeled as 8 kb). The green dashed line indicates the physical distance (31.17 kb, labeled as 31.2 kb) at which the r^2 value falls below the background threshold of 0.1, signifying that global LD has nearly dissipated beyond this genomic interval.

Supplement 18. Summary of significant SNP loci and candidate genes associated with important economic traits in Gamba sheep based on GWAS.

Trait	Chromosome	Start Position	End Position	Gene Symbol
BW	1	120023245	120112682	ADCY10
	1	121733042	121734018	C1H21orf140
	1	119638494	119723234	CD247
	1	121386868	121446523	CLIC6
