# Supplementary material for: Genome-wide association studies of body weight, body conformation traits and coat color in Gamba sheep
Source: Anim Biosci. 2026 Jun 15;39(10):260295. doi: 10.5713/ab.260295 (PMC13662935; doi:10.5713/ab.260295)
Supplement: Supplementary file 10 [file ab-260295-Supplementary-18.pdf]

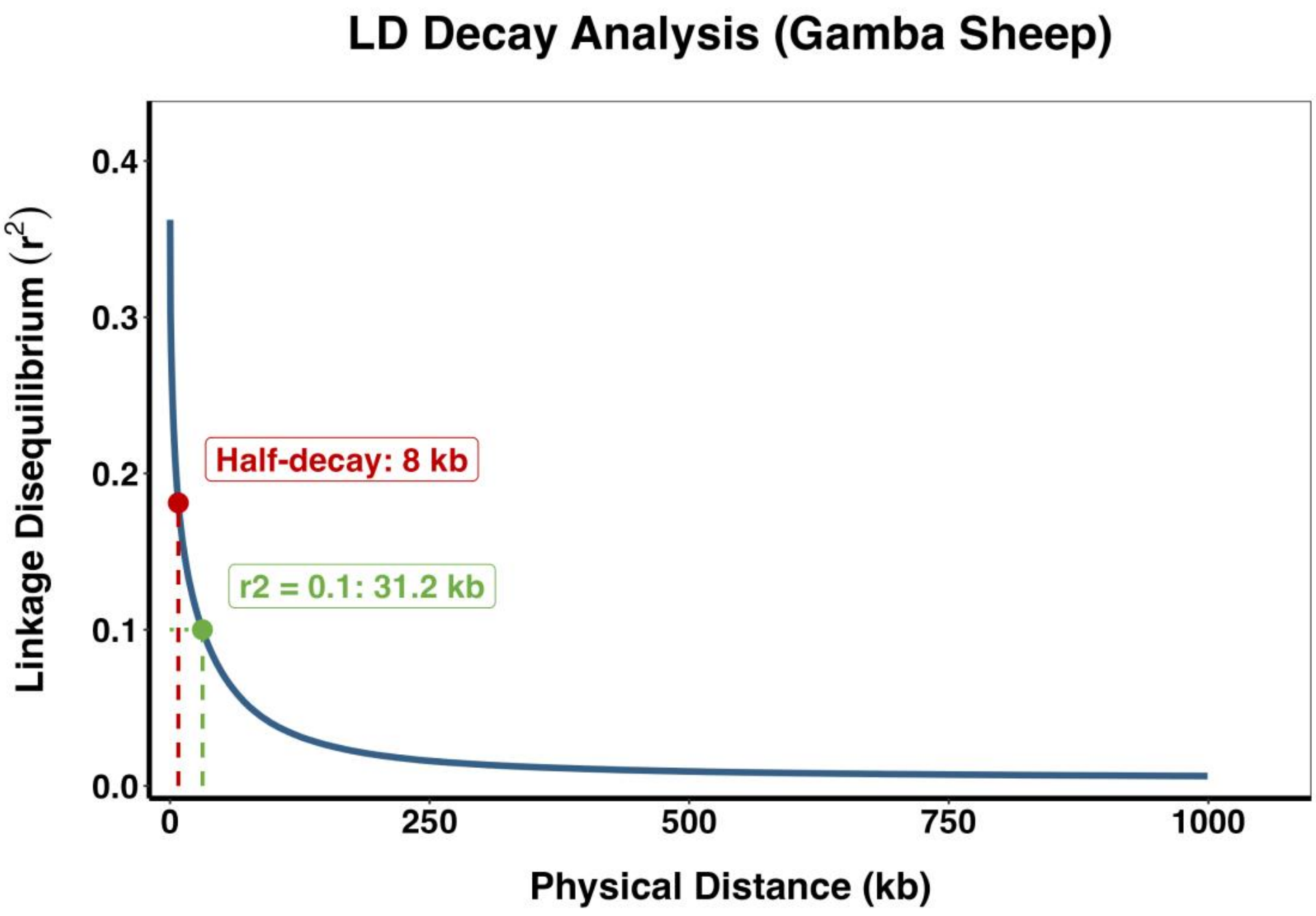

Supplement 17. Genome-wide average linkage disequilibrium (LD) decay over physical distance in Gamba sheep. The decay curve illustrates the rapid decrease in the average linkage disequilibrium coefficient (r^2, y-axis) as the physical distance between genomic markers increases (kb, x-axis). The red dashed line highlights the half-decay distance, where the r^2 value drops to half of its maximum initial value at approximately 7.99 kb (labeled as 8 kb). The green dashed line indicates the physical distance (31.17 kb, labeled as 31.2 kb) at which the r^2 value falls below the background threshold of 0.1, signifying that global LD has nearly dissipated beyond this genomic interval.

Supplement 18. Summary of significant SNP loci and candidate genes associated with important economic traits in Gamba sheep based on GWAS.

Trait	Chromosome	Start Position	End Position	Gene Symbol
BW	1	120023245	120112682	ADCY10
	1	121733042	121734018	C1H21orf140
	1	119638494	119723234	CD247
	1	121386868	121446523	CLIC6

1	119743422	119758072	CREG1
1	42495244	42601940	DNAI4
1	122676212	122679866	DNAJC28
1	42426121	42455138	DYNLT5
1	122633710	122663360	GART
1	122846951	122876101	IFNAR1
1	122979204	123014718	IFNAR2
1	122946973	122977687	IL10RB
1	42475766	42479267	INSL5
1	121756268	121767085	KCNE2
1	121286717	121301943	LOC101111769
1	42219846	42229172	LOC101112774
1	121530885	121531431	LOC101118835
1	119751983	119754596	LOC105612995
1	121153898	121154016	LOC114110862
1	121324987	121325095	LOC114111158
1	42505229	42505348	LOC114111274
1	122747126	122747250	LOC114111302
1	121149522	121149640	LOC114111399
1	42179160	42181064	LOC114113027
1	119905421	119905576	LOC114113255
1	122875442	122877556	LOC114117969
1	123024471	123028722	LOC121816116
1	121169209	121172855	LOC121818669

1	122977779	122992256	LOC132658299
1	122789677	122800260	LOC132659857
1	119910477	119998030	MPZL1
1	122029349	122095890	MRPS6
1	119428447	119634966	POU2F1
1	121508809	121632745	RCAN1
1	119825175	119896669	RCSD1
1	42187118	42424690	SGIP1
1	122061710	122087035	SLC5A3
1	121721588	121751006	SMIM11
1	121705009	121711208	SMIM34
1	122694829	122737339	TMEM50B
1	4044786	4044857	TRNAC-ACA
1	119904491	119904562	TRNAP-AGG
1	119903767	119903838	TRNAP-CGG
1	122756234	122789510	IFNGR2
2	173013644	174062311	THSD7B
1	28122341	28122413	TRNAW-CCA
3	19470051	19587764	HPCAL1
3	99547239	99641780	IL1R1
3	99742003	99778253	IL1R2
3	99453643	99482002	IL1RL2
3	19601907	19602039	LOC114114242
3	99633955	99634031	LOC114114266
3	99682469	99684246	LOC132659418
3	168211020	168252873	LOC132659462
3	19304961	19308147	LOC132659563
3	99837227	99988521	MAP4K4

3	19596255	19603526	ODC1
3	78150489	78695898	PRKCE
3	19308282	19315309	RRM2
3	78738454	78966372	SRBD1
1	4828683	4828755	TRNAG-CCC
4	15868108	16020359	ASNS
4	15670584	15771750	LOC132659669
4	15770246	15778970	TAC1
7	83503937	83524401	ALDH6A1
7	83474392	83509495	BBOF1
7	83425603	83439234	COQ6
7	83439355	83474284	ENTPD5
7	83409230	83425307	FAM161B
7	42542167	42809344	FRMD6
7	83524876	83641748	LIN52
7	42239820	42296640	LOC105615691
7	83334718	83343318	LOC114115645
7	42236085	42236865	LOC114115720
7	42466465	42466568	LOC114115986
7	42214784	42216412	LOC121819966
7	83216971	83285422	MIDEAS
7	83343374	83364371	PTGR2
7	42296809	42310565	TMX1
7	83676440	83696983	VSX2
7	83366393	83409216	ZNF410
9	73176392	73217051	DCSTAMP
9	73072431	73171953	DPYS
9	73284981	73886589	RIMS2

11	18831308	18991288	LOC101117683
11	18671368	18700272	LOC121820559
11	18820577	18827534	LOC121820560
11	18651624	18657266	LOC132657313
11	18365735	18619391	NF1
11	18731059	18745606	WSB1
17	71266683	71272774	C17H22orf15
17	71388541	71464052	CABIN1
17	71272746	71275204	CHCHD10
17	71306784	71308870	DERL3
17	71491517	71500580	GGT5
17	71359204	71365537	LOC101111397
17	71367351	71374939	LOC101111664
17	71383171	71385840	LOC101111911
17	71377071	71382199	LOC101118990
17	71246840	71253598	LOC105602956
17	71337851	71340354	LOC105602957
17	71193086	71213638	LOC105602976
17	70750757	71220068	LOC114108841
17	71107573	71108251	LOC114118884
17	71162712	71163320	LOC114118889
17	71123580	71124301	LOC121816875
17	71115512	71116181	LOC121816877

	17	71113786	71120097	LOC121816885
	17	71121855	71124213	LOC121816886
	17	71221384	71235046	LOC121816984
	17	71316419	71328503	LOC121816985
	17	71129911	71136219	LOC132658059
	17	71340535	71341210	MIF
	17	71278262	71287252	MMP11
	17	71314815	71331331	SLC2A11
	17	71288769	71305020	SMARCB1
	17	71474397	71482046	SUSD2
	1	22218050	22218122	TRNAS-GGA
	17	71267588	71268826	VPREB3
	17	71254927	71266653	ZNF70
	19	880958	1095437	EGFR
	19	5258457	5449678	GADL1
	19	685998	769387	LANCL2
	19	531525	531987	LOC106990554
	1	63295425	63295497	TRNAV-CAC
	19	496514	664321	VOPP1
	21	22881691	22881823	LOC114110213
	21	22345557	23145527	NAV2
	23	16366025	16367346	LOC101106129
	23	14139462	14139577	LOC114110495
	23	14129534	14132725	LOC132658466
	23	14132836	14293753	PIK3C3
HG	1	36292217	36324749	LOC105612174

1	35934288	36096339	LOC114115115
1	35987757	36004604	LOC114115126
1	36318427	36721364	NFIA
2	53362036	53373760	ATOSB
2	53005826	53130571	LOC101109422
2	53064695	53065214	LOC101117475
2	80859210	80860410	LOC101119696
2	53187296	53188638	LOC105607745
2	81267175	81315274	LURAP1L
2	53382956	53390668	PIGO
2	52930825	52994044	RUSC2
2	53374737	53377897	STOML2
2	81182552	81200943	TYRP1
2	53130799	53350246	UNC13B
3	165808407	165822869	AMDHD1
3	165772273	165807826	CCDC38
3	165828290	165850520	HAL
3	165744858	165756067	LOC132659458
3	165858005	165890879	LTA4H
3	165423639	165434046	NEUROD4
3	165585734	165716151	NTN4
3	165765813	165773251	SNRPF
3	165470158	165504630	TESPA1
6	48380476	48381859	LOC101107099
10	30408378	30436392	ALOX5AP
10	29934343	30046732	B3GLCT
10	30503002	30633115	HMGB1
10	30086904	30110240	HSPH1

10	30132318	30170501	LOC105616258
10	30321197	30340375	LOC132657250
10	30073461	30087868	LOC132657253
10	30292368	30308359	MEDAG
10	30260101	30286188	TEX26
1	98036334	98036407	TRNAN-GUU
1	28122341	28122413	TRNAW-CCA
10	30475351	30502897	USPL1
11	20530801	20537836	CRYBA1
11	20441822	20449310	LOC114116895
11	20576594	20576702	LOC114117036
11	20486807	20486938	LOC114117048
11	20378907	20480140	MYO18A
11	20538454	20565904	NUFIP2
11	20350930	20364542	PIPOX
11	20265514	20313571	SEZ6
11	20643810	20751150	TAOK1
1	104115251	104115322	TRNAM-CAU
14	17665440	17667230	LOC132657672
1	4828683	4828755	TRNAG-CCC
14	17495690	17838589	ZNF423
18	31193638	31203493	C18H15orf39
18	31135428	31140977	COMMD4
18	31428668	31439293	COX5A
18	31449221	31454932	FAM219B

	18	31168301	31169347	LOC105603152
	18	31177995	31182251	LOC105603153
	18	31203583	31211115	LOC121817103
	18	31176152	31179857	LOC121817104
	18	31260596	31303081	LOC132658083
	18	31107268	31118701	MAN2C1
	18	31455376	31464169	MPI
	18	31119318	31126195	NEIL1
	18	31329602	31354451	PPCDC
	18	31414154	31415592	RPP25
	18	31355066	31377158	SCAMP5
	18	31039254	31105240	SIN3A
	23	38055805	38847454	DLGAP1
	23	39340880	39568342	EPB41L3
	23	39478820	39482081	LOC105604471
	23	39105919	39106469	LOC121817745
	23	39273327	39280799	ZBTB14
CB	1	112632053	112640143	ADAMTS4
	1	112659164	112660421	APOA2
	1	112515224	112529664	ARHGAP30
	1	112616664	112622849	B4GALT3
	1	112765480	112793668	CFAP126
	1	112573863	112584675	DEDD
	1	112653106	112656316	FCER1G
	1	112556843	112569989	KLHDC9

1	112684265	112691111	LOC101105373
1	112746487	112756177	LOC114110816
1	112546620	112550972	LOC121820706
1	112517453	112520746	LOC121820707
1	112724768	112729926	MPZ
1	112643586	112652541	NDUFS2
1	112530444	112546704	NECTIN4
1	112570809	112573978	NIT1
1	112659164	112671032	NR1I3
1	112558903	112570751	PFDN2
1	112612716	112616571	PPOX
1	112733322	112763726	SDHC
1	112662451	112666898	TOMM40L
1	13044539	13044610	TRNAC-GCA
1	112839753	112839824	TRNAD-GUC
1	64970488	64970560	TRNAE-CUC
1	40611315	40611388	TRNAG-GCC
1	14052201	14052273	TRNAG-UCC
1	112840388	112840470	TRNAL-CAG
1	63295425	63295497	TRNAV-CAC
1	112601506	112605181	UFC1
1	112507825	112513683	USF1
1	112605307	112612017	USP21
1	10702984	10703056	TRNAE-UUC
2	179336493	179336599	LOC114113286

2	179230657	179237752	LOC132659223
3	201476037	202004971	GRIN2B
1	28122341	28122413	TRNAW-CCA
6	23761397	24085851	PPP3CA
7	89961364	90416423	CEP128
1	22218050	22218122	TRNAS-GGA
9	42258956	42432700	LOC101119648
9	42432440	42437361	LOC105609787
10	2489906	3061493	DIAPH3
10	2774863	2775176	LOC114116590
10	2974817	2975147	LOC114116682
10	2926518	2926682	LOC114116770
14	23707152	23751745	AMFR
14	23536584	23646280	BBS2
14	2897365	3185377	CNTNAP4
14	23754035	23935718	GNAO1
14	23441533	23469387	LOC101116336
14	23477648	23507467	LOC101116756
14	23514101	23515907	LOC101117184
14	23524839	23526197	LOC101117691
14	23533058	23534018	LOC101117955
14	23679351	23683262	LOC101118978
14	3317114	3404242	LOC114117781
14	23492069	23510186	LOC121816484

	14	23519733	23521742	MT1B
	14	23542234	23543477	MT3
	14	23571783	23574369	MT4
	14	23685617	23702412	NUDT21
	14	23650647	23685395	OGFOD1
	14	23380827	23422703	SLC6A2
	16	50529795	51722443	CDH12
	21	26524189	26528257	ACRV1
	21	26498334	26517408	CHEK1
	21	26445863	26462592	EI24
	21	26357363	26399323	FEZ1
	21	26455390	26456015	LOC101110715
	21	26050912	26345389	PKNOX2
	21	26470636	26493233	STT3A
BH	1	90021673	90064274	CAPZA1
	1	94156746	94171365	CD2
	1	93942487	94075034	IGSF3
	1	90203404	90242281	LOC101108016
	1	94195458	94199827	LOC121820073
	1	94106383	94117300	LOC132658115
	1	90217842	90235239	LOC132659453
	1	90217842	90235239	LOC132659453
	1	90069477	90096616	MOV10
	1	90097001	90111702	PPM1J
	1	94311362	94395045	PTGFRN
	1	90097001	90103059	RHOC
	1	90319385	90353156	SLC16A1
	1	90114939	90124558	TAFA3
	1	90166726	90166798	TRNAL-UAA

1	94171596	94172179	LOC121820072
5	30514414	30516487	LOC101113234
11	61062567	61082712	AMZ2
11	60958196	61060024	ARSG
11	60836198	60879725	FAM20A
11	61099586	61132884	GNA13
11	61082914	61088939	LOC105610545
11	60908821	60929600	LOC105610547
11	61150225	61155943	LOC114116809
11	60899045	60899680	LOC132657446
11	60879803	60900176	PRKAR1A
11	61147448	61221042	RGS9
11	61032389	61052826	SLC16A6
11	60930096	60958193	WIPI1
11	60899963	60906567	LOC121820674
11	60991955	60995477	LOC121820675
11	61076776	61078203	LOC121820676
11	61180116	61185572	LOC132657381
11	61239337	61247262	LOC132657382
11	60869135	60870294	LOC132657443
11	60967689	60971864	LOC132657444
12	3047416	3067280	BLACAT1
12	3113237	3141521	CDK18
12	2791699	2844593	DSTYK

	12	2962640	2969422	KLHDC8A
	12	3031505	3035546	LEMD1
	12	2954093	2954454	LOC114117284
	12	2954093	2954454	LOC114117284
	12	2970710	2985912	LOC132657491
	12	2970710	2985912	LOC132657491
	12	2923391	2942888	NUAK2
	12	2866366	2904128	TMCC2
	12	2797272	2798973	LOC114117336
	16	11174495	11444152	LOC106990170
BL	4	113065731	113155405	CUL1
	4	113160647	113217372	EZH2
	4	113423200	113423836	LOC121819467
	4	113494629	113501070	LOC121819468
	4	113335914	113340245	LOC132659778
	4	113340347	113357926	PDIA4
	1	13044539	13044610	TRNAC-GCA
	4	113477077	113493433	ZNF212
	4	113444191	113469855	ZNF282
	4	113409671	113437284	ZNF398
	4	113501339	113517208	ZNF783
	4	113377688	113396086	ZNF786
	6	57070747	57070994	LOC114115409
	10	49104321	49625788	KLF12
	23	33340147	33393740	ANKRD29
	23	33066000	33319383	LAMA3
	23	33287277	33287644	LOC114110478

	23	33076282	33088489	LOC132658467
	1	14052201	14052273	TRNAG-UCC
	23	32922012	33025180	TTC39C
	24	40244034	40275382	LOC132658541
	24	39857074	40586849	SDK1
CC	5	60138924	60187576	ANXA6
	17	68916271	68952486	ASCC2
	15	17518450	17674475	ATM
	4	48207853	48468067	ATXN7L1
	15	17691155	17772364	C15H11orf65
	17	68864027	68875820	CABP7
	5	60218505	60266709	CCDC69
	1	108958383	108962097	CD1
	14	28950183	29345571	CDH8
	4	48541902	48612644	CDHR3
	14	14104564	14122480	CDK10
	14	14285678	14287309	CENPBD1
	14	14097631	14104492	CHMP1A
	14	14049129	14061270	CPNE7
	14	14309691	14318138	DBNDD1
	23	52076711	53357354	DCC
	14	14268673	14284941	DEF8
	14	14083253	14089202	DPEP1
	15	17880387	17975679	EXPH5
	14	14152238	14191062	FANCA
	5	60485610	60570332	FAT2
	1	195878957	196119227	FGF12
	14	14321413	14356243	GAS8
	5	60280193	60297306	GM2A
	15	17465426	17518204	LOC101104855
	15	45450751	45451737	LOC101107549

17	68898338	68900398	LOC101108778
14	14336835	14339671	LOC101109035
1	109014353	109017860	LOC101110634
15	45366853	45367767	LOC101112510
14	14287955	14312814	LOC101113264
15	45431279	45432265	LOC101113268
15	45453069	45454016	LOC101113526
1	108850968	108854620	LOC101114089
1	108635784	108647456	LOC101114924
8	60206830	60207753	LOC101120251
15	26720695	26740054	LOC105602189
4	48544804	48545824	LOC105609617
14	28910867	28911553	LOC105610704
1	108690449	108693097	LOC105612800
1	108773383	108776133	LOC105614484
17	68958362	68958468	LOC114108930
4	48390790	48390925	LOC114114651
8	59926018	59926287	LOC114116200
9	36267725	36267791	LOC114116498
9	36282683	36282847	LOC114116499
14	14038953	14039037	LOC114118112
15	45307517	45308370	LOC114118261
15	17769995	17771103	LOC114118344
15	26629077	26629163	LOC114118631
1	108656653	108661308	LOC121816032
14	14062296	14068450	LOC121816363
14	14109650	14119443	LOC121816364
4	48559679	48600908	LOC121819397

8	59836754	59906231	LOC121820187
14	14101264	14104017	LOC132657730
15	26829894	26831528	LOC132657894
1	108835626	108848722	LOC132658249
4	48468260	48476793	LOC132659732
5	60480742	60495482	LOC132659833
8	59829086	59910672	LOC132660206
1	108985189	108992221	LOC443423
1	108911019	108914674	LOC443441
1	108708112	108793439	LOC443442
9	36127162	36236442	LYN
14	14251730	14252683	MC1R
9	36288318	36289509	MOS
17	68978228	69107905	MTMR3
17	68772724	68843739	NF2
17	68744714	68772472	NIPSNAP1
15	45327429	45369798	NLRP14
9	36313599	36364939	PLAG1
15	17835882	17874814	POGLUT3
15	45312713	45314272	RBMXL2
14	14038268	14041306	RPL13
9	36266837	36268318	RPS20
1	196199547	196200273	SELENOK
5	60420957	60587635	SLC36A1
5	60344889	60420792	SLC36A2
5	60306574	60334796	SLC36A3
14	14122482	14126454	SPATA2L
14	14011461	14032489	SPG7
14	14191182	14225621	SPIRE2
4	48661784	48693654	SYPL1
15	44930165	45142112	SYT9
14	14227944	14248162	TCF25
9	36060717	36086153	TGS1
17	68715327	68743865	THOC5
9	36026414	36060642	TMEM68
5	60075064	60126054	TNIP1
1	4044786	4044857	TRNAC-ACA
1	13044539	13044610	TRNAC-GCA
1	64970488	64970560	TRNAE-CUC
1	4828683	4828755	TRNAG-CCC
1	14052201	14052273	TRNAG-UCC

14	14129620	14139603	VPS9D1
9	35632201	35968200	XKR4
17	68874779	68897987	ZMAT5
15	45376188	45399285	ZNF214
15	45402724	45425204	ZNF215
