# Supplementary material for: Genome-wide association studies of body weight, body conformation traits and coat color in Gamba sheep
Source: Anim Biosci. 2026 Jun 15;39(10):260295. doi: 10.5713/ab.260295 (PMC13662935; doi:10.5713/ab.260295)
Supplement: Supplementary file 11 [file ab-260295-Supplementary-19.pdf]

Supplement 19. Gene Ontology (GO) enrichment analysis of candidate genes.

Category	Term	P-Value	Benjamini	FDR	Fisher Exact	User Ids
BP	antigen processing and presentation of exogenous antigen	1.66E-10	0.000000374	0.000000367	5.14E-12	LOC105602976,CD1,LOC443423,FCER1G,LOC105614484,LOC114108841,LOC101110634,LOC101114089,LOC443441,LOC443442
BP	stress response to copper ion	1.89E-09	0.00000142	0.0000014	6.32E-12	LOC101117955,MT1B,MT4,LOC101117691,MT3,LOC101117184
BP	detoxification of copper ion	1.89E-09	0.00000142	0.0000014	6.32E-12	LOC101117955,MT1B,MT4,LOC101117691,MT3,LOC101117184
BP	cellular response to zinc ion	0.000000005	0.00000211	0.00000207	2.51E-11	LOC101117955,MT1B,MT4,LOC101117691,MT3,LOC101117184
BP	antigen processing and presentation, endogenous lipid antigen via MHC class Ib	6.55E-09	0.00000211	0.00000207	9.37E-11	CD1,LOC443423,LOC105614484,LOC101110634,LOC101114089,LOC443441,LOC443442
BP	antigen processing and presentation, exogenous lipid antigen via MHC class Ib	6.55E-09	0.00000211	0.00000207	9.37E-11	CD1,LOC443423,LOC105614484,LOC101110634,LOC101114089,LOC443441,LOC443442
BP	antigen processing and presentation of lipid antigen via MHC class Ib	6.55E-09	0.00000211	0.00000207	9.37E-11	CD1,LOC443423,LOC105614484,LOC101110634,LOC101114089,LOC443441,LOC443442
BP	cellular response to copper ion	1.12E-08	0.00000315	0.00000309	7.46E-11	LOC101117955,MT1B,MT4,LOC101117691,MT3,LO

						C101117184
BP	cellular response to cadmium ion	2.22E-08	0.000005	0.0000049	1.85E-10	LOC101117955,MT1B,MT4,LOC101117691,MT3,LOC101117184
BP	response to copper ion	2.22E-08	0.000005	0.0000049	1.85E-10	LOC101117955,MT1B,MT4,LOC101117691,MT3,LOC101117184
BP	response to cadmium ion	4.03E-08	0.00000826	0.0000081	4.04E-10	LOC101117955,MT1B,MT4,LOC101117691,MT3,LOC101117184
BP	stress response to metal ion	0.00000017	0.000032	0.0000314	2.56E-09	LOC101117955,MT1B,MT4,LOC101117691,MT3,LOC101117184
BP	detoxification of inorganic compound	0.000000253	0.000044	0.0000432	4.23E-09	LOC101117955,MT1B,MT4,LOC101117691,MT3,LOC101117184
BP	cellular response to metal ion	0.00000107	0.000172	0.000169	8.26E-08	LOC101117955,CPNE7,DPEP1,MT1B,MT4,SYT9,LOC101117691,MT3,LOC101117184
BP	response to zinc ion	0.00000126	0.000189	0.000185	3.14E-08	LOC101117955,MT1B,MT4,LOC101117691,MT3,LOC101117184
BP	regulation of immune effector process	0.0000116	0.00164	0.00161	0.00000218	LYN,LOC443423,IL1R1,HMGB1,LOC101110634,MIF,CD1,LOC105614484,MYO18A,LOC101114089,NECTIN4,LOC443441,LOC443442
BP	intracellular zinc ion homeostasis	0.0000126	0.00167	0.00164	0.000000544	LOC101117955,MT1B,MT4,LOC101117691,MT3,LOC101117184
BP	response to metal ion	0.0000209	0.00262	0.00257	0.00000249	LOC101117955,CPNE7,DPEP1,MT

						1B,MT4,SYT9,LOC101117691,MT3,LOC101117184
BP	antigen processing and presentation	0.0000241	0.00284	0.00279	0.00000346	LOC105602976,CD1,LOC443423,FCER1G,LOC105614484,LOC114108841,LOC101110634,LOC101114089,LOC443441,LOC443442
BP	regulation of T cell mediated immunity	0.0000252	0.00284	0.00279	0.00000308	CD1,LOC443423,IL1R1,LOC105614484,HMGB1,LOC101110634,LOC101114089,LOC443441,LOC443442
BP	regulation of leukocyte mediated immunity	0.0000302	0.00324	0.00318	0.00000508	LYN,CD1,LOC443423,IL1R1,LOC105614484,HMGB1,LOC101110634,LOC101114089,NECTIN4,LOC443441,LOC443442
BP	positive regulation of immune system process	0.0000354	0.00363	0.00356	0.0000116	LYN,LOC443423,FCER1G,IL1R1,HMGB1,LOC114108841,LOC101110634,MIF,LOC105602976,CD1,IL1RL2,HSPH1,TESPA1,SIN3A,TAFA3,LOC105614484,MYO18A,CD247,LOC101114089,LOC443441,LOC443442
BP	regulation of lymphocyte mediated immunity	0.0000685	0.00671	0.00659	0.0000113	CD1,LOC443423,IL1R1,LOC105614484,HMGB1,LOC101110634,LOC101114089,NECTIN4,LOC443441,LOC443442

BP	positive regulation of T cell mediated immunity	0.000129	0.0121	0.0119	0.0000167	CD1,LOC443423,IL1R1,LOC105614484,LOC101110634,LOC101114089,LOC443441,LOC443442
BP	detoxification	0.000213	0.0192	0.0188	0.0000176	LOC101117955,MT1B,MT4,LOC101117691,MT3,LOC101117184
BP	positive regulation of immune effector process	0.000233	0.02	0.0196	0.0000456	CD1,LOC443423,IL1R1,LOC105614484,MYO18A,MIF,LOC101110634,LOC101114089,LOC443441,LOC443442
BP	regulation of adaptive immune response based on somatic recombination of immune receptors built from immunoglobulin superfamily domains	0.00024	0.02	0.0196	0.0000408	CD1,LOC443423,IL1R1,LOC105614484,HMGB1,LOC101110634,LOC101114089,LOC443441,LOC443442
BP	antigen processing and presentation via MHC class Ib	0.000276	0.0222	0.0218	0.0000327	CD1,LOC443423,LOC105614484,LOC101110634,LOC101114089,LOC443441,LOC443442
BP	regulation of adaptive immune response	0.00033	0.0253	0.0248	0.0000593	CD1,LOC443423,IL1R1,LOC105614484,HMGB1,LOC101110634,LOC101114089,LOC443441,LOC443442
BP	antigen processing and presentation of endogenous antigen	0.00035	0.0253	0.0248	0.0000434	CD1,LOC443423,LOC105614484,LOC101110634,LOC101114089,LOC443441,LOC4434

						42
BP	regulation of leukocyte mediated cytotoxicity	0.000354	0.0253	0.0248	0.0000545	CD1,LOC443423,LOC105614484,LOC101110634,LOC101114089,NECTIN4,LOC443441,LOC443442
BP	regulation of T cell mediated cytotoxicity	0.00037	0.0253	0.0248	0.0000464	CD1,LOC443423,LOC105614484,LOC101110634,LOC101114089,LOC443441,LOC443442
BP	positive regulation of T cell mediated cytotoxicity	0.00037	0.0253	0.0248	0.0000464	CD1,LOC443423,LOC105614484,LOC101110634,LOC101114089,LOC443441,LOC443442
BP	regulation of cell killing	0.000406	0.0265	0.026	0.0000639	CD1,LOC443423,LOC105614484,LOC101110634,LOC101114089,NECTIN4,LOC443441,LOC443442
BP	regulation of cell activation	0.000412	0.0265	0.026	0.000106	LOC105602976,LYN,IL1RL2,FCER1G,HSPH1,TAFA3,TMX1,FANCA,MYO18A,HMGB1,LOC114108841,MIF
BP	positive regulation of adaptive immune response based on somatic recombination of immune receptors built from immunoglobulin superfamily domains	0.000443	0.0278	0.0272	0.0000709	CD1,LOC443423,IL1R1,LOC105614484,LOC101110634,LOC101114089,LOC443441,LOC443442
BP	regulation of immune system	0.00047	0.0286	0.0281	0.000207	LOC443423,PLAG1,HMGB1,LOC1

	process					14108841,LOC101110634,LOC105602976,IL1RL2,HSPH1,TESPA1,SIN3A,MYO18A,LYN,FCER1G,IL1R1,FANCA,MIF,CD1,TAFA3,LOC105614484,CD247,LOC101114089,NECTIN4,ZNF214,LOC443441,LOC443442
BP	positive regulation of adaptive immune response	0.000527	0.0313	0.0307	0.0000868	CD1,LOC443423,IL1R1,LOC105614484,LOC101110634,LOC101114089,LOC443441,LOC443442
BP	proline transmembrane transport	0.000579	0.0335	0.0328	0.00000386	SLC36A1,SLC36A3,SLC36A2
BP	positive regulation of lymphocyte mediated immunity	0.000889	0.0501	0.0492	0.00016	CD1,LOC443423,IL1R1,LOC105614484,LOC101110634,LOC101114089,LOC443441,LOC443442
BP	positive regulation of leukocyte mediated immunity	0.00123	0.0679	0.0666	0.000236	CD1,LOC443423,IL1R1,LOC105614484,LOC101110634,LOC101114089,LOC443441,LOC443442
BP	leukotriene metabolic process	0.00129	0.0691	0.0678	0.0000607	GGT5,ALOX5AP,DPEP1,LTA4H
BP	response to toxic substance	0.00134	0.0704	0.0691	0.00017	LOC101117955,MT1B,MT4,LOC101117691,MT3,LOC101117184
BP	amide metabolic process	0.00152	0.078	0.0765	0.000454	GGT5,LOC101111397,B4GALT3,HAL,GM2A,AMDHD1,LOC10111166

					4,DPEP1,PIPOX,ASNS,LOC101118990,PPCDC
BP	positive regulation of leukocyte mediated cytotoxicity	0.00163	0.0803	0.0788	0.000273	CD1,LOC443423,LOC105614484,LOC101110634,LOC101114089,LOC443441,LOC443442
BP	positive regulation of immune response	0.00164	0.0803	0.0788	0.000554	LYN,LOC443423,FCER1G,IL1R1,HMGB1,LOC101110634,CD1,TESPA1,SIN3A,LOC105614484,CD247,LOC101114089,LOC443441,LOC443442
BP	positive regulation of cell killing	0.00169	0.0813	0.0798	0.000287	CD1,LOC443423,LOC105614484,LOC101110634,LOC101114089,LOC443441,LOC443442
BP	icosanoid metabolic process	0.00193	0.0908	0.0891	0.000266	GGT5,ALOX5AP,DPEP1,LTA4H,MIF,PTGR2
BP	L-alanine transport	0.00263	0.121	0.119	0.0000525	SLC36A1,SLC36A3,SLC36A2
BP	regulation of leukocyte activation	0.00353	0.159	0.156	0.00101	LOC105602976,LYN,IL1RL2,HSPH1,TAFA3,FANCA,MYO18A,HMGB1,LOC114108841,MIF
BP	glycine transport	0.00417	0.181	0.178	0.000111	SLC36A1,SLC36A3,SLC36A2
BP	alanine transport	0.00417	0.181	0.178	0.000111	SLC36A1,SLC36A3,SLC36A2
BP	icosanoid biosynthetic process	0.00497	0.212	0.208	0.000392	GGT5,ALOX5AP,LTA4H,MIF
BP	positive regulation of response to	0.00528	0.221	0.216	0.00277	LOC443423,HMGB1,LOC10111063

	stimulus					4,DSTYK,LURAP1L,TESPA1,SIN3A,ZNF423,LYN,FCER1G,IL1R1,IL10RB,MIF,MT3,CD1,FRMD6,MC1R,TAFA3,TAOK1,LOC105614484,RPS20,CD247,LOC101114089,LOC443441,LOC443442
BP	carboxylic acid transmembrane transport	0.00543	0.223	0.219	0.000713	SLC36A1,SLC36A3,SLC36A2,SLC16A1,LOC101108016
BP	regulation of vesicle-mediated transport	0.00579	0.23	0.226	0.00165	RIMS2,LOC105612995,LYN,FCER1G,MYO18A,HMGB1,NF2,SYT9,EXPH5
BP	hematopoietic progenitor cell differentiation	0.00582	0.23	0.226	0.000487	LYN,DNAI4,SIN3A,HMGB1
BP	melanin metabolic process	0.00604	0.231	0.227	0.0002	MC1R,TYRP1,LOC101111911
BP	melanin biosynthetic process	0.00604	0.231	0.227	0.0002	MC1R,TYRP1,LOC101111911
BP	glutathione metabolic process	0.00659	0.248	0.243	0.000915	GGT5,LOC101111397,LOC101111664,DPEP1,LOC101118990
BP	pigment biosynthetic process	0.00675	0.249	0.245	0.000597	MC1R,TYRP1,LOC101111911,PPOX
BP	signal transduction in response to DNA damage	0.00723	0.263	0.258	0.00103	LYN,TAOK1,CHEK1,ATM,MIF
BP	regulation of multicellular organismal process	0.00763	0.273	0.268	0.00425	KCNE2,PLAG1,LAMA3,HMGB1,LOC114108841,SLC5A3,LOC105602976,IL1RL2,HSP

					H1,ANXA6,MYO18A,ZNF423,INSL5,LYN,FCER1G,IL1R1,FANCA,MIF,DEF8,MT3,CD2,MC1R,TMX1,TAFA3,NF2,ZNF214,EZH2
BP	nucleobase metabolic process	0.00775	0.273	0.268	0.000724	ALDH6A1,LOC101109035,DPYS,GART
BP	secondary metabolite biosynthetic process	0.00822	0.285	0.28	0.000326	MC1R,TYRP1,LOC101111911
BP	regulation of immune response	0.00862	0.295	0.289	0.00371	LYN,LOC443423,FCER1G,IL1R1,HMGB1,LOC101110634,CD1,TESPA1,SIN3A,LOC105614484,CD247,LOC101114089,NECTIN4,LOC443441,LOC443442
BP	regulation of platelet activation	0.00942	0.317	0.311	0.000405	LYN,FCER1G,TMX1
BP	carboxylic acid transport	0.0103	0.34	0.334	0.00252	SLC36A1,LYN,SLC36A3,SLC36A2,SLC16A1,SLC16A6,LOC101108016
BP	organic acid transport	0.0105	0.34	0.334	0.0026	SLC36A1,LYN,SLC36A3,SLC36A2,SLC16A1,SLC16A6,LOC101108016
BP	regulation of cell-cell adhesion	0.0107	0.34	0.334	0.00303	LOC105602976,LYN,IL1RL2,HSPH1,TMX1,HMGB1,LOC114108841,NF2
BP	amide catabolic process	0.0107	0.34	0.334	0.000495	HAL,AMDHD1,PIPOX
BP	small molecule	0.0111	0.349	0.342	0.00639	LOC101119648,D

	metabolic process					PYS,ODC1,MPI,B3GLCT,GADL1,SLC5A3,PPCDC,AMDHD1,MAN2C1,LTA4H,GGT5,LOC101109035,RRM2,ENTPD5,APOA2,PIPOX,ASNS,MIF,PTGR2,COQ6,ADCY10,ALDH6A1,HAL,ALOX5AP,DPEP1,GART
BP	regulation of ERK1 and ERK2 cascade	0.0115	0.357	0.35	0.00244	LYN,DSTYK,TNIP1,HMGB1,MIF,MT3
BP	pigment metabolic process	0.012	0.362	0.356	0.00132	MC1R,TYRP1,LOC101111911,PPOX
BP	leukotriene biosynthetic process	0.012	0.362	0.356	0.000596	GGT5,ALOX5AP,LTA4H
BP	intracellular signal transduction	0.0131	0.384	0.377	0.00707	LYN,WSB1,PRKCE,AMFR,DERL3,PPM1J,MIF,RHOC,GNA13,PPP3CA,RCAN1,ADCY10,ARHGAP30,MC1R,NUAK2,TAOK1,CHEK1,LOC101117683,PIK3C3,ATM,NF2,RGS9
BP	positive regulation of cellular process	0.0132	0.384	0.377	0.00926	HMGB1,LOC101110634,RIMS2,LOC105602976,DSTYK,LURAP1L,SIN3A,MYO18A,FCER1G,IL1R1,MIF,THOC5,RBMXL2,FRMD6,LOC101114089,IFNAR1,LOC443423,KCNE2,NR1I3,TYRP1,LOC114108841,

						SLC5A3,EGFR,IL1RL2,NEUROD4,HSPH1,TESPA1,ZNF423,ZNF783,EXPH5,LYN,IL10RB,DEF8,MT3,GRIN2B,CD2,CD1,MC1R,TNIP1,NFIA,TAFA3,TAOK1,LOC105614484,ATM,RPS20,NF2,FGF12,LOC443441,ZNF410,LOC443442,EZH2
BP	organic hydroxy compound transport	0.0133	0.384	0.377	0.00228	SLC16A1,APOA2,LOC101108016,SLC6A2,SLC5A3
BP	type I interferon-mediated signaling pathway	0.0135	0.384	0.377	0.00071	IFNAR2,SIN3A,IFNAR1
BP	negative regulation of growth	0.0143	0.403	0.396	0.0025	LOC101117955,MT1B,LOC101117691,MT3,LOC101117184
BP	cellular response to type I interferon	0.015	0.406	0.398	0.000837	IFNAR2,SIN3A,IFNAR1
BP	pyrimidine-containing compound catabolic process	0.015	0.406	0.398	0.000837	ALDH6A1,DPYS,ENTPD5
BP	protein phosphorylation	0.0151	0.406	0.398	0.00613	LYN,MOS,CDK18,DSTYK,NUAK2,TAOK1,PRKCE,CHEK1,LOC101117683,CDK10,EGFR,MAP4K4
BP	positive regulation of biological process	0.0152	0.406	0.398	0.0108	HMGB1,LOC101110634,RIMS2,LOC105602976,DSTYK,LURAP1L,SIN3A,MYO18A,INSL5,FCER1G,IL1R1,MIF,THOC5,RBMXL2,FRMD6

						,LOC101114089,GAS8,IFNAR1,LOC443423,KCNE2,NR1I3,TYRP1,LOC114108841,SLC5A3,EGFR,IL1RL2,NEUROD4,HSPH1,TESPA1,ZNF423,ZNF783,EXPH5,LYN,IL10RB,DEF8,MT3,GRIN2B,CD2,CD1,MC1R,TNIP1,NFIA,TAFA3,TAOK1,LOC105614484,ATM,RPS20,NF2,CD247,FGF12,LOC443441,ZNF410,LOC443442,EZH2
BP	positive regulation of leukocyte activation	0.0153	0.406	0.398	0.0041	LOC105602976,IL1RL2,HSPH1,TAFA3,HMGB1,LOC114108841,MIF
BP	regulation of lymphocyte activation	0.0162	0.425	0.417	0.00499	LOC105602976,LYN,IL1RL2,HSPH1,FANCA,HMGB1,LOC114108841,MIF
BP	regulation of hippo signaling	0.0165	0.428	0.42	0.000978	NUAK2,FRMD6,NF2
BP	homeostatic process	0.0168	0.431	0.423	0.0083	LYN,LOC101117955,LOC101119648,APOA2,HMGB1,SELENOK,MT4,MT3,USF1,SIN3A,TAOK1,ANXA6,FAM20A,MT1B,LOC101117691,LOC101117184,EZH2
BP	positive regulation of cell activation	0.0178	0.437	0.429	0.00493	LOC105602976,IL1RL2,HSPH1,TAFA3,HMGB1,LOC114108841,MIF

BP	organic cation transport	0.0181	0.437	0.429	0.00234	SLC36A1,SLC36A3,SLC36A2,SLC6A2
BP	interferon-mediated signaling pathway	0.0181	0.437	0.429	0.00113	IFNAR2,SIN3A,IFNAR1
BP	antigen processing and presentation of exogenous peptide antigen via MHC class II	0.0181	0.437	0.429	0.00113	LOC105602976,FCER1G,LOC114108841
BP	response to type I interferon	0.0181	0.437	0.429	0.00113	IFNAR2,SIN3A,IFNAR1
BP	phosphorylation	0.0182	0.437	0.429	0.00758	LYN,MOS,CDK18,DSTYK,NUAK2,TAOK1,PRKCE,CHEK1,LOC101117683,CDK10,EGFR,MAP4K4
BP	antigen processing and presentation of exogenous peptide antigen	0.0216	0.505	0.495	0.00149	LOC105602976,FCER1G,LOC114108841
BP	antigen processing and presentation of peptide antigen via MHC class II	0.0216	0.505	0.495	0.00149	LOC105602976,FCER1G,LOC114108841
BP	DNA damage checkpoint signaling	0.0217	0.505	0.495	0.00301	LYN,TAOK1,CHEK1,ATM
BP	response to stress	0.0221	0.508	0.498	0.0148	DERL3,PPM1J,HMGB1,ADAMTS4,GNA13,IL1RL2,HSPH1,NUAK2,SIN3A,CHEK1,ANXA6,TAC1,LOC101117184,GGT5,IFNAR2,LYN,LOC101117955,FCER1G,IL1R1,IL10RB,AMFR,FANCA,MIF,MT4,RHOC,MT3,PDIA4,CD2,RCAN1,MC1R,T

						NIP1,TAOK1,ATM,MT1B,NEIL1,LOC101117691,EZH2,IFNAR1
BP	alpha-amino acid metabolic process	0.0236	0.53	0.521	0.00697	ALDH6A1,HAL,AMDHD1,ODC1,DPEP1,PIPOX,ASNS
BP	regulation of tumor necrosis factor production	0.0237	0.53	0.521	0.00339	CD2,MC1R,HMGB1,MIF
BP	cellular response to chemical stimulus	0.0238	0.53	0.521	0.0128	LYN,LOC101117955,FCER1G,KCNE2,CPNE7,HMGB1,MT4,SYT9,MT3,USF1,GNAO1,TNIP1,SIN3A,DPEP1,ATM,MT1B,LOC101117691,LOC101117184,EZH2
BP	regulation of tumor necrosis factor superfamily cytokine production	0.0247	0.545	0.535	0.00359	CD2,MC1R,HMGB1,MIF
BP	cellular homeostasis	0.0253	0.554	0.543	0.0105	LOC101117955,SIN3A,TAOK1,ANXA6,SELENOK,MT1B,MT4,LOC101117691,MT3,LOC101117184,USF1
BP	regulation of cell adhesion	0.0257	0.558	0.548	0.00944	LOC105602976,LYN,IL1RL2,HSPH1,TMX1,LAMA3,HMGB1,LOC114108841,NF2
BP	intracellular chemical homeostasis	0.0268	0.57	0.559	0.0107	LOC101117955,SIN3A,ANXA6,SELENOK,MT1B,MT4,LOC101117691,MT3,LOC101117184,USF1

BP	adaptive immune response	0.0268	0.57	0.559	0.0107	LOC105602976,CD1,LOC443423,FCER1G,HMGB1,LOC114108841,CD247,LOC101114089,LOC443441,LOC443442
BP	L-amino acid metabolic process	0.0274	0.578	0.567	0.00722	ALDH6A1,HAL,AMDHD1,ODC1,PIPOX,ASNS
BP	positive regulation of lymphocyte activation	0.028	0.581	0.57	0.00742	LOC105602976,IL1RL2,HSPH1,HMGB1,LOC114108841,MIF
BP	inorganic ion homeostasis	0.0281	0.581	0.57	0.0105	LOC101117955,ANXA6,FAM20A,SELENOK,MT1B,MT4,LOC101117691,MT3,LOC101117184
BP	chemical homeostasis	0.0329	0.67	0.658	0.0156	LOC101117955,LOC101119648,APOA2,SELENOK,MT4,MT3,USF1,SIN3A,ANXA6,FAM20A,MT1B,LOC101117691,LOC101117184
BP	phenol-containing compound biosynthetic process	0.0333	0.67	0.658	0.00293	MC1R,TYRP1,LOC101111911
BP	neutral amino acid transport	0.0333	0.67	0.658	0.00293	SLC36A1,SLC36A3,SLC36A2
BP	proteinogenic amino acid metabolic process	0.0339	0.677	0.664	0.00943	ALDH6A1,HAL,AMDHD1,ODC1,PIPOX,ASNS
BP	regulation of localization	0.0343	0.678	0.665	0.0185	LOC105612995,LYN,ANKRD29,FCER1G,KCNE2,PRKCE,HMGB1,MIF,SYT9,SLC5A3,RIMS2,SIN3A,ANXA6,MYO18A,

						NF2,GAS8,EXPH5
BP	regulation of response to stimulus	0.0356	0.698	0.685	0.0245	LOC443423,HMGB1,LOC101110634,EGFR,IL1RL2,DSTYK,NUAK2,LURAP1L,TESPA1,SIN3A,ZNF423,RGS9,LYN,FCER1G,IL1R1,IL10RB,MIF,MT3,NLRP14,CD1,RCAN1,FRMD6,MC1R,TNIP1,TMX1,TAFA3,TAOK1,LOC105614484,ATM,RPS20,NF2,CD247,LOC101114089,NECTIN4,LOC443441,EZH2,LOC443442
BP	DNA integrity checkpoint signaling	0.0372	0.716	0.702	0.00638	LYN,TAOK1,CHEK1,ATM
BP	synaptic transmission, glutamatergic	0.0376	0.716	0.702	0.00356	UNC13B,GRIN2B,CDH8
BP	plasma membrane lactate transport	0.039	0.716	0.702	0.000583	SLC16A1,LOC101108016
BP	pyrimidine nucleobase catabolic process	0.039	0.716	0.702	0.000583	ALDH6A1,DPYS
BP	Fc receptor mediated stimulatory signaling pathway	0.039	0.716	0.702	0.000583	LYN,FCER1G
BP	lactate transport	0.039	0.716	0.702	0.000583	SLC16A1,LOC101108016
BP	lactate transmembrane transport	0.039	0.716	0.702	0.000583	SLC16A1,LOC101108016
BP	positive regulation of chemokine (C-X-C motif) ligand 2	0.039	0.716	0.702	0.000583	HMGB1,MIF

	production					
BP	positive regulation of T cell activation	0.0425	0.774	0.759	0.0104	LOC105602976,IL1RL2,HSPH1,HMGB1,LOC114108841
BP	cellular response to oxygen-containing compound	0.0439	0.791	0.776	0.019	GNAO1,LYN,TNIP1,SIN3A,DPEP1,ATM,HMGB1,MT3,USF1,EZH2
BP	positive regulation of tumor necrosis factor production	0.0445	0.791	0.776	0.00463	CD2,HMGB1,MIF
BP	multivesicular body sorting pathway	0.0445	0.791	0.776	0.00463	TMEM50B,CHMP1A,EXPH5
BP	regulation of cytokine production	0.0463	0.793	0.779	0.0192	CD2,LYN,IL1RL2,MC1R,PLAG1,IL1R1,HMGB1,MIF,ZNF214
BP	positive regulation of tumor necrosis factor superfamily cytokine production	0.0469	0.793	0.779	0.00503	CD2,HMGB1,MIF
BP	secondary metabolic process	0.0469	0.793	0.779	0.00503	MC1R,TYRP1,LOC101111911
BP	positive regulation of leukocyte cell-cell adhesion	0.0476	0.793	0.779	0.0121	LOC105602976,IL1RL2,HSPH1,HMGB1,LOC114108841
BP	L-amino acid catabolic process	0.048	0.793	0.779	0.00912	ALDH6A1,HAL,AMDHD1,PIPOX
BP	regulation of T cell activation	0.0485	0.793	0.779	0.0149	LOC105602976,IL1RL2,HSPH1,FANCA,HMGB1,LOC114108841
BP	positive regulation of cAMP/PKA signal transduction	0.0486	0.793	0.779	0.000966	MC1R,MIF
BP	skeletal muscle satellite cell migration	0.0486	0.793	0.779	0.000966	PPM1J,RHOC
BP	regulation of chemokine (C-X-C	0.0486	0.793	0.779	0.000966	HMGB1,MIF

	motif) ligand 2 production					
BP	L-histidine catabolic process to glutamate and formamide	0.0486	0.793	0.779	0.000966	HAL,AMDHD1
BP	L-histidine catabolic process to glutamate and formate	0.0486	0.793	0.779	0.000966	HAL,AMDHD1
BP	sulfur compound metabolic process	0.0497	0.802	0.787	0.0175	GGT5,LOC101111397,LOC101111664,DPEP1,PIPOX,LOC101118990,PPCDC
BP	response to oxygen-containing compound	0.0498	0.802	0.787	0.0244	GNAO1,LYN,TNIP1,SIN3A,DPEP1,ATM,HMGB1,GRIN2B,MT3,USF1,IFNAR1,EZH2
BP	organic anion transport	0.051	0.816	0.801	0.018	SLC36A1,LYN,SLC36A3,SLC36A2,SLC16A1,SLC16A6,LOC101108016
BP	antigen processing and presentation of peptide or polysaccharide antigen via MHC class II	0.0544	0.858	0.842	0.00634	LOC105602976,FCER1G,LOC114108841
BP	L-amino acid transport	0.0544	0.858	0.842	0.00634	SLC36A1,SLC36A3,SLC36A2
BP	monoatomic cation homeostasis	0.0566	0.86	0.844	0.0245	LOC101117955,ANXA6,FAM20A,SELENOK,MT1B,MT4,LOC101117691,MT3,LOC101117184
BP	regulation of phagocytosis	0.057	0.86	0.844	0.00682	FCER1G,MYO18A,HMGB1
BP	metabolic process	0.058	0.86	0.844	0.0497	LOC101116336,MPI,LOC101112774,VPREB3,LOC10

5602976,OGFOD1
,CHEK1,LOC1056
07745,LOC10111
8990,WSB1,PRK
CE,ENTPD5,PIPO
X,SDHC,MIF,TH
OC5,PDIA4,DPEP
1,SNRPF,RPP25,
NEIL1,LOC10111
8978,MTMR3,LO
C101111664,B3G
LCT,CUL1,SLC5
A3,NUAK2,HSPH
1,AMDHD1,ZMA
T5,LOC10110877
8,AMZ2,LTA4H,
GGT5,LOC10111
1397,LYN,ANKR
D29,CDK18,FAN
CA,ASNS,MT3,C
OQ6,ADCY10,AL
DH6A1,HAL,MC
1R,STT3A,CDK1
0,TMCC2,ATM,R
PS20,EZH2,B4GA
LT3,USPL1,PIGO
,LOC101119648,T
CF25,DPYS,GAD
L1,HMGB1,PPCD
C,ADAMTS4,DS
TYK,GM2A,SIN3
A,MYO18A,MAN
2C1,LOC1011132
64,LOC11411826
1,APOA2,WIPI1,P
TGR2,MRPS6,NI
PSNAP1,MMP11,
LANCL2,UFC1,N
DUFS2,PFDN2,PI
K3C3,GART,LOC
101116756,LOC1
14117284,EI24,SR
BD1,ODC1,TYRP
1,PPM1J,LOC114

						108841,SPG7,PPOX,COX5A,EGFR,TGS1,TMEM68,RPL13,LOC101117683,MAP4K4,LOC101109035,RRM2,USP21,AMFR,LOC101111911,MOS,NUDT21,NFIA,TAOK1,ALOX5AP
BP	regulation of toll-like receptor 2 signaling pathway	0.058	0.86	0.844	0.00144	LYN,HMGB1
BP	imidazole-containing compound catabolic process	0.058	0.86	0.844	0.00144	HAL,AMDHD1
BP	regulation of cAMP/PKA signal transduction	0.058	0.86	0.844	0.00144	MC1R,MIF
BP	formate metabolic process	0.058	0.86	0.844	0.00144	HAL,AMDHD1
BP	L-histidine catabolic process	0.058	0.86	0.844	0.00144	HAL,AMDHD1
BP	L-histidine metabolic process	0.058	0.86	0.844	0.00144	HAL,AMDHD1
BP	positive regulation of cell communication	0.0593	0.873	0.856	0.0335	LYN,IL1R1,IL10RB,HMGB1,MIF,GRIN2B,MT3,RIMS2,DSTYK,MC1R,FRMD6,LURAP1L,TESPA1,TAOK1,RPS20,ZNF423
BP	immunoglobulin production	0.0596	0.873	0.856	0.00732	VPREB3,LOC105602976,LOC114108841
BP	positive regulation of signaling	0.0602	0.875	0.859	0.0337	LYN,IL1R1,IL10RB,HMGB1,MIF,GRIN2B,MT3,RIMS2,DSTYK,MC1R,FRMD6,LURAP1L,TESPA1,TAOK1,RPS20,ZN

						F423
BP	phosphate-containing compound metabolic process	0.0622	0.895	0.878	0.0401	LYN,CDK18,LOC101118978,RRM2,MTMR3,PIGO,ENTPD5,PRKCE,MPI,PIPOX,PPM1J,PPCDC,EGFR,MOS,ADCY10,DSTYK,NUAK2,TAOK1,CHEK1,CDK10,LOC101117683,PIK3C3,GART,MAP4K4
BP	phosphorus metabolic process	0.0625	0.895	0.878	0.0403	LYN,CDK18,LOC101118978,RRM2,MTMR3,PIGO,ENTPD5,PRKCE,MPI,PIPOX,PPM1J,PPCDC,EGFR,MOS,ADCY10,DSTYK,NUAK2,TAOK1,CHEK1,CDK10,LOC101117683,PIK3C3,GART,MAP4K4
BP	monoatomic ion homeostasis	0.063	0.895	0.878	0.0277	LOC101117955,ANXA6,FAM20A,SELENOK,MT1B,MT4,LOC101117691,MT3,LOC101117184
BP	regulation of growth	0.0634	0.895	0.878	0.0209	LOC101117955,SIN3A,MT1B,LOC101117691,MT3,LOC101117184
BP	carboxylic acid metabolic process	0.0635	0.895	0.878	0.0344	GGT5,LOC101119648,ODC1,GADL1,PIPOX,ASNS,MIF,PTGR2,ALDH6A1,HAL,AMDHD1,ALOX5AP,DPEP1,LTA4H
BP	regulation of MAPK cascade	0.0642	0.899	0.883	0.0262	LYN,DSTYK,TNIP1,TAOK1,HMG

						B1,MIF,NF2,MT3
BP	negative regulation of cell cycle phase transition	0.0662	0.909	0.892	0.0186	LYN,TAOK1,CHEK1,ATM,EZH2
BP	oxoacid metabolic process	0.0666	0.909	0.892	0.0364	GGT5,LOC101119648,ODC1,GADL1,PIPOX,ASNS,MIF,PTGR2,ALDH6A1,HAL,AMDHD1,ALOX5AP,DPEP1,LTA4H
BP	negative regulation of cell activation	0.0668	0.909	0.892	0.0145	LYN,TAFA3,TMX1,HMGB1
BP	regulation of B cell apoptotic process	0.0673	0.909	0.892	0.002	LYN,MIF
BP	intracellular monoatomic ion homeostasis	0.0673	0.909	0.892	0.0278	LOC101117955,ANXA6,SELENOK,MT1B,MT4,LOC101117691,MT3,LOC101117184
BP	intracellular monoatomic cation homeostasis	0.0673	0.909	0.892	0.0278	LOC101117955,ANXA6,SELENOK,MT1B,MT4,LOC101117691,MT3,LOC101117184
BP	proteinogenic amino acid catabolic process	0.0685	0.92	0.902	0.015	ALDH6A1,HAL,AMDHD1,PIPOX
BP	production of molecular mediator of immune response	0.0705	0.94	0.923	0.00953	VPREB3,LOC105602976,LOC114108841
BP	regulated exocytosis	0.0737	0.978	0.96	0.0167	RIMS2,UNC13B,LYN,SYT9
BP	cellular component assembly	0.0748	0.987	0.968	0.0511	CCDC69,NTN4,HMGB1,LOC114108841,SPG7,CDH8,LOC105602976,SIN3A,OGFOD1,ZNF423,LOC101113264,BBS2,DNAI4,UNC13B,LOC114118261,WSB1,SG

						IP1,APOA2,WIPI1,MIF,TTC39C,DIAPH3,CDH12,SPIRE2,PIK3C3,SNRPF,GAS8,EZH2
BP	negative regulation of delayed rectifier potassium channel activity	0.0766	0.987	0.968	0.00265	ANKRD29,KCNE2
BP	imidazole-containing compound metabolic process	0.0766	0.987	0.968	0.00265	HAL,AMDHD1
BP	nucleobase catabolic process	0.0766	0.987	0.968	0.00265	ALDH6A1,DPYS
BP	regulation of platelet aggregation	0.0766	0.987	0.968	0.00265	LYN,TMX1
BP	alpha-amino acid catabolic process	0.0773	0.99	0.972	0.0178	ALDH6A1,HAL,AMDHD1,PIPOX
BP	regulation of cell migration	0.0804	1.00e+0	0.982	0.0369	LYN,GNA13,IL1R1,LAMA3,DPEP1,HMGB1,MIF,FGF12,EZH2
BP	regulation of type II interferon production	0.0819	1.00e+0	0.982	0.0121	CD2,IL1R1,HMGB1
BP	negative regulation of molecular function	0.0821	1.00e+0	0.982	0.0249	ANKRD29,DBNDD1,KCNE2,APOA2,MT3
BP	positive regulation of cell-cell adhesion	0.0832	1.00e+0	0.982	0.0254	LOC105602976,IL1RL2,HSPH1,HMGB1,LOC114108841
BP	positive regulation of biosynthetic process	0.0841	1.00e+0	0.982	0.0533	IL1R1,NR1I3,TYRP1,HMGB1,MIF,THOC5,MT3,SLC5A3,CD2,RBMXL2,IL1RL2,MC1R,HSPH1,NEUROD4,NFIA,ATM,ZNF423,FGF12,ZNF410,ZNF783
BP	cellular response to calcium ion	0.0849	1.00e+0	0.982	0.0128	CPNE7,DPEP1,SYT9

BP	wound healing	0.085	1.00e+0	0.982	0.026	GNA13,IL10RB,ANXA6,PPM1J,RHOC
BP	negative regulation of voltage-gated potassium channel activity	0.0857	1.00e+0	0.982	0.00339	ANKRD29,KCNE2
BP	negative regulation of potassium ion transmembrane transporter activity	0.0857	1.00e+0	0.982	0.00339	ANKRD29,KCNE2
BP	negative regulation of cell development	0.0865	1.00e+0	0.982	0.0209	LYN,HMGB1,NF2,MT3
BP	regulation of leukocyte migration	0.0903	1.00e+0	0.982	0.0222	LYN,IL1R1,HMGB1,MIF
BP	calcium-ion regulated exocytosis	0.0909	1.00e+0	0.982	0.0142	RIMS2,UNC13B,SYT9
BP	cellular component organization	0.092	1.00e+0	0.982	0.0737	USPL1,SMARCB1,THSD7B,TCF25,HMGB1,CDH8,CABIN1,ADAMTS4,LOC105602976,SIN3A,OGFOD1,ANXA6,MYO18A,PTGFRN,LOC101113264,UNC13B,LOC114118261,DNAI4,WSB1,SGIP1,APOA2,WIPI1,MIF,MMP11,CDH12,PIK3C3,FAM161B,SNRPF,GAS8,STOML2,MTMR3,LOC101109422,CCDC69,TYRP1,LAMA3,NTN4,SEZ6,SPG7,LOC114108841,CFAP126,NUAK2,NEUROD4,EPB41L3,ZNF423,BBS2,L

						YN,USP21,CHCHD10,TTC39C,MT3,DIAPH3,CAPZA1,TAOK1,SPIRE2,CDK10,ATM,NF2,XKR4,EZH2
BP	negative regulation of cell cycle process	0.0922	1.00e+0	0.982	0.0291	LYN,TAOK1,CHEK1,ATM,EZH2
BP	lymphocyte mediated immunity	0.0923	1.00e+0	0.982	0.0229	CD2,LOC105602976,FCER1G,LOC114108841
BP	regulation of intracellular signal transduction	0.0925	1.00e+0	0.982	0.0564	LYN,IL1R1,HMGB1,MIF,MT3,RCAN1,DSTYK,MC1R,NUAK2,FRMD6,LURAP1L,TNIP1,TAOK1,ATM,RPS20,NF2,EZH2
BP	regulation of inflammatory response	0.0937	1.00e+0	0.982	0.0297	LYN,NLRP14,IL1RL2,IL1R1,TAFA3
BP	organic acid metabolic process	0.0938	1.00e+0	0.982	0.0538	GGT5,LOC101119648,ODC1,GADL1,PIPOX,ASNS,MIF,PTGR2,ALDH6A1,HAL,AMDHD1,ALOX5AP,DPEP1,LTA4H
BP	hindbrain development	0.0939	1.00e+0	0.982	0.015	SEZ6,ZNF423,EZH2
BP	positive regulation of intracellular signal transduction	0.0945	1.00e+0	0.982	0.0492	LYN,DSTYK,MC1R,FRMD6,LURAP1L,IL1R1,TAOK1,RPS20,HMGB1,MIF,MT3
BP	negative regulation of potassium ion transmembrane transport	0.0948	1.00e+0	0.982	0.00421	ANKRD29,KCNE2
BP	glutathione catabolic process	0.0948	1.00e+0	0.982	0.00421	GGT5,DPEP1
BP	regulation of homotypic	0.0948	1.00e+0	0.982	0.00421	LYN,TMX1

	cell-cell adhesion					
BP	negative regulation of multicellular organismal process	0.0952	1.00e+0	0.982	0.0456	LYN,MC1R,TAFA3,TMX1,HMGB1,NF2,ZNF423,MT3,EZH2
BP	regulation of transport	0.0961	1.00e+0	0.982	0.0539	LOC105612995,LYN,ANKRD29,FCER1G,KCNE2,PRKCE,HMGB1,MIF,SYT9,RIMS2,MYO18A,NF2,EXPH5
BP	regulation of monoatomic cation transmembrane transport	0.0962	1.00e+0	0.982	0.0243	LYN,ANKRD29,KCNE2,PRKCE
BP	homophilic cell-cell adhesion	0.0997	1.00e+0	0.982	0.0323	CDH12,CDHR3,FAT2,NECTIN4,CDH8
BP	positive regulation of signal transduction	0.0999	1.00e+0	0.982	0.0579	LYN,IL1R1,IL10RB,HMGB1,MIF,MT3,DSTYK,MC1R,FRMD6,LURAP1L,TESPA1,TAOK1,RPS20,ZNF423
CC	cytoplasm	0.00000115	0.000436	0.000426	0.000000881	CPNE7,LOC101116336,MPI,LOC101112774,LOC101110634,SCAMP5,RIMS2,RUSC2,OGFOD1,CHEK1,LOC105607745,PTGFRN,LOC101118990,RGS9,PKNOX2,UNC13B,LOC101117955,WSB1,PRKCE,ENTPD5,PIPOX,SDHC,MIF,PDIA4,FRMD6,SYPL1,TMX1,PRKAR1A,FEZ1,VOPP1,BBOF1,SNRPF,MT1B,NEIL1,I

FNAR1,MTMR3,
CABP7,LOC1011
11664,B3GLCT,T
EX26,SLC5A3,N
UAK2,HSPH1,TE
SPA1,AMDHD1,
EPB41L3,LOC101
108778,ARSG,LT
A4H,EXPH5,BBS
2,LOC101111397,
LYN,CDK18,TM
EM50B,CHCHD1
0,ASNS,MT4,SYT
9,MT3,GRIN2B,C
OQ6,GNAO1,MO
V10,RCAN1,ADC
Y10,LOC1011202
51,ALDH6A1,HA
L,DIAPH3,SPIRE
2,STT3A,NF1,CD
K10,FAT2,TMCC
2,ATM,LOC10560
9617,RPS20,NF2,
FGF12,B4GALT3,
PIGO,LOC101119
648,DPYS,GADL
1,HMGB1,RCSD1
,PPCDC,MEDAG,
PPP3CA,DSTYK,
CHMP1A,ANXA6
,MYO18A,MAN2
C1,LOC10111326
4,LOC101117184,
SLC36A1,DYNLT
5,DNAI4,SLC36A
3,SLC36A2,VPS9
D1,SGIP1,WIPI1,
RHOC,PTGR2,M
RPS6,NIPSNAP1,
TOMM40L,DCST
AMP,LANCL2,U
FC1,NDUFS2,PF
DN2,PIK3C3,FA
M20A,LOC10111

						4089,GART,GAS8,LOC101116756,LOC443423,STOML2,LOC114117284,LOC101109422,NUFIP2,PLAG1,EI24,DERL3,NR1I3,CCDC69,ODC1,TYRP1,SEZ6,PPM1J,SPG7,PPOX,SELENOK,COX5A,EGFR,GNA13,CREG1,TGS1,TMEM68,RPL13,LOC101117683,DEDD,MAP4K4,LOC101109035,RRM2,USP21,AMFR,LOC101111911,SPATA2L,CD2,NLRP14,NUDT21,DBNDD1,CD1,ZNF70,TNIP1,CAPZA1,TAOK1,ALOX5AP,CD247,LOC101117691,LOC443441,LOC443442
CC	intracellular anatomical structure	0.0000815	0.0154	0.015	0.0000702	SMARCB1,CPNE7,LOC101110634,SCAMP5,RUSC2,OGFOD1,CHEK1,PTGFRN,PKNOX2,UNC13B,LOC101117955,WSB1,PRKCE,ENTPD5,MIF,PRKAR1A,FEZ1,VOPP1,MT1B,NEIL1,ZNF398,ZNF276,MTMR3,B3GLCT,TEX26,SLC5A3,CFAP126,LOC101104855,NUAK2,HSPH1,TESPA1,EPB41L3,

LOC101108778,A
RSG,LTA4H,EXP
H5,BBS2,LOC101
111397,LYN,CDK
18,TMEM50B,GR
IN2B,ATOSB,MO
V10,ALDH6A1,H
AL,LOC10110612
9,CDK10,FAT2,T
MCC2,ATM,RPS
20,LOC10560961
7,CENPBD1,EZH
2,VSX2,USPL1,PI
GO,TCF25,DPYS,
RCSD1,PPCDC,M
EDAG,PPP3CA,D
STYK,CHMP1A,
ANXA6,LOC1011
13264,LOC10111
7184,LIN52,RHO
C,PTGR2,MRPS6,
TOMM40L,DCST
AMP,UFC1,FAM
20A,GART,GAS8,
LOC101116756,L
OC443423,LOC10
1109422,DERL3,
NR1I3,ODC1,SEZ
6,COX5A,EGFR,
CREG1,TMEM68,
RPL13,DEDD,MA
P4K4,NLRP14,N
UDT21,ZNF70,NF
IA,CAPZA1,TAO
K1,ZNF215,ZNF2
14,LOC443441,L
OC443442,MPI,L
OC101116336,LO
C101112774,CEP
128,RIMS2,LOC1
05607745,LOC10
1118990,RGS9,PI
POX,SDHC,THO
C5,PDIA4,RBMX

L2,FRMD6,SYPL
1,TMX1,SNRPF,F
AM161B,BBOF1,
IFNAR1,LOC105
612995,LOC1011
18978,CABP7,LO
C101111664,NEU
ROD4,AMDHD1,
ZMAT5,ZNF423,
ZNF786,ZNF783,
POU2F1,ZBTB14,
FANCA,ASNS,C
HCHD10,MT4,SY
T9,MT3,COQ6,G
NAO1,LOC10112
0251,RCAN1,AD
CY10,DIAPH3,ST
T3A,SPIRE2,NF1,
NF2,FGF12,ZNF4
10,B4GALT3,LO
C101119648,MID
EAS,GADL1,HM
GB1,CABIN1,AD
AMTS4,SIN3A,M
YO18A,MAN2C1,
LOC114118261,D
YNLT5,DNAI4,C
CDC38,SLC36A1,
KLF12,SLC36A3,
SLC36A2,VPS9D
1,SGIP1,WIPI1,N
AV2,NIPSNAP1,
LANCL2,NDUFS
2,PFDN2,PIK3C3,
LOC101114089,L
OC114117284,ST
OML2,NUFIP2,P
LAG1,EI24,CCDC
69,TYRP1,PPM1J,
SPG7,PPOX,SEL
ENOK,GNA13,T
GS1,LOC1011176
83,LOC10110903
5,RRM2,USP21,A

						MFR,LOC101111911,USF1,SPATA2L,CD2,CD1,DBNDD1,TNIP1,ALOX5AP,CD247,LOC101117691
CC	vacuolar membrane	0.00104	0.131	0.128	0.000274	SLC36A1,CD1,LOC443423,SLC36A3,SLC36A2,ANXA6,VOPP1,LOC101110634,LOC101114089,LOC443441,LOC443442
CC	endosome	0.00205	0.133	0.13	0.000876	LOC443423,SLC36A2,WIPI1,HMGB1,LOC101110634,RCSD1,EGFR,SCAMP5,CD1,CHMP1A,ANXA6,VOPP1,PIK3C3,LOC101114089,NF2,LOC443441,EXPH5,IFNAR1,LOC443442
CC	membrane-bounded organelle	0.00211	0.133	0.13	0.00178	SMARCB1,CPNE7,LOC101116336,LOC101112774,LOC101110634,SCAMP5,RUSC2,OGFOD1,CHEK1,LOC105607745,PTGFRN,PKNOX2,UNC13B,LOC101117955,PRKCE,ENTPD5,PIPOX,SDHC,MIF,THOC5,PDIA4,RBMXL2,SYPL1,TMX1,VOPP1,FAM161B,BBOF1,SNRPF,MT1B,NEIL1,ZNF398,IFNAR1,LOC105612995,ZNF276,LOC101118978,C

ABP7,B3GLCT,C
FAP126,NEURO
D4,LOC10110485
5,HSPH1,ZMAT5,
LOC101108778,A
RSG,LTA4H,ZNF
423,ZNF786,ZNF
783,EXPH5,BBS2
,LYN,CDK18,PO
U2F1,ZBTB14,T
MEM50B,FANCA
,CHCHD10,MT4,
SYT9,MT3,GRIN
2B,COQ6,ATOSB
,MOV10,RCAN1,
ADCY10,LOC101
120251,ALDH6A
1,LOC101106129,
SPIRE2,STT3A,N
F1,CDK10,FAT2,
TMCC2,ATM,LO
C105609617,NF2,
CENPBD1,ZNF41
0,VSX2,EZH2,B4
GALT3,USPL1,PI
GO,LOC10111964
8,TCF25,MIDEAS
,HMGB1,RCSD1,
CABIN1,ADAMT
S4,SIN3A,CHMP
1A,ANXA6,MYO
18A,MAN2C1,LO
C101113264,LOC
101117184,LIN52,
CCDC38,SLC36A
1,LOC114118261,
DNAI4,KLF12,SL
C36A3,SLC36A2,
VPS9D1,SGIP1,W
IPI1,NAV2,RHOC
,MRPS6,NIPSNA
P1,TOMM40L,DC
STAMP,LANCL2,
NDUFS2,PIK3C3,

						FAM20A,LOC101114089,GAS8,LOC101116756,LOC443423,STOML2,LOC101109422,NUFIP2,PLAG1,EI24,DERL3,NR1I3,CCDC69,TYRP1,SEZ6,PPM1J,SPG7,PPOX,SELENOK,COX5A,EGFR,GNA13,TGS1,TMEM68,LOC101117683,DEDD,LOC101109035,USP21,AMFR,USF1,CD2,NUDT21,CD1,ZNF70,TNIP1,NFIA,TAOK1,ALOX5AP,ZNF215,ZNF214,LOC101117691,LOC443441,LOC443442
CC	intracellular membrane-bounded organelle	0.00225	0.133	0.13	0.00188	SMARCB1,CPNE7,LOC101116336,LOC101112774,LOC101110634,SCAMP5,RUSC2,OGFOD1,CHEK1,LOC105607745,PTGFRN,PKNOX2,UNC13B,LOC101117955,PRKCE,ENTPD5,PIPOX,SDHC,MIF,THOC5,PDIA4,RBMXL2,SYPL1,TMX1,VOPP1,SNRPF,MT1B,NEIL1,ZNF398,IFNAR1,LOC105612995,ZNF276,LOC101118978,CABP7,B3GLCT,NEUROD4,LOC1011

04855,HSPH1,ZM
AT5,LOC1011087
78,ARSG,LTA4H,
ZNF423,ZNF786,
ZNF783,EXPH5,L
YN,CDK18,POU2
F1,ZBTB14,TME
M50B,FANCA,C
HCHD10,MT4,SY
T9,MT3,GRIN2B,
COQ6,ATOSB,M
OV10,RCAN1,AD
CY10,LOC101120
251,ALDH6A1,L
OC101106129,SPI
RE2,STT3A,NF1,
CDK10,FAT2,TM
CC2,ATM,LOC10
5609617,NF2,CE
NPBD1,ZNF410,
VSX2,EZH2,B4G
ALT3,USPL1,PIG
O,LOC101119648,
TCF25,MIDEAS,
HMGB1,RCSD1,
CABIN1,ADAMT
S4,SIN3A,CHMP
1A,ANXA6,MYO
18A,MAN2C1,LO
C101113264,LOC
101117184,LIN52,
SLC36A1,LOC11
4118261,KLF12,S
LC36A3,SLC36A
2,VPS9D1,SGIP1,
WIPI1,NAV2,RH
OC,MRPS6,NIPS
NAP1,TOMM40L,
DCSTAMP,LANC
L2,NDUFS2,PIK3
C3,FAM20A,LOC
101114089,GAS8,
LOC101116756,L
OC443423,STOM

						L2,LOC101109422,NUFIP2,PLAG1,EI24,DERL3,NR1I3,CCDC69,TYRP1,SEZ6,PPM1J,SPG7,PPOX,SELENOK,COX5A,EGFR,GNA13,TGS1,TMEM68,LOC101117683,DEDD,LOC101109035,USP21,AMFR,USF1,CD2,NUDT21,CD1,ZNF70,TNIP1,NFIA,TAOK1,ALOX5AP,ZNF215,ZNF214,LOC101117691,LOC443441,LOC443442
CC	cell surface	0.00246	0.133	0.13	0.001	LOC443423,FCER1G,KCNE2,IL1R1,HMGB1,LOC101110634,MIF,EGFR,PDIA4,CD2,DCSTAMP,CD1,LOC105614484,LOC101114089,MPZL1,LOC443441,LOC443442
CC	endomembrane system	0.00296	0.14	0.136	0.00199	B4GALT3,PIGO,LOC101119648,LOC101116336,HMGB1,LOC101110634,RCSD1,SCAMP5,CHMP1A,ANXA6,MYO18A,LOC105607745,PTGFRN,POGLUT3,UNC13B,SLC36A2,SGIP1,ENTPD5,WIPI1,PDIA4,DCSTAMP,SYPL1,TMX1,VOPP1,PIK3C3,FAM20A,L

						OC101114089,GAS8,IFNAR1,LOC101116756,LOC443423,LOC101109422,CABP7,EI24,DERL3,B3GLCT,SEZ6,SELENOK,EGFR,CREG1,TMEM68,LOC101117683,EXPH5,LYN,AMFR,TMEM50B,SYT9,MT3,GRIN2B,COQ6,CD2,CD1,ALOX5AP,STT3A,FAT2,TMCC2,NF2,LOC443441,LOC443442
CC	side of membrane	0.00332	0.14	0.136	0.00133	LYN,LOC443423,FCER1G,IL1R1,LOC101110634,GNAO1,CD2,GNA13,CD1,FRMD6,GM2A,LOC105614484,DPEP1,LOC101114089,LOC443441,LOC443442
CC	endosome membrane	0.00387	0.146	0.143	0.00131	SCAMP5,CD1,LOC443423,SLC36A2,CHMP1A,ANXA6,VOPP1,LOC101110634,LOC101114089,EGFR,LOC443441,LOC443442
CC	extrinsic component of membrane	0.00476	0.163	0.159	0.00116	GNAO1,LYN,GNA13,CDH12,WIPI1,PIK3C3,CDH8,COQ6
CC	perinuclear region of cytoplasm	0.0052	0.163	0.159	0.00159	LYN,ADCY10,CABP7,TAOK1,PRKCE,ANXA6,NF2,MT3,SLC5A3,EGFR
CC	cytoplasmic	0.00598	0.163	0.159	0.0034	LOC443423,TYR

	vesicle					P1,HMGB1,LOC101110634,RCSD1,EGFR,SCAMP5,RUSC2,CHMP1A,ANXA6,EXPH5,UNC13B,SLC36A2,VPS9D1,SGIP1,WIPI1,SYT9,MT3,PDIA4,CD1,SYPL1,SPIRE2,VOPP1,PIK3C3,ATM,NF2,LOC101114089,LOC443441,IFNAR1,LOC443442
CC	intracellular vesicle	0.00602	0.163	0.159	0.00342	LOC443423,TYRP1,HMGB1,LOC101110634,RCSD1,EGFR,SCAMP5,RUSC2,CHMP1A,ANXA6,EXPH5,UNC13B,SLC36A2,VPS9D1,SGIP1,WIPI1,SYT9,MT3,PDIA4,CD1,SYPL1,SPIRE2,VOPP1,PIK3C3,ATM,NF2,LOC101114089,LOC443441,IFNAR1,LOC443442
CC	cytoplasmic vesicle membrane	0.0117	0.283	0.276	0.00562	UNC13B,LOC443423,SLC36A2,TYRP1,LOC101110634,SYT9,EGFR,SCAMP5,CD1,SYPL1,CHMP1A,SPIRE2,ANXA6,VOPP1,LOC101114089,LOC443441,LOC443442
CC	lysosomal membrane	0.0127	0.283	0.276	0.00372	CD1,LOC443423,ANXA6,VOPP1,LOC101110634,LOC101114089,LOC443441,LOC4434

						42
CC	lytic vacuole membrane	0.0127	0.283	0.276	0.00372	CD1,LOC443423,ANXA6,VOPP1,LOC101110634,LOC101114089,LOC443441,LOC443442
CC	vesicle membrane	0.0136	0.285	0.278	0.00655	UNC13B,LOC443423,SLC36A2,TYRP1,LOC101110634,SYT9,EGFR,SCAMP5,CD1,SYPL1,CHMP1A,SPIRE2,ANXA6,VOPP1,LOC101114089,LOC443441,LOC443442
CC	intracellular organelle	0.0175	0.348	0.34	0.0155	SMARCB1,CPNE7,LOC101116336,LOC101112774,LOC101110634,CEP128,SCAMP5,RIMS2,RUSC2,OGFOD1,CHEK1,LOC105607745,PTGFRN,PKNOX2,UNC13B,LOC101117955,PRKCE,ENTPD5,PIPOX,SDHC,MIF,THOC5,PDIA4,RBMXL2,FRMD6,SYPL1,TMX1,VOPP1,FAM161B,BBOF1,SNRPF,MT1B,NEIL1,ZNF398,IFNAR1,LOC105612995,ZNF276,LOC101118978,CABP7,B3GLCT,CFAP126,NEUROD4,LOC101104855,HSPH1,ZMAT5,EPB41L3,LOC101108778,A

RSG,LTA4H,ZNF
423,ZNF786,ZNF
783,EXPH5,BBS2
,LYN,CDK18,PO
U2F1,ZBTB14,T
MEM50B,FANCA
,CHCHD10,MT4,
SYT9,MT3,GRIN
2B,COQ6,ATOSB
,MOV10,RCAN1,
ADCY10,LOC101
120251,ALDH6A
1,DIAPH3,LOC10
1106129,SPIRE2,
STT3A,NF1,CDK
10,FAT2,TMCC2,
ATM,LOC105609
617,RPS20,NF2,C
ENPBD1,ZNF410,
VSX2,EZH2,B4G
ALT3,USPL1,PIG
O,LOC101119648,
TCF25,MIDEAS,
HMGB1,RCSD1,
CABIN1,ADAMT
S4,SIN3A,CHMP
1A,ANXA6,MYO
18A,MAN2C1,LO
C101113264,LOC
101117184,LIN52,
CCDC38,SLC36A
1,LOC114118261,
DYNLT5,DNAI4,
KLF12,SLC36A3,
SLC36A2,VPS9D
1,SGIP1,WIPI1,N
AV2,RHOC,MRP
S6,NIPSNAP1,TO
MM40L,DCSTA
MP,LANCL2,ND
UFS2,PIK3C3,FA
M20A,LOC10111
4089,GAS8,LOC1
01116756,LOC44

						3423,STOML2,LOC114117284,LOC101109422,NUFIP2,PLAG1,EI24,DERL3,NR1I3,CCDC69,TYRP1,SEZ6,PPM1J,SPG7,PPOX,SELENOK,COX5A,EGFR,GNA13,TGS1,TMEM68,RPL13,LOC101117683,DEDD,LOC101109035,USP21,AMFR,USF1,CD2,NUDT21,CD1,ZNF70,TNIP1,NFIA,CAPZA1,TAOK1,ALOX5AP,ZNF215,ZNF214,LOC101117691,LOC443441,LOC443442
CC	vesicle	0.0191	0.361	0.352	0.0118	LOC443423,TYRP1,HMGB1,LOC101110634,RCSD1,EGFR,SCAMP5,RUSC2,CHMP1A,ANXA6,EXPH5,UNC13B,SLC36A2,VPS9D1,SGIP1,WIPI1,SYT9,MT3,PDIA4,CD1,SYPL1,SPIRE2,VOPP1,PIK3C3,ATM,NF2,LOC101114089,LOC443441,IFNAR1,LOC443442
CC	vacuole	0.021	0.379	0.37	0.00939	SLC36A1,LOC443423,SLC36A3,SLC36A2,LOC101110634,CD1,ANXA6,VOPP1,ARSG,LOC101114089,LOC443441,IFNAR

						1,LOC443442
CC	external side of plasma membrane	0.0255	0.438	0.428	0.0101	CD2,CD1,LOC443423,FCER1G,IL1R1,LOC105614484,LOC101110634,LOC101114089,LOC443441,LOC443442
CC	Fc receptor complex	0.0274	0.45	0.439	0.000254	FCER1G,CD247
CC	cytoplasmic side of plasma membrane	0.0287	0.451	0.441	0.0062	GNAO1,LYN,GNA13,FRMD6,GM2A
CC	endoplasmic reticulum	0.0305	0.461	0.45	0.019	LOC101116756,PIGO,LOC101119648,EI24,DERL3,LOC101116336,B3GLCT,SEZ6,HMGB1,SELENOK,EGFR,TMEM68,LOC105607745,LOC101117683,PTGFRN,SLC36A2,ENTPD5,AMFR,TMEM50B,GRIN2B,PDIA4,DCSTAMP,TMX1,ALOX5AP,STT3A,TMCC2,FAM20A
CC	transcription repressor complex	0.0391	0.563	0.55	0.00378	SIN3A,HMGB1,LIN52
CC	organelle membrane	0.0402	0.563	0.55	0.0291	B4GALT3,LOC443423,STOML2,PIGO,LOC101119648,CABP7,DERL3,TYRP1,B3GLCT,LOC101110634,SPG7,SELENOK,PPOX,COX5A,EGFR,SCAMP5,CHMP1A,TMEM68,ANXA6,LOC101108778,PTGFRN,LOC101113264,BB

CC	organelle	0.0424	0.564	0.551	0.0383	S2,SLC36A1,UNC13B,SLC36A3,SLC36A2,AMFR,SDHC,SYT9,GRIN2B,COQ6,TOMM40L,CD1,DCSTAMP,SYPL1,TMX1,ALOX5AP,STT3A,SPIRE2,VOPP1,NDUFS2,LOC101114089,LOC443441,LOC443442 SMARCB1,CPNE7,LOC101116336,LOC101112774,LOC101110634,CEP128,SCAMP5,RIMS2,RUSC2,OGFOD1,CHEK1,LOC105607745,PTGFRN,PKNOX2,UNC13B,LOC101117955,PRKCE,ENTPD5,PIPOX,SDHC,MIF,THOC5,PDIA4,RBMXL2,FRMD6,SYPL1,TMX1,VOPP1,FAM161B,BBOF1,SNRPF,MT1B,NEIL1,ZNF398,IFNAR1,LOC105612995,ZNF276,LOC101118978,CABP7,B3GLCT,CFAP126,NEUROD4,LOC101104855,HSPH1,ZMAT5,EPB41L3,LOC101108778,ARSG,LTA4H,ZNF423,ZNF786,ZNF783,EXPH5,BBS2,LYN,CDK18,POU2F1,ZBTB14,T

MEM50B,FANCA
,CHCHD10,MT4,
SYT9,MT3,GRIN
2B,COQ6,ATOSB
,MOV10,RCAN1,
ADCY10,LOC101
120251,ALDH6A
1,DIAPH3,LOC10
1106129,SPIRE2,
STT3A,NF1,CDK
10,FAT2,TMCC2,
ATM,LOC105609
617,RPS20,NF2,C
ENPBD1,ZNF410,
VSX2,EZH2,B4G
ALT3,USPL1,PIG
O,LOC101119648,
TCF25,MIDEAS,
HMGB1,RCSD1,
CABIN1,ADAMT
S4,SIN3A,CHMP
1A,ANXA6,MYO
18A,DLGAP1,MA
N2C1,LOC101113
264,LOC1011171
84,LIN52,CCDC3
8,SLC36A1,LOC1
14118261,DYNLT
5,DNAI4,KLF12,S
LC36A3,SLC36A
2,VPS9D1,SGIP1,
WIPI1,NAV2,RH
OC,MRPS6,NIPS
NAP1,TOMM40L,
DCSTAMP,LANC
L2,NDUFS2,PIK3
C3,FAM20A,LOC
101114089,GAS8,
LOC101116756,L
OC443423,STOM
L2,LOC11411728
4,LOC101109422,
NUFIP2,PLAG1,E
I24,DERL3,NR1I3

						,CCDC69,TYRP1,SEZ6,PPM1J,SPG7,PPOX,SELENOK,COX5A,EGFR,GNA13,TGS1,TMEM68,RPL13,LOC101117683,DEDD,LOC101109035,USP21,AMFR,USF1,CD2,NUDT21,CD1,ZNF70,TNIP1,NFIA,CAPZA1,TAOK1,ALOX5AP,ZNF215,ZNF214,LOC101117691,LOC443441,LOC443442
CC	extrinsic component of plasma membrane	0.0445	0.564	0.551	0.011	GNAO1,LYN,GNA13,CDH12,CDH8
CC	cell junction	0.0448	0.564	0.551	0.0286	LOC105612995,CPNE7,SEZ6,SLC6A2,CDH8,GNA13,RIMS2,SCAMP5,DSTYK,ANXA6,DLGAP1,TAC1,MPZL1,UNC13B,ANKRD29,SGIP1,GRIN2B,MT3,SDK1,SYPL1,CDHR3,CDH12,FAT2,RPS20,NF2,NECTIN4
CC	cytoplasmic side of membrane	0.0492	0.588	0.574	0.0126	GNAO1,LYN,GNA13,FRMD6,GM2A
CC	plasma membrane region	0.0498	0.588	0.574	0.028	BBS2,UNC13B,SLC16A1,KCNE2,LOC101108016,PPM1J,RHOC,GRIN2B,SLC6A2,SLC5A3,EGFR,GNA13,RIMS2,DSTYK,FRMD6,DPEP1

						,NF2
CC	apical part of cell	0.0652	0.738	0.72	0.0269	GNA13,DSTYK,KCNE2,SLC16A1,FRMD6,DPEP1,NF2,SLC5A3
CC	presynapse	0.0664	0.738	0.72	0.0294	RIMS2,SCAMP5,UNC13B,LOC105612995,SYPL1,SGIP1,MT3,SLC6A2,CDH8
CC	synapse	0.0695	0.742	0.724	0.0416	UNC13B,LOC105612995,ANKRD29,SGIP1,CPNE7,SEZ6,GRIN2B,MT3,SLC6A2,CDH8,GNA13,RIMS2,SCAMP5,SDK1,SYPL1,DLGAP1,RPS20,TAC1
CC	cleavage furrow	0.0723	0.742	0.724	0.00994	PPM1J,NF2,RHOC
CC	lysosome	0.0733	0.742	0.724	0.0349	CD1,LOC443423,ANXA6,VOPP1,ARSG,LOC101110634,LOC101114089,LOC443441,IFNAR1,LOC443442
CC	lytic vacuole	0.0746	0.742	0.724	0.0357	CD1,LOC443423,ANXA6,VOPP1,ARSG,LOC101110634,LOC101114089,LOC443441,IFNAR1,LOC443442
CC	membrane protein complex	0.0788	0.764	0.745	0.0508	FCER1G,KCNE2,DERL3,SDHC,HMGB1,LOC114108841,SPG7,GRIN2B,COX5A,SLC5A3,TOMM40L,CDH8,GNAO1,LOC105602976,GNA13,CHMP1A,CDH12,NDUFS2,LOC101108778,PIK3C

						3,CD247,LOC101113264
MF	endogenous lipid antigen binding	4.27E-09	0.000000891	0.000000866	5.69E-11	CD1,LOC443423,LOC105614484,LOC101110634,LOC101114089,LOC443441,LOC443442
MF	lipid antigen binding	4.27E-09	0.000000891	0.000000866	5.69E-11	CD1,LOC443423,LOC105614484,LOC101110634,LOC101114089,LOC443441,LOC443442
MF	exogenous lipid antigen binding	4.27E-09	0.000000891	0.000000866	5.69E-11	CD1,LOC443423,LOC105614484,LOC101110634,LOC101114089,LOC443441,LOC443442
MF	lipopeptide binding	0.000000038	0.00000595	0.00000577	8.09E-10	CD1,LOC443423,LOC105614484,LOC101110634,LOC101114089,LOC443441,LOC443442
MF	solute:proton symporter activity	0.0000245	0.00307	0.00298	0.000000682	SLC36A1,SLC36A3,SLC36A2,SLC16A1,LOC101108016
MF	amino acid:proton symporter activity	0.000252	0.0263	0.0256	0.000000784	SLC36A1,SLC36A3,SLC36A2
MF	antigen binding	0.000334	0.0298	0.029	0.0000411	CD1,LOC443423,LOC105614484,LOC101110634,LOC101114089,LOC443441,LOC443442
MF	binding	0.000596	0.0467	0.0453	0.000523	SMARCB1,CPNE7,MPI,LOC101112774,LOC101110634,CDH8,RIMS2,LOC105602976,RUSC2,OGFOD1,C

HEK1,LOC10560
7745,POGLUT3,P
KNOX2,UNC13B,
IFNAR2,LOC101
117955,WSB1,ZN
F282,IL1R1,PRK
CE,ENTPD5,IL1R
2,HPCAL1,PIPOX
,SDHC,MIF,THO
C5,RBMXL2,PRK
AR1A,DPEP1,SN
RPF,MT1B,RPP2
5,NEIL1,ZNF398,
IFNAR1,ZNF276,
MTMR3,KCNE2,
CABP7,CUL1,SL
C5A3,NEUROD4,
NUAK2,LOC1011
04855,HSPH1,TE
SPA1,AMDHD1,
ZMAT5,EPB41L3
,ARSG,LTA4H,Z
NF423,ZNF786,T
AC1,ZNF783,EXP
H5,BBS2,LYN,A
NKRD29,CDK18,
POU2F1,ZBTB14,
ASNS,MT4,SYT9,
MT3,GRIN2B,CO
Q6,GNAO1,MOV
10,SDK1,RCAN1,
ADCY10,DIAPH3
,MC1R,LOC1011
06129,KLHDC8A,
SPIRE2,STT3A,L
OC105614484,NF
1,CDK10,FAT2,A
TM,LOC1056096
17,RPS20,NF2,CE
NPBD1,FGF12,Z
NF410,VSX2,KL
HDC9,EZH2,CLI
C6,B4GALT3,US
PL1,LOC1011196

48,TCF25,MIDEA
S,LOC105616258,
GADL1,HMGB1,
RCSD1,SLC6A2,
PPCDC,CABIN1,
ADAMTS4,PPP3
CA,DSTYK,SIN3
A,ANXA6,MYO1
8A,MAN2C1,LOC
101113264,LOC1
01117184,INSL5,
LOC114118261,D
YNLT5,DNAI4,K
LF12,FCER1G,VP
S9D1,DCC,IFNG
R2,APOA2,WIPI1
,NAV2,RHOC,M
RPS6,MMP11,CD
HR3,CDH12,NDU
FS2,PFDN2,PIK3
C3,FAM20A,LOC
101114089,GART,
GAS8,LOC44342
3,LOC114117284,
LOC101109422,N
UFIP2,PLAG1,DE
RL3,NR1I3,CCD
C69,SRBD1,TYR
P1,LAMA3,NTN4
,PPM1J,LOC1141
08841,SPG7,COX
5A,EGFR,GNA13,
RPL13,LOC10111
7683,ASCC2,DED
D,MAP4K4,RRM
2,IL10RB,USP21,
AMFR,DEF8,TTC
39C,USF1,MOS,C
D2,NLRP14,NUD
T21,CD1,ZNF70,
TNIP1,NFIA,TAF
A3,CAPZA1,TAO
K1,ALOX5AP,CD
247,ZNF215,NEC

						TIN4,ZNF214,LOC101117691,LOC443441,LOC443442
MF	hydrolase activity, acting on carbon-nitrogen (but not peptide) bonds, in cyclic amides	0.000672	0.0468	0.0454	0.0000249	LOC101109035,AMDHD1,DPYS,DPEP1
MF	immune receptor activity	0.00132	0.0826	0.0802	0.000255	IFNAR2,IL1RL2,FCER1G,IL1R1,IL10RB,IFNGR2,IL1R2,IFNAR1
MF	cytokine receptor activity	0.00161	0.0917	0.089	0.000271	IFNAR2,IL1RL2,IL1R1,IL10RB,IFNGR2,IL1R2,IFNAR1
MF	intramolecular oxidoreductase activity	0.0019	0.0933	0.0906	0.000185	TMX1,MPI,LOC101111911,MIF,PDIA4
MF	solute:monoatomic cation symporter activity	0.00194	0.0933	0.0906	0.000338	SLC36A1,SLC36A3,SLC36A2,SLC16A1,LOC101108016,SLC6A2,SLC5A3
MF	L-proline transmembrane transporter activity	0.00229	0.102	0.0992	0.0000424	SLC36A1,SLC36A3,SLC36A2
MF	L-alanine transmembrane transporter activity	0.00292	0.108	0.104	0.0000632	SLC36A1,SLC36A3,SLC36A2
MF	glycine transmembrane transporter activity	0.00292	0.108	0.104	0.0000632	SLC36A1,SLC36A3,SLC36A2
MF	interleukin-1 receptor activity	0.00292	0.108	0.104	0.0000632	IL1RL2,IL1R1,IL1R2
MF	ion binding	0.00384	0.129	0.125	0.00297	CPNE7,MPI,CDH8,RIMS2,OGFOD1,CHEK1,UNC13B,LOC101117955,PRKCE,ENTPD5,HPCAL1,PIPOX,SDHC,PRKAR1A

						,DPEP1,MT1B,NEIL1,ZNF398,ZNF276,MTMR3,CABP7,NUAK2,HSPH1,AMDHD1,ZMAT5,ARSG,LTA4H,ZNF423,ZNF786,ZNF783,LYN,CDK18,ZBTB14,ASNS,MT4,SYT9,MT3,GRIN2B,COQ6,GNAO1,MOV10,ADCY10,STT3A,CDK10,FAT2,ATM,LOC105609617,ZNF410,B4GALT3,LOC101119648,MIDEAS,GADL1,HMGB1,SLC6A2,PPCDC,ADAMTS4,PPP3CA,DSTYK,ANXA6,MYO18A,MAN2C1,LOC101113264,LOC101117184,KLF12,APOA2,WIPI1,NAV2,RHOC,MMP11,CDHR3,CDH12,PIK3C3,FAM20A,GART,LOC101109422,PLAG1,NR1I3,TYRP1,PPM1J,SPG7,COX5A,EGFR,GNA13,LOC101117683,MAP4K4,RRM2,USP21,AMFR,DEF8,MOS,NLRP14,ZNF70,TAOK1,ALOX5AP,ZNF215,ZNF214,LOC101117691
MF	lipid binding	0.00404	0.129	0.125	0.00179	UNC13B,LOC443423,CPNE7,APOA2,WIPI1,HMGB

						1,LOC101110634,SYT9,RCSD1,CD1,ALOX5AP,ANXA6,NF1,LOC105614484,DPEP1,LOC101114089,LOC443441,LOC443442
MF	amide binding	0.00411	0.129	0.125	0.000837	CD1,LOC443423,LOC105614484,LOC101110634,LOC101114089,LOC443441,LOC443442
MF	alanine transmembrane transporter activity	0.00526	0.157	0.152	0.000162	SLC36A1,SLC36A3,SLC36A2
MF	lyase activity	0.00615	0.168	0.163	0.00176	ADCY10,HAL,ALOX5AP,ODC1,LOC101111911,GADL1,HMGB1,NEIL1,PPCDC
MF	transmembrane transporter binding	0.00617	0.168	0.163	0.000842	RIMS2,LYN,ANKRD29,KCNE2,SLC5A3
MF	small molecule binding	0.00723	0.189	0.183	0.00571	CPNE7,MPI,CDH8,RIMS2,OGFOD1,CHEK1,UNC13B,LOC101117955,PRKCE,ENTPD5,HPCAL1,PIPOX,SDHC,PRKAR1A,DPEP1,MT1B,NEIL1,ZNF398,ZNF276,MTMR3,CABP7,NUAK2,HSPH1,AMDHD1,ZMAT5,ARSG,LTA4H,ZNF423,ZNF786,ZNF783,LYN,CDK18,ZBTB14,ASNS,MT4,SYT9,MT3,GRIN2B,COQ6,GNAO1,MOV10,A

						DCY10,STT3A,CDK10,FAT2,ATM,LOC105609617,ZNF410,B4GALT3,LOC101119648,MIDEAS,GADL1,HMGB1,SLC6A2,PPCDC,ADAMTS4,PPP3CA,DSTYK,ANXA6,MYO18A,MAN2C1,LOC101113264,LOC101117184,KLF12,APOA2,WIPI1,NAV2,RHOC,MMP11,CDHR3,CDH12,NDUFS2,PIK3C3,FAM20A,GART,LOC101109422,PLAG1,NR1I3,TYRP1,PPM1J,SPG7,COX5A,EGFR,GNA13,LOC101117683,MAP4K4,RRM2,USP21,AMFR,DEF8,MOS,NLRP14,ZNF70,TAOK1,ALOX5AP,ZNF215,ZNF214,LOC101117691
MF	carboxylic acid transmembrane transporter activity	0.00919	0.23	0.223	0.00221	SLC36A1,SLC36A3,SLC36A2,SLC16A1,SLC16A6,LOC101108016,GRIN2B
MF	glutathione transferase activity	0.011	0.257	0.249	0.00118	LOC101111397,LOC101111664,ALOX5AP,LOC101118990
MF	symporter activity	0.0111	0.257	0.249	0.00277	SLC36A1,SLC36A3,SLC36A2,SLC16A1,LOC101108016,SLC6A2,SLC5A3

MF	protease binding	0.0172	0.367	0.357	0.00219	ADAMTS4,IL1R1,DERL3,MIF
MF	cation binding	0.0176	0.367	0.357	0.0133	B4GALT3,LOC101119648,MIDEAS,CPNE7,MPI,SLC6A2,CDH8,ADAMTS4,RIMS2,PPP3CA,OGFOD1,ANXA6,MAN2C1,LOC101113264,LOC101117184,UNC13B,LOC101117955,KLF12,PRKCE,HPCAL1,APOA2,SDHC,MMP11,CDH12,CDHR3,DPEP1,FAM20A,MT1B,NEIL1,GART,ZNF398,ZNF276,MTMR3,LOC101109422,CABP7,PLAG1,NR1I3,TYRP1,SPG7,COX5A,GNA13,NUAK2,AMDHD1,ZMAT5,LOC101117683,ARSG,LTA4H,ZNF786,ZNF423,ZNF783,RRM2,ZBTB14,USP21,AMFR,DEF8,MT4,SYT9,MT3,GRIN2B,GNAO1,ADCY10,ZNF70,STT3A,FAT2,ZNF215,ZNF214,LOC101117691,ZNF410
MF	phenylpyruvate tautomerase activity	0.0184	0.367	0.357	0.0000854	LOC101111911,MIF
MF	interleukin-1, type I, activating receptor activity	0.0184	0.367	0.357	0.0000854	IL1RL2,IL1R1
MF	metal ion binding	0.0188	0.367	0.357	0.0142	B4GALT3,LOC10

						1119648,MIDEAS,CPNE7,MPI,SLC6A2,CDH8,ADAMTS4,RIMS2,PPP3CA,OGFOD1,ANXA6,MAN2C1,LOC101113264,LOC101117184,UNC13B,LOC101117955,KLF12,PRKCE,HPCAL1,SDHC,MMP11,CDH12,CDHR3,DPEP1,FAM20A,MT1B,NEIL1,GART,ZNF398,ZNF276,MTMR3,LOC101109422,CABP7,PLAG1,NR1I3,TYRP1,SPG7,COX5A,GNA13,NUAK2,AMDHD1,ZMAT5,LOC101117683,ARSG,LTA4H,ZNF786,ZNF423,ZNF783,RRM2,ZBTB14,USP21,AMFR,DEF8,MT4,SYT9,MT3,GRIN2B,GNAO1,ADCY10,ZNF70,STT3A,FAT2,ZNF215,ZNF214,LOC101117691,ZNF410
MF	zinc ion binding	0.0199	0.378	0.367	0.013	ZNF276,MTMR3,PLAG1,MIDEAS,NR1I3,MPI,SPG7,ADAMTS4,RIMS2,ZMAT5,LOC101117683,LTA4H,ZNF786,ZNF423,LOC101113264,LOC101117184,ZNF783,UNC13B,LO

						C101117955,KLF12,ZBTB14,PRKCE,AMFR,DEF8,MT3,MMP11,ZNF70,DPEP1,ZNF215,MT1B,NEIL1,LOC101117691,ZNF214,ZNF410,ZNF398
MF	protein serine/threonine kinase activity	0.0211	0.388	0.377	0.00904	MOS,CDK18,DSTYK,NUAK2,PRKAR1A,TAOK1,PRKCE,CHEK1,LOC101117683,CDK10,ATM,MAP4K4
MF	proton transmembrane transporter activity	0.0254	0.455	0.442	0.00656	SLC36A1,SLC36A3,SLC36A2,SLC16A1,LOC101108016,NDUFS2
MF	anion binding	0.0286	0.497	0.482	0.0201	LOC101109422,GADL1,PPM1J,HMGB1,SPG7,PPCDC,EGFR,GNA13,DSTYK,HSPH1,NUAK2,OGFOD1,CHEK1,ANXA6,MYO18A,LOC101117683,LOC101113264,MAP4K4,LYN,CDK18,ENTPD5,PRKCE,PIPOX,ASNS,WIPI1,NAV2,RHOC,SYT9,COQ6,GNAO1,MOS,NLRP14,ADCY10,MOV10,PRKAR1A,TAOK1,ALOX5AP,CDK10,LOC105609617,PIK3C3,ATM,FAM20A,GART
MF	protein kinase activity	0.0357	0.542	0.527	0.0178	LYN,CDK18,PRKCE,EGFR,MOS,DSTYK,NUAK2,P

						RKAR1A,TAOK1,CHEK1,CDK10,LOC101117683,ATM,MAP4K4
MF	protein binding	0.0358	0.542	0.527	0.0293	CLIC6,USPL1,TCF25,LOC105616258,HMGB1,RCSD1,SLC6A2,CDH8,CABIN1,ADAMTS4,RIMS2,PPP3CA,RUSC2,SIN3A,ANXA6,MYO18A,POGLUT3,INSL5,PKNOX2,UNC13B,LOC114118261,DYNLT5,DNAI4,IFNAR2,VPS9D1,WSB1,DCC,IL1R1,IFNGR2,IL1R2,APOA2,WIPI1,MIF,NAV2,PRKAR1A,CDH12,PFDN2,GAS8,IFNAR1,MTMR3,KCNE2,DERL3,CCDC69,LAMA3,CUL1,NTN4,SLC5A3,EGFR,GNA13,NEUROD4,LOC101104855,HSPH1,TESPA1,EPB41L3,ASCC2,TAC1,DEDD,ZNF783,EXPH5,BBS2,LYN,ANKRD29,ZBTB14,IL10RB,AMFR,SYT9,TTC39C,USF1,GNAO1,CD2,NLRP14,SDK1,DIAPH3,MC1R,TNIP1,TAFA3,KLHDC8A,CAPZA1,TAOK1,SPIRE2,ATM,RPS20,NF2,ZNF215,CD247,

						NECTIN4,FGF12,KLHDC9,EZH2
MF	lactate:proton symporter activity	0.0364	0.542	0.527	0.000506	SLC16A1,LOC101108016
MF	type I interferon receptor activity	0.0364	0.542	0.527	0.000506	IFNAR2,IFNAR1
MF	intramolecular oxidoreductase activity, interconverting keto- and enol-groups	0.0364	0.542	0.527	0.000506	LOC101111911,MIF
MF	interferon receptor activity	0.0364	0.542	0.527	0.000506	IFNAR2,IFNAR1
MF	amino acid:monoatomic cation symporter activity	0.0434	0.632	0.614	0.00447	SLC36A1,SLC36A3,SLC36A2
MF	interleukin-1 binding	0.0453	0.644	0.625	0.000838	IL1R1,IL1R2
MF	protein-containing complex binding	0.0467	0.65	0.631	0.0249	LYN,FCER1G,APOA2,HMGB1,LOC114108841,RCSD1,CABIN1,GNAO1,LOC105602976,GNA13,CAPZA1,ANXA6,MYO18A,EZH2,PKNOX2
MF	phosphotransferase activity, alcohol group as acceptor	0.0506	0.689	0.669	0.0273	LYN,CDK18,PRKCE,EGFR,MOS,DSTYK,NUAK2,PRKAR1A,TAOK1,CHEK1,CDK10,LOC101117683,PIK3C3,ATM,MAP4K4
MF	[pyruvate dehydrogenase (acetyl-transferring)]-phosphatase activity	0.0541	0.71	0.689	0.00125	PPM1J,RHOC
MF	modified amino acid binding	0.0563	0.71	0.689	0.0114	ANXA6,DPEP1,HMGB1,SYT9
MF	transition metal	0.0583	0.71	0.689	0.0416	ZNF276,MTMR3,

	ion binding					LOC101119648,PLAG1,MIDEAS,NR1I3,MPI,SPG7,ADAMTS4,RIMS2,OGFOD1,ZMAT5,LOC101117683,LTA4H,ZNF786,ZNF423,LOC101113264,LOC101117184,ZNF783,UNC13B,LOC101117955,KLF12,ZBTB14,PRKCE,AMFR,DEF8,MT3,MMP11,ZNF70,DPEP1,ZNF215,MT1B,NEIL1,LOC101117691,ZNF214,ZNF410,ZNF398
MF	heterocyclic compound binding	0.0583	0.71	0.689	0.0423	LOC101109422,GADL1,PPM1J,SPG7,PPCDC,EGFR,GNA13,DSTYK,HSPH1,NUAK2,OGFOD1,CHEK1,ANXA6,MYO18A,LOC101117683,LOC101113264,MAP4K4,LYN,CDK18,ENTPD5,PRKCE,PIPOX,ASNS,NAV2,RHOC,COQ6,GNAO1,MOS,NLRP14,ADCY10,MOV10,PRKAR1A,TAOK1,CDK10,NDUFS2,LOC105609617,PIK3C3,ATM,FAM20A,GART
MF	hydrolase activity, acting on carbon-nitrogen (but not peptide)	0.0589	0.71	0.689	0.016	LOC101109035,AMDHD1,DPYS,DPEP1,NIT1

	bonds				
MF	neutral L-amino acid transmembrane transporter activity	0.0597	0.71	0.689	0.00737	SLC36A1,SLC36A3,SLC36A2
MF	actin binding	0.0616	0.71	0.689	0.0284	DIAPH3,CAPZA1,EPB41L3,SPIRE2,ANXA6,MYO18A,NF2,RCSD1,SLC6A2,PKNOX2
MF	metallopeptidase activity	0.0618	0.71	0.689	0.0229	ADAMTS4,MMP11,DPEP1,AMZ2,LTA4H,SPG7,LOC101113264
MF	phospholipid binding	0.0628	0.71	0.689	0.0275	UNC13B,CPNE7,ANXA6,APOA2,DPEP1,WIPI1,HMGB1,SYT9,RCSD1
MF	nucleotide binding	0.0655	0.71	0.689	0.0475	LOC101109422,PPM1J,SPG7,PPCDC,EGFR,GNA13,DSTYK,HSPH1,NUAK2,CHEK1,ANXA6,MYO18A,LOC101117683,LOC101113264,MAP4K4,LYN,CDK18,ENTPD5,PRKCE,PIPOX,ASNS,NAV2,RHOC,COQ6,GNAO1,MOS,NLRP14,ADCY10,MOV10,PRKAR1A,TAOK1,CDK10,NDUFS2,LOC105609617,PIK3C3,ATM,FAM20A,GART
MF	ribonucleotide binding	0.0665	0.71	0.689	0.0474	LOC101109422,PPM1J,SPG7,PPCDC,EGFR,GNA13,DSTYK,HSPH1,NUAK2,CHEK1,A

						NXA6,MYO18A,LOC101117683,LOC101113264,MAP4K4,LYN,CDK18,ENTPD5,PRKCE,ASNS,NAV2,RHOC,GNAO1,MOS,NLRP14,ADCY10,MOV10,PRKAR1A,TAOK1,CDK10,LOC105609617,PIK3C3,ATM,FAM20A,GART
MF	protein serine/threonine phosphatase activity	0.0669	0.71	0.689	0.0145	PPP3CA,MTMR3,PPM1J,RHOC
MF	transferase activity, transferring alkyl or aryl (other than methyl) groups	0.0669	0.71	0.689	0.0145	LOC101111397,LOC101111664,ALOX5AP,LOC101118990
MF	nucleoside phosphate binding	0.0708	0.733	0.712	0.0516	LOC101109422,PPM1J,SPG7,PPCDC,EGFR,GNA13,DSTYK,HSPH1,NUAK2,CHEK1,ANXA6,MYO18A,LOC101117683,LOC101113264,MAP4K4,LYN,CDK18,ENTPD5,PRKCE,PIPOX,ASNS,NAV2,RHOC,COQ6,GNAO1,MOS,NLRP14,ADCY10,MOV10,PRKAR1A,TAOK1,CDK10,NDUFS2,LOC105609617,PIK3C3,ATM,FAM20A,GART
MF	lactate transmembrane	0.0714	0.733	0.712	0.0023	SLC16A1,LOC101108016

	transporter activity					
MF	adenyl ribonucleotide binding	0.0765	0.773	0.75	0.0525	LOC101109422,SPG7,EGFR,DSTYK,HSPH1,NUAK2,CHEK1,MYO18A,LOC101117683,LOC101113264,MAP4K4,LYN,CDK18,ENTPD5,PRKCE,ASNS,NAV2,MOS,NLRP14,ADCY10,MOV10,PRKAR1A,TAOK1,CDK10,PIK3C3,ATM,FAM20A,GART
MF	adenyl nucleotide binding	0.0808	0.803	0.78	0.0562	LOC101109422,SPG7,EGFR,DSTYK,HSPH1,NUAK2,CHEK1,MYO18A,LOC101117683,LOC101113264,MAP4K4,LYN,CDK18,ENTPD5,PRKCE,ASNS,NAV2,MOS,NLRP14,ADCY10,MOV10,PRKAR1A,TAOK1,CDK10,NDUFS2,PIK3C3,ATM,FAM20A,GART
MF	secondary active transmembrane transporter activity	0.087	0.851	0.826	0.0352	SLC36A1,SLC36A3,SLC36A2,SLC16A1,LOC101108016,SLC6A2,SLC5A3
MF	purine ribonucleotide binding	0.0892	0.851	0.826	0.0648	LOC101109422,PPM1J,SPG7,EGFR,GNA13,DSTYK,HSPH1,NUAK2,CHEK1,ANXA6,MYO18A,LOC101117683,LOC101113264,MAP4K4,

						LYN,CDK18,ENTPD5,PRKCE,ASNS,NAV2,RHOC,GNAO1,MOS,NLRP14,ADCY10,MOV10,PRKAR1A,TAOK1,CDK10,LOC105609617,PIK3C3,ATM,FAM20A,GART
MF	catalytic activity	0.0897	0.851	0.826	0.0763	B4GALT3,USPL1,PIGO,LOC101119648,DPYS,LOC101116336,MPI,GADL1,HMGB1,PPCDC,ADAMTS4,PPP3CA,DSTYK,OGFOD1,CHEK1,MYO18A,MAN2C1,LOC101118990,LOC101113264,POGLUT3,WSB1,IL1R1,ENTPD5,PRKCE,PIPOX,SDHC,MIF,NAV2,RHOC,PTGR2,PDIA4,MMP11,TMX1,PRKAR1A,UFC1,DPEP1,NDUFS2,PIK3C3,NEIL1,GART,LOC101116756,LOC101118978,MTMR3,LOC101109422,TYRP1,ODC1,LOC101111664,B3GLCT,PPM1J,SPG7,PPOX,EGFR,GNA13,IL1RL2,NUAK2,AMDHD1,TGS1,TMEM68,LOC101117683,AMZ2,ARSG,LTA4H,MAP4K4,GGT5,LOC10

						1111397,LYN,CDK18,ANKRD29,LOC101109035,RRM2,USP21,AMFR,LOC101111911,ASNS,COQ6,GNAO1,MOS,MOV10,ADCY10,ALDH6A1,HAL,TAOK1,ALOX5AP,STT3A,CDK10,ATM,LOC105609617,NIT1,EZH2
MF	purine nucleotide binding	0.0931	0.868	0.843	0.0682	LOC101109422,PPM1J,SPG7,EGFR,GNA13,DSTYK,HSPH1,NUAK2,CHEK1,ANXA6,MYO18A,LOC101117683,LOC101113264,MAP4K4,LYN,CDK18,ENTPD5,PRKCE,ASNS,NAV2,RHOC,GNAO1,MOS,NLRP14,ADCY10,MOV10,PRKAR1A,TAOK1,CDK10,NDUFS2,LOC105609617,PIK3C3,ATM,FAM20A,GART
MF	carboxy-lyase activity	0.0943	0.868	0.843	0.0152	ODC1,GADL1,PPCDC
MF	dopamine receptor binding	0.0969	0.87	0.845	0.00444	CLIC6,GNA13
MF	carbohydrate derivative binding	0.0985	0.87	0.845	0.0733	LOC101109422,PPM1J,HMGB1,SPG7,PPCDC,EGFR,GNA13,DSTYK,HSPH1,NUAK2,CHEK1,ANXA6,MYO18A,LOC101117683,LOC101113264,MAP4K4,LYN,

						CDK18,ENTPD5,PRKCE,ASNS,NAV2,RHOC,GNAO1,MOS,NLRP14,ADCY10,MOV10,PRKAR1A,TAOK1,DPEP1,CDK10,LOC105609617,PIK3C3,ATM,FAM20A,GART
MF	ATP binding	0.0987	0.87	0.845	0.0688	LOC101109422,SPG7,EGFR,DSTYK,HSPH1,NUAK2,CHEK1,MYO18A,LOC101117683,LOC101113264,MAP4K4,LYN,CDK18,ENTPD5,PRKCE,ASNS,NAV2,MOS,NLRP14,ADCY10,MOV10,TAOK1,CDK10,PIK3C3,ATM,FAM20A,GART
