# Supplementary material for: Genome-wide association studies of body weight, body conformation traits and coat color in Gamba sheep
Source: Anim Biosci. 2026 Jun 15;39(10):260295. doi: 10.5713/ab.260295 (PMC13662935; doi:10.5713/ab.260295)
Supplement: Supplementary file 12 [file ab-260295-Supplementary-20.pdf]

Supplement 20. KEGG pathway enrichment analysis of candidate genes.

Category	Term	P-Value	Bonferroni	FDR	User Ids
KEGG	Hematopoietic cell lineage	0.0000173	0.00402	0.00403	CD2,CD1,LOC443423,IL1R1,IL1R2,LOC105614484,LOC101110634,LOC101114089,LOC443441,LOC443442
KEGG	Amoebiasis	0.0000387	0.00898	0.00451	CD1,LOC443423,IL1R1,IL1R2,LAMA3,LOC105614484,LOC101110634,LOC101114089,LOC443441,LOC443442
KEGG	Drug metabolism - other enzymes	0.00143	0.284	0.111	LOC101111397,LOC101116756,RRM2,DPYS,LOC101111664,LOC101116336,LOC101118990
KEGG	Glutathione metabolism	0.00344	0.552	0.2	GGT5,LOC101111397,RRM2,LOC101111664,ODC1,LOC101118990
KEGG	Tight junction	0.00527	0.708	0.246	CD1,LOC443423,PRKCE,LOC105614484,LOC101110634,NF2,LOC101114089,LOC443441,LOC443442
KEGG	Metabolic pathways	0.0295	0.999	1.00e+0	B4GALT3,MTMR3,PIGO,DPYS,TYRP1,ODC1,LOC101111664,MPI,GADL1,PPOX,COX5A,PPCDC,AMDHD1,LOC101108778,LTA4H,LOC101118990,GGT5,LOC101111397,LOC101109035,RRM2,ENTPD5,LOC114110816,PIPOX,ASNS,SDHC,MIF,COQ6

					,ADCY10,ALDH6A1,HAL,STT3A,NDUFS2,PIK3C3,GART,EZH2
KEGG	Pantothenate and CoA biosynthesis	0.0391	1.00e+0	1.00e+0	DPYS,GADL1,PPCDC
KEGG	Chemical carcinogenesis - reactive oxygen species	0.0457	1.00e+0	1.00e+0	LOC101111397,LOC101111664,LOC114110816,NDUFS2,LOC101108778,SDHC,LOC101118990,COX5A,EGFR
KEGG	Protein processing in endoplasmic reticulum	0.0489	1.00e+0	1.00e+0	LOC105612995,HSPH1,AMFR,DERL3,STT3A,CUL1,PDIA4
KEGG	Natural killer cell mediated cytotoxicity	0.0546	1.00e+0	1.00e+0	IFNAR2,PPP3CA,FCER1G,IFNGR2,CD247,IFNAR1
KEGG	Necroptosis	0.0555	1.00e+0	1.00e+0	IFNAR2,USP21,IFNGR2,CHMP1A,HMGB1,IFNAR1,SPATA2L
KEGG	beta-Alanine metabolism	0.0937	1.00e+0	1.00e+0	ALDH6A1,DPYS,GADL1
KEGG	Mineral absorption	0.0954	1.00e+0	1.00e+0	LOC101117955,MT1B,LOC101117691,LOC101117184
