# Supplementary material for: Genome-wide association studies of body weight, body conformation traits and coat color in Gamba sheep
Source: Anim Biosci. 2026 Jun 15;39(10):260295. doi: 10.5713/ab.260295 (PMC13662935; doi:10.5713/ab.260295)
Supplement: Supplementary file 14 [file ab-260295-Supplementary-5.pdf]

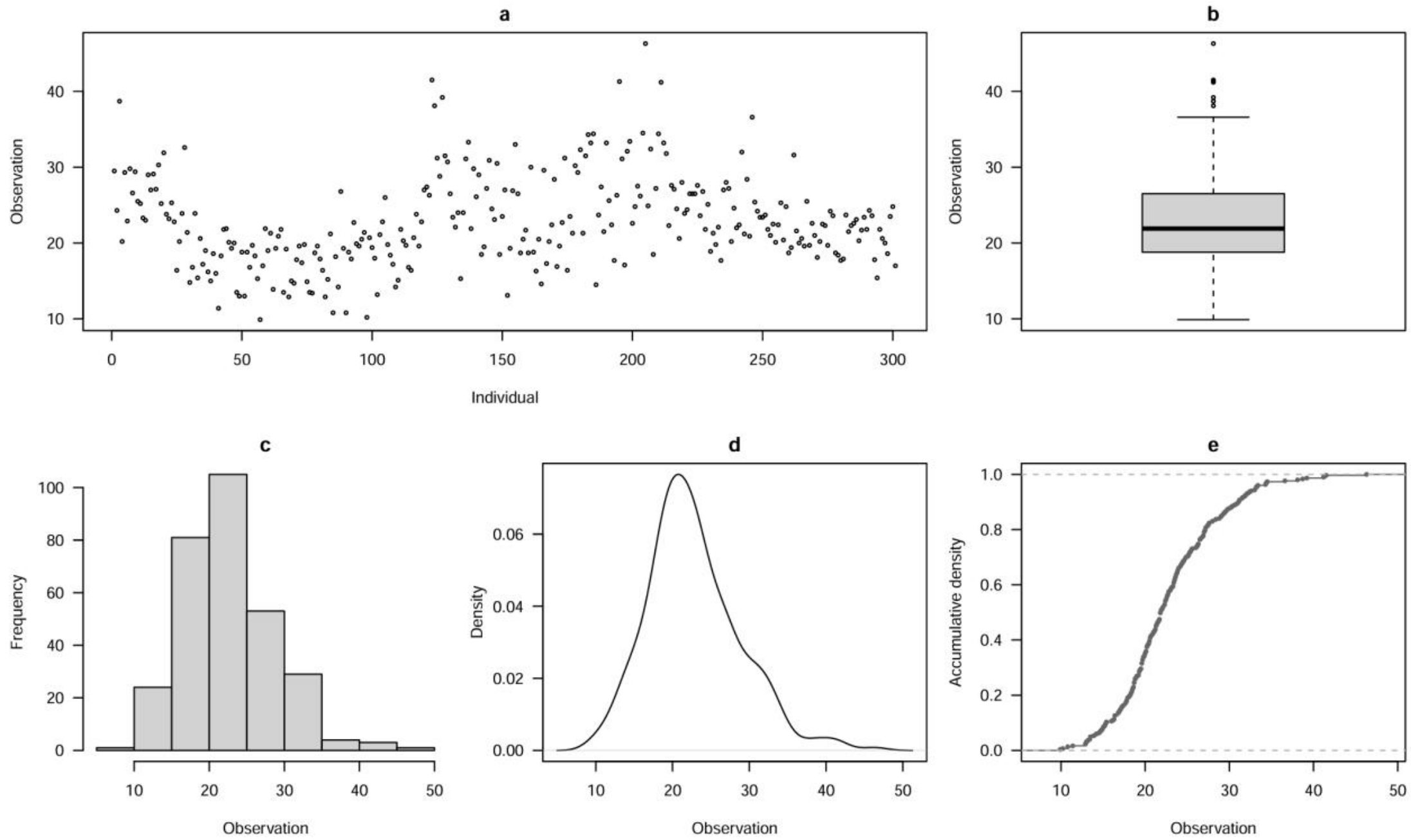


**Supplement 5.** Phenotypic distribution of body weight (BW) in Gamba sheep. (a) Discrete distribution plot;(b) Boxplot;(c) Histogram;(d) Kernel density curve;(e) Cumulative distribution curve.
