# Supplementary material for: Genome-wide association studies of body weight, body conformation traits and coat color in Gamba sheep
Source: Anim Biosci. 2026 Jun 15;39(10):260295. doi: 10.5713/ab.260295 (PMC13662935; doi:10.5713/ab.260295)

Supplement 8. Kinship heatmap of 301 Gamba sheep individuals.

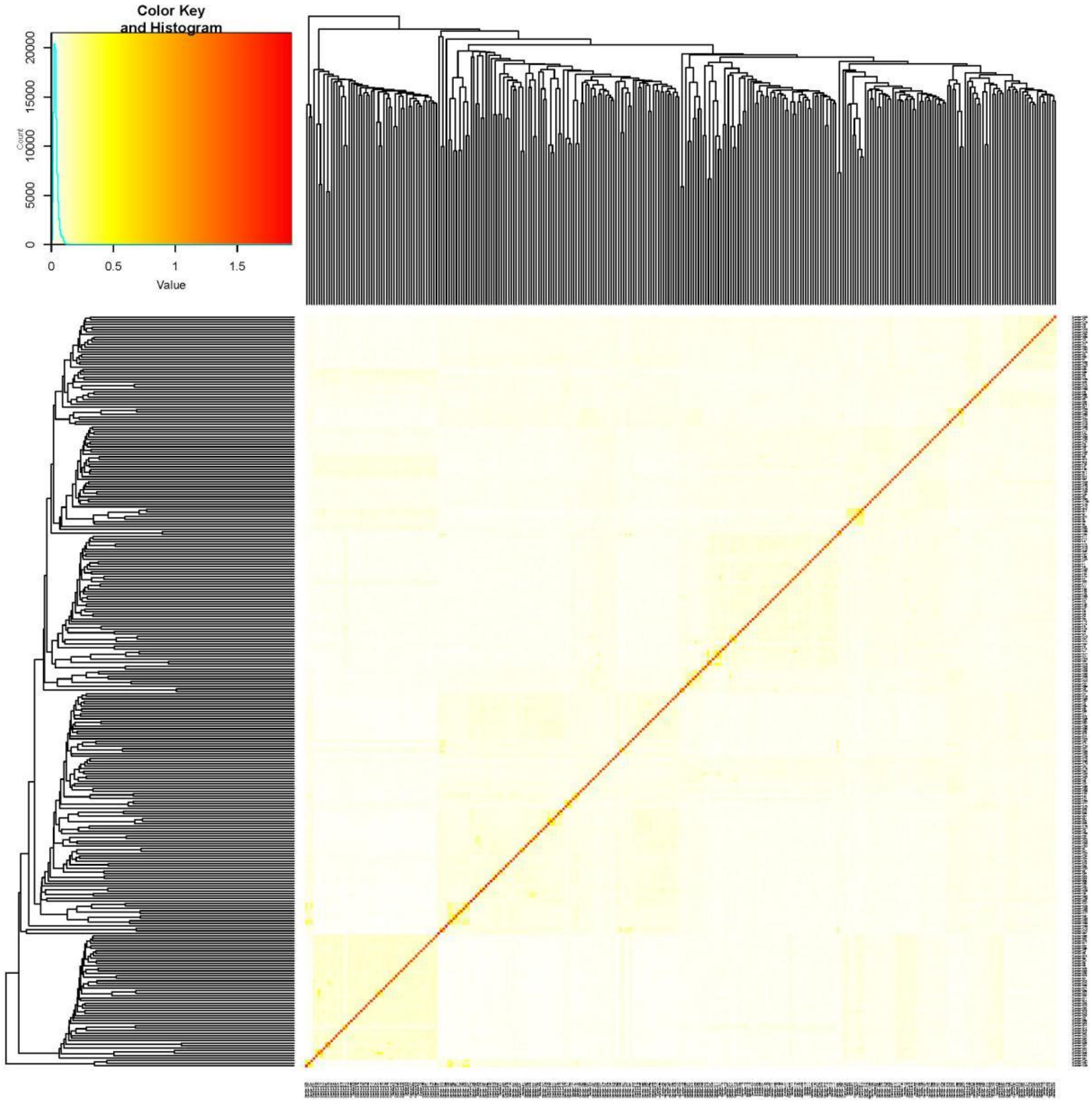

Supplement: Supplementary file 17 [file ab-260295-Supplementary-8.pdf]
