# Supplementary material for: Genome-wide association studies of body weight, body conformation traits and coat color in Gamba sheep
Source: Anim Biosci. 2026 Jun 15;39(10):260295. doi: 10.5713/ab.260295 (PMC13662935; doi:10.5713/ab.260295)
Supplement: Supplementary file 18 [file ab-260295-Supplementary-9.pdf]

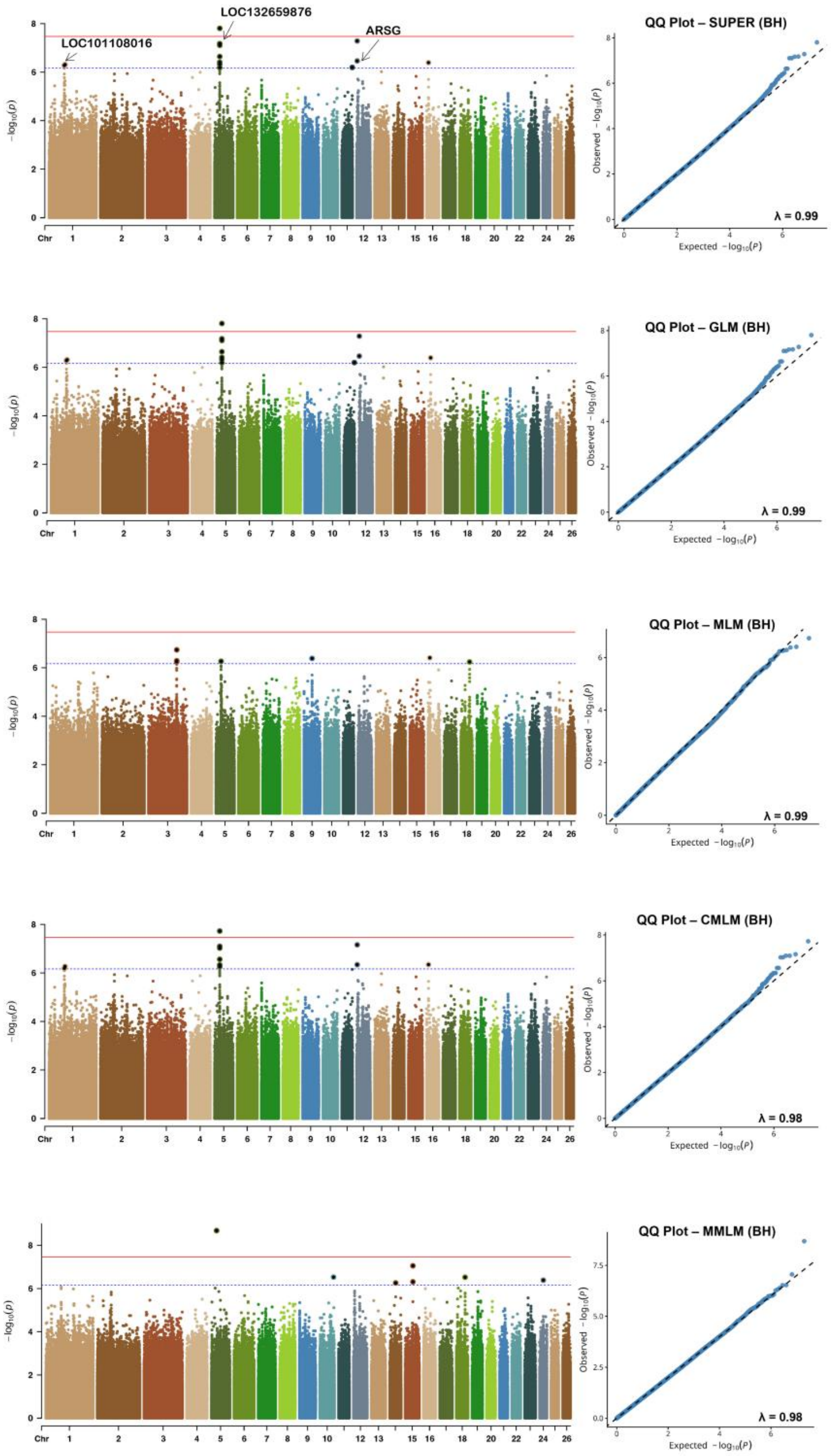

Supplement 9. Comparison of GWAS models for body height (BH) in Gamba sheep.

Note: Manhattan plots are shown on the left, and Q-Q plots are shown on the right. Results for the *SUPER, CMLM, GLM, MLM*, and *MLMM* models are displayed from top to bottom, respectively. The red solid and blue dashed horizontal lines in the Manhattan plots represent the strict genome-wide significance threshold ($P = 3.42 \times 10^{-8}$) and the suggestive significance threshold ($P = 6.84 \times 10^{-7}$), respectively. Annotated gene symbols indicate significantly associated loci.
